# Supplementary material for: TPR is required for cytoplasmic chromatin fragment formation during senescence
Source: eLife. 2024 Dec 3;13:e101702. doi: 10.7554/eLife.101702 (PMC11666244; doi:10.7554/eLife.101702)
Supplement: Figure 2—source data 1. — Median NF-κB nucleocytoplasmic ratios (n/c) and number of cells analysed for day 8 (d8) STOP or RAS cells subject to knockdown with control (CTRL) or TPR siRNAs, and for experiments where these cells were treated with conditioned media (CM) from either STOP or RAS cells. Kruskal-Wallis testing was used to determine statistical significance for each replicate (p-value in parentheses) followed by Dunn’s post hoc testing. p-values after Benjamini and Hochberg correction. [file elife-101702-fig2-data1.docx]

| **Figure 2B and Figure 2- figure supplement 1 A** | | | | | | | |
| --- | --- | --- | --- | --- | --- | --- | --- |
| **Cells** | **siRNA** | **NF-κB** **n/c** | **No. of cells** | **P value** | **NF-κB n/c** | **No. of cells** | **P value** |
|  |  | Replicate 1 (p< 2.2E-16) | | | Replicate 2 (p< < 2.2E-16) | | |
| STOP | siCTRL | 0.038 | 115 | 0.112 | 0.025 | 190 | 7.26E-07 |
|  | siTPR | 0.058 | 105 |  | 0.043 | 148 |  |
| RAS | siCTRL | 0.126 | 101 | 1.39E-06 | 0.059 | 101 | 0.03225 |
|  | siTPR | 0.046 | 128 |  | 0.045 | 128 |  |
| STOP | siCTRL | 0.038 | 115 | 8.90E-08 | 0.025 | 190 | 6.67E-29 |
| RAS |  | 0.126 | 101 |  | 0.059 | 101 |  |

| **Figure 2F and Figure 2- figure supplement 1 D** | | | | | | | | |
| --- | --- | --- | --- | --- | --- | --- | --- | --- |
| **Cells** | **siRNA** | **CM** | **NF-κB n/c** | **No. of cells** | **P value** | **NF-κB n/c** | **No. of cells** | **P value** |
|  |  |  | Replicate 1 (p< 2.2E-16) | | | Replicate 2 (p< 2.2E-16) | | |
| STOP | siCTRL | STOP | 0.981 | 4525 | 0.00E+00 | 0.959 | 786 | 0.00E+00 |
|  | siCTRL | RAS | 1.583 | 3397 |  | 1.524 | 973 |  |
| STOP | siTPR | STOP | 1.010 | 1972 | 0.00E+00 | 0.974 | 643 | 1.21E-245 |
|  | siTPR | RAS | 1.641 | 2438 |  | 1.432 | 738 |  |
| RAS | siCTRL | STOP | 1.192 | 801 | 1.41E-20 | 1.067 | 328 | 3.79E-06 |
|  | siCTRL | RAS | 1.473 | 563 |  | 1.166 | 358 |  |
| RAS | siTPR | STOP | 1.071 | 679 | 6.57E-20 | 1.079 | 416 | 4.73E-11 |
|  | siTPR | RAS | 1.321 | 524 |  | 1.188 | 439 |  |

**Figure 2 – source data 1. Quantification of NF-κB nucleocytoplasmic ratios and statistical analysis for data in Figure 2B and F, and for biological replicates in Figure 2- figure supplement 1A and D.** Median NF-κB nucleocytoplasmic ratios (n/c) and number of cells analysed for d8 STOP or RAS cells subject to knockdown with control (CTRL) or TPR siRNAs, and for experiments where these cells were treated with conditioned media (CM) from either STOP or RAS cells. Kruskal-Wallis testing was used to determine statistical significance for each replicate (p value in parentheses) followed by Dunn post-hoc testing. P values after Benjamini and Hochberg correction.
