# Supplementary material for: TPR is required for cytoplasmic chromatin fragment formation during senescence
Source: eLife. 2024 Dec 3;13:e101702. doi: 10.7554/eLife.101702 (PMC11666244; doi:10.7554/eLife.101702)
Supplement: Figure 2—source data 2. [file elife-101702-fig2-data2.pdf]

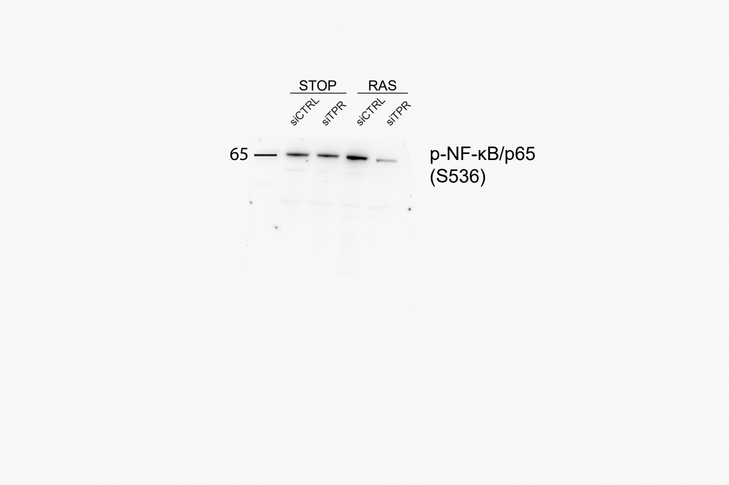
STOP
RAS
siCTRL
siTPR
siCTRL
siTPR
65
p-NF-κB/p65
(S536)

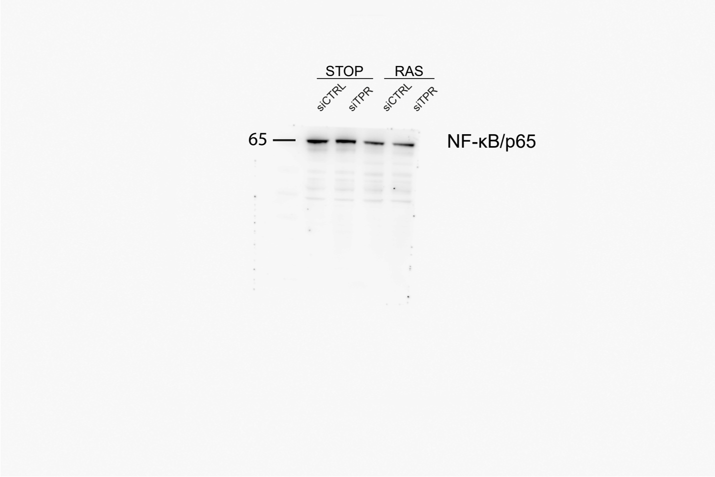
STOP
RAS
siCTRL
siTPR
siCTRL
siTPR
65
NF-κB/p65

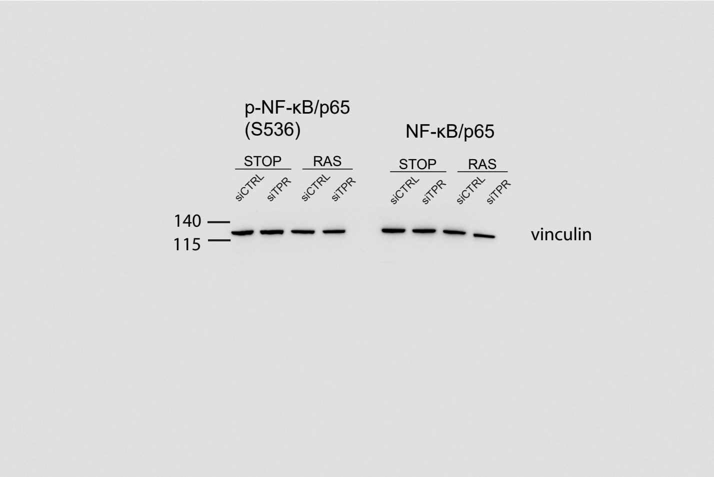
p-NF-κB/p65
(S536)
NF-κB/p65
STOP
RAS
siCTRL
siTPR
siCTRL
siTPR
STOP
RAS
siCTRL
siTPR
siCTRL
siTPR
140
115
vinculin

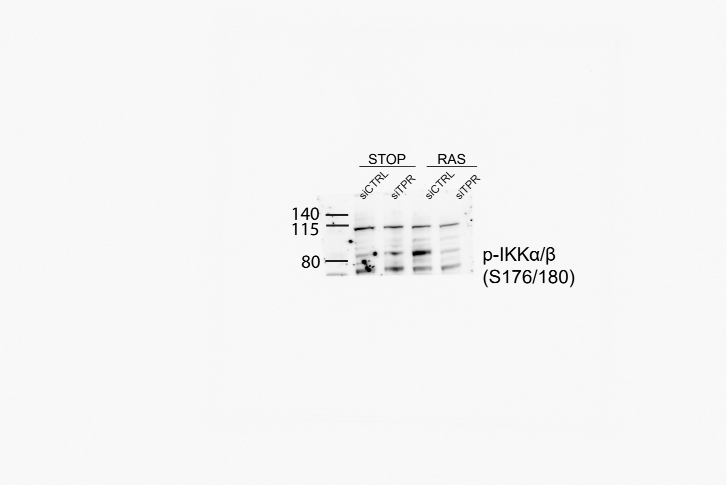
STOP
RAS
siCTRL
siTPR
siCTRL
siTPR
140
115
80
p-IKKα/β
(S176/180)

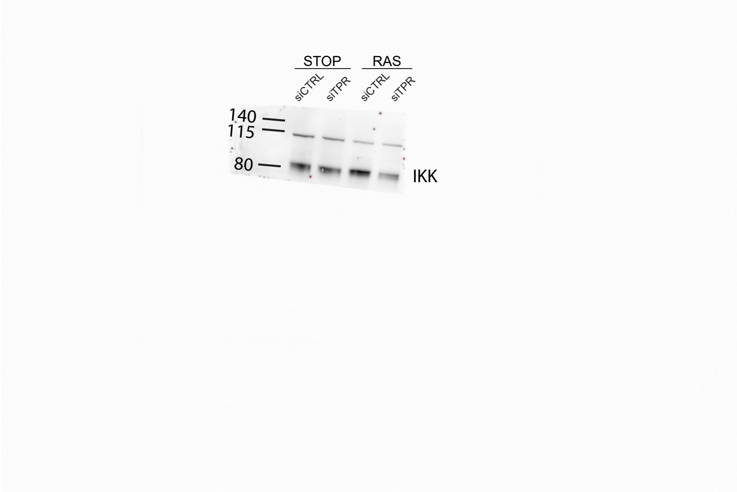
STOP
RAS
siCTRL
siTPR
siCTRL
siTPR
140
115
80
IKK

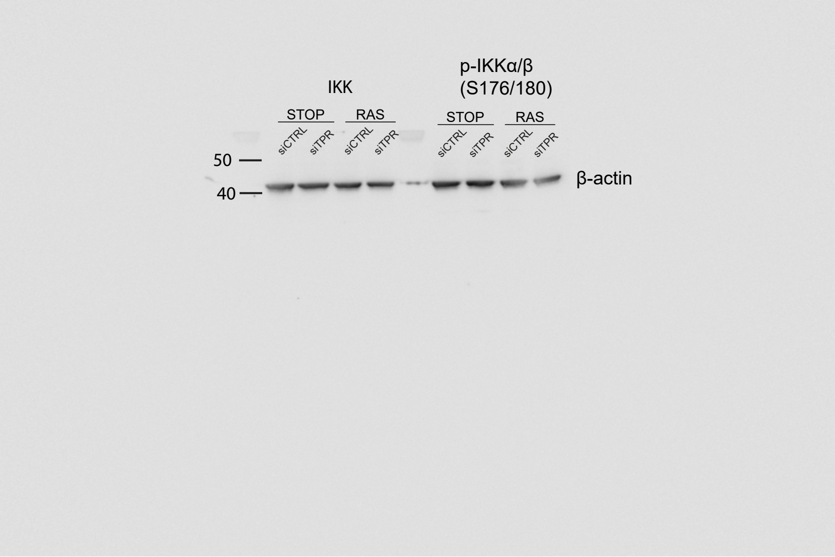
IKK
p-IKKα/β
(S176/180)
STOP
RAS
siCTRL
siTPR
siCTRL
siTPR
STOP
RAS
siCTRL
siTPR
siCTRL
siTPR
50
40
β-actin
