# Supplementary figures and images for: TPR is required for cytoplasmic chromatin fragment formation during senescence
Source: eLife. 2024 Dec 3;13:e101702. doi: 10.7554/eLife.101702 (PMC11666244; doi:10.7554/eLife.101702)

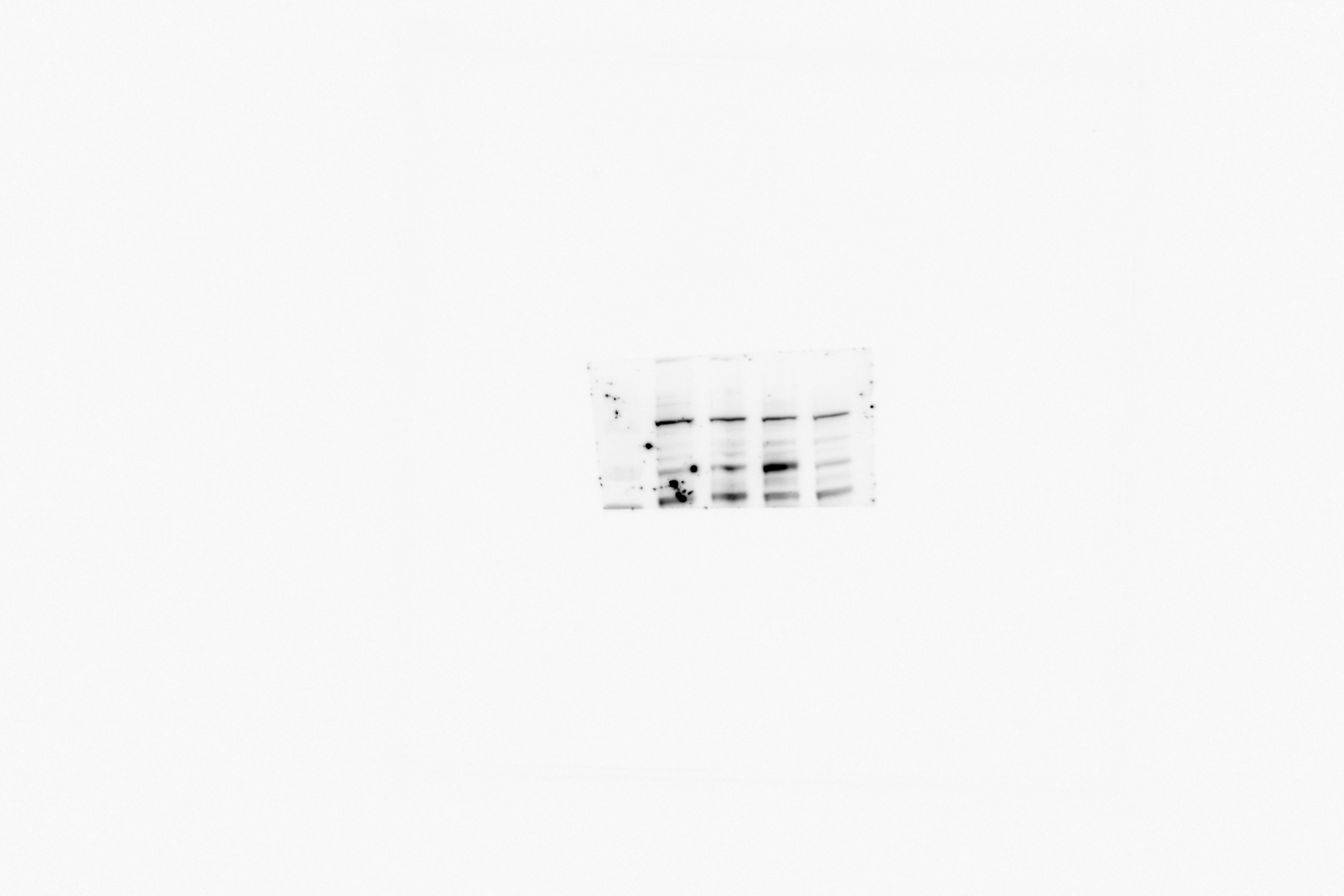

Supplement: Figure 2—source data 3. [file elife-101702-fig2-data3.zip › p-IKK day 8.tif]

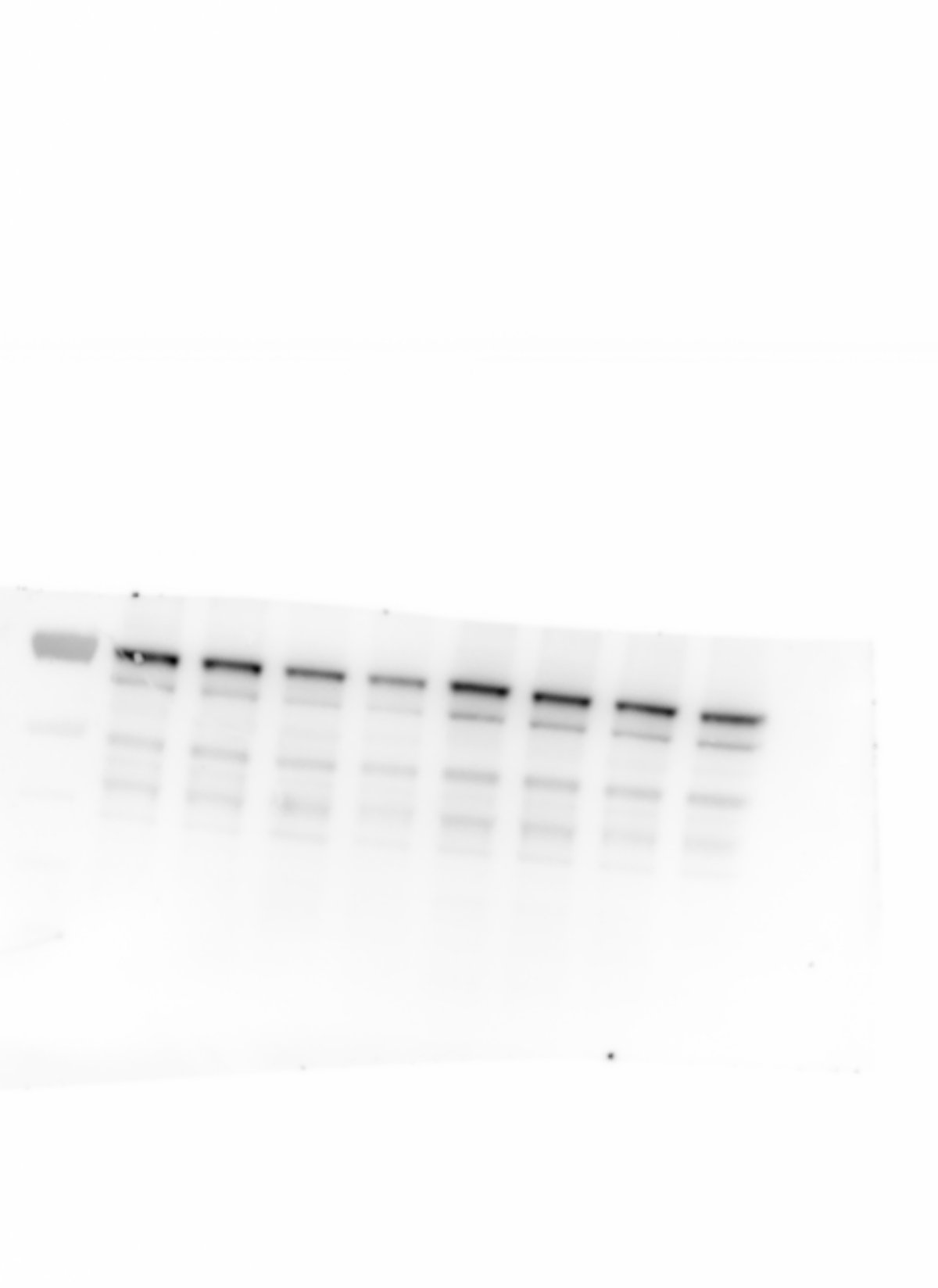

Supplement: Figure 2—source data 3. [file elife-101702-fig2-data3.zip › p65_23 20210721_124831_Ch_Chemi.jpg]

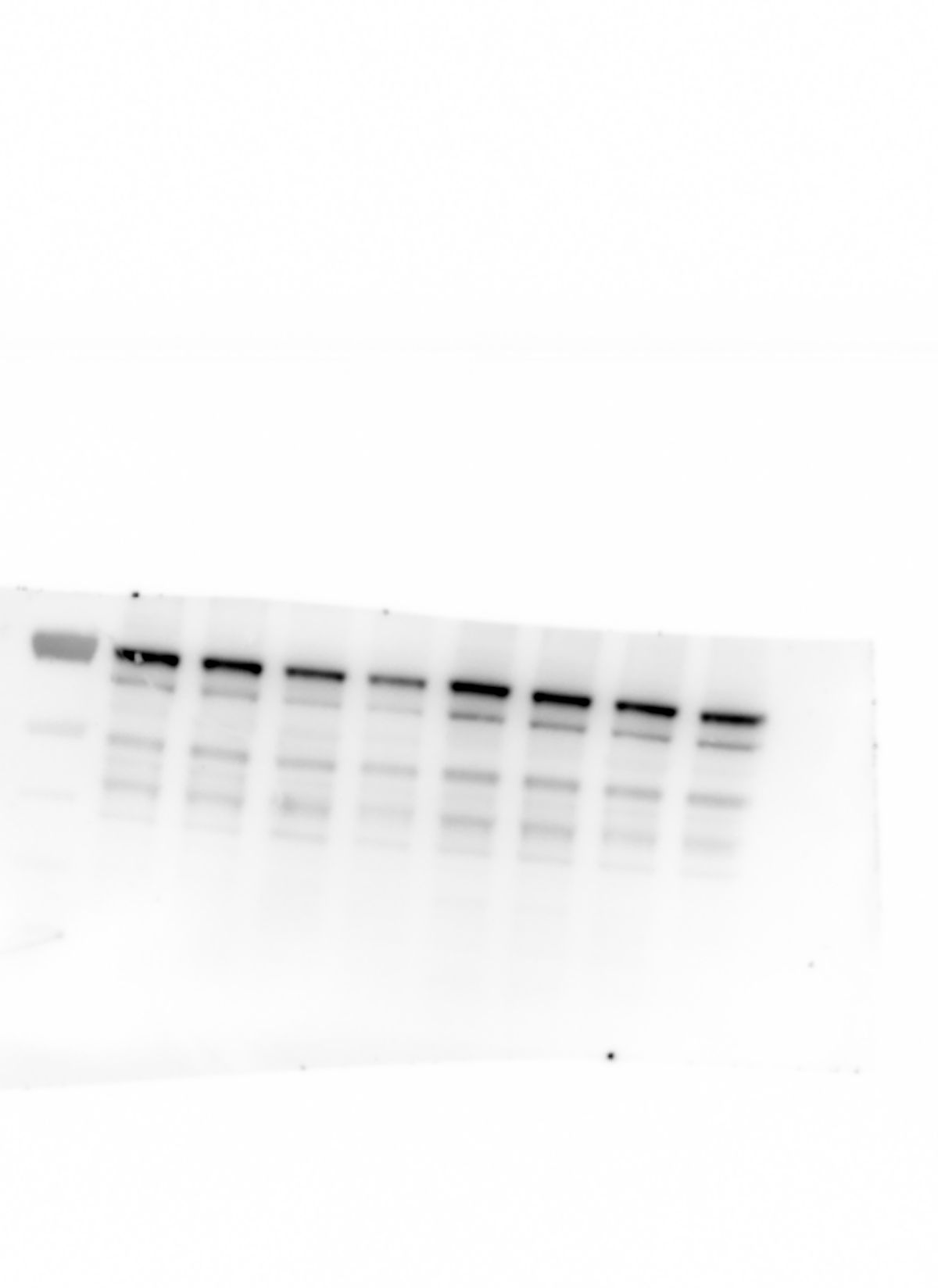

Supplement: Figure 2—source data 3. [file elife-101702-fig2-data3.zip › p65_23 20210721_124831_Ch_Chemi.tif]

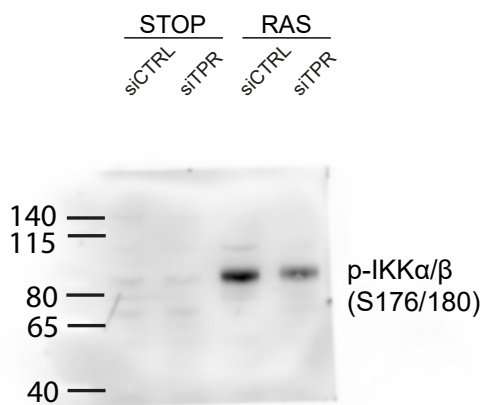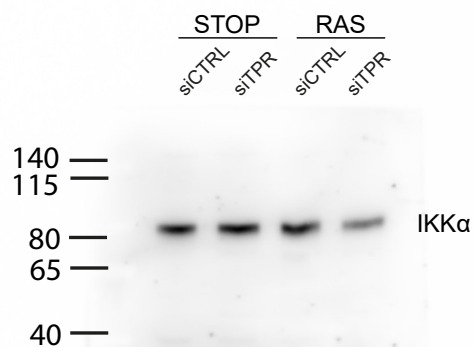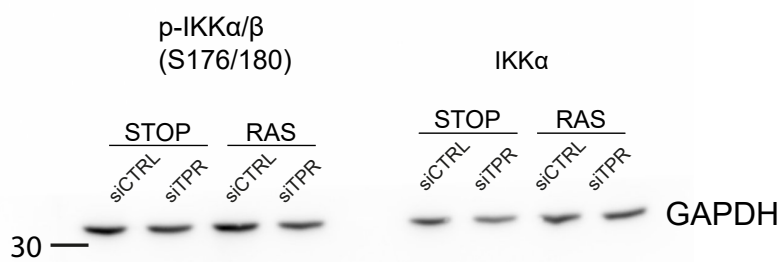

Supplement: Figure 2—figure supplement 1—source data 1. [file elife-101702-fig2-figsupp1-data1.zip › Figure 2 - figure supplement 1 IKK blots.pdf]

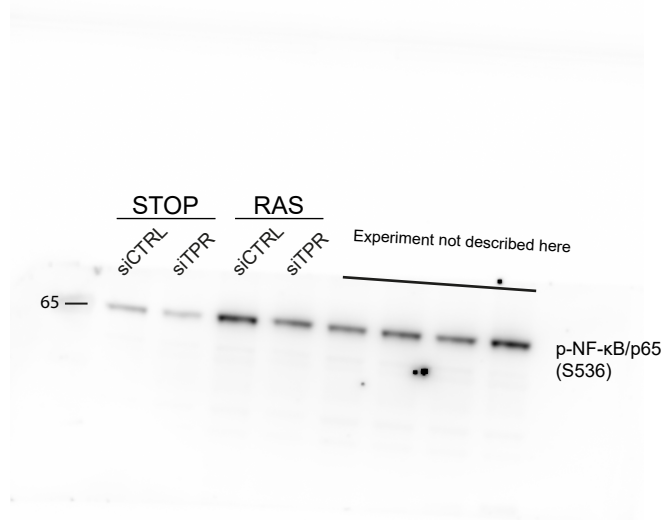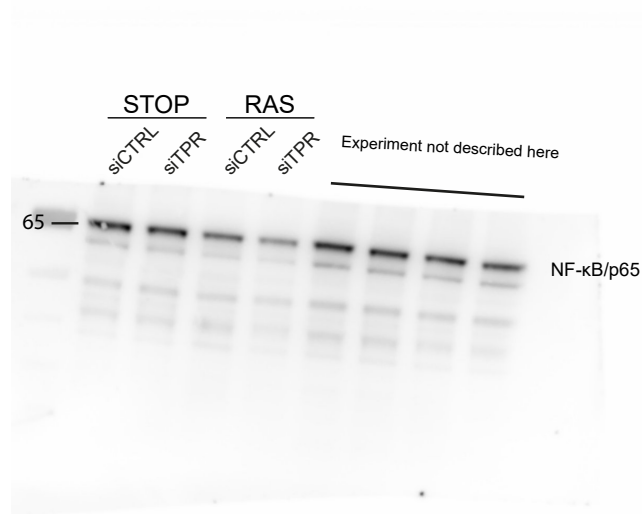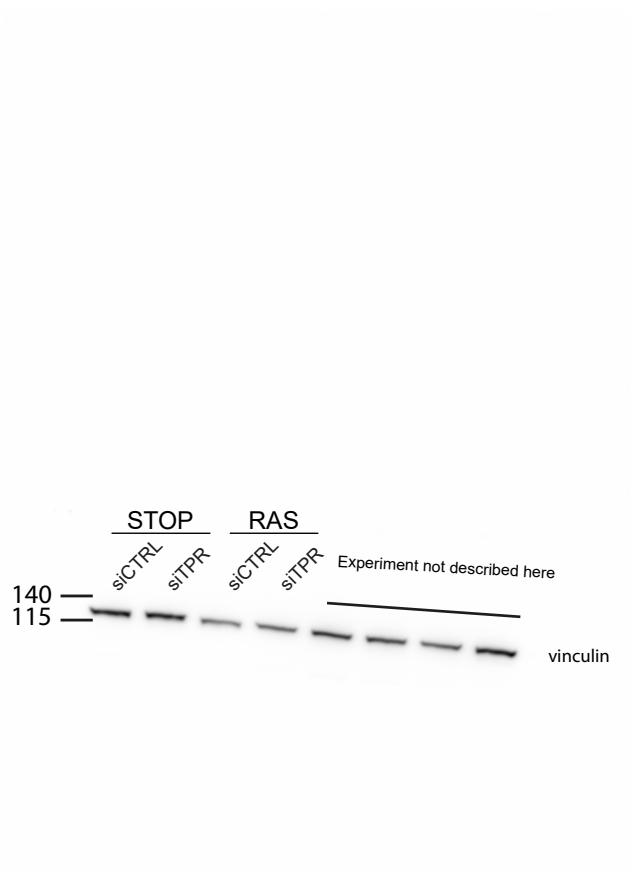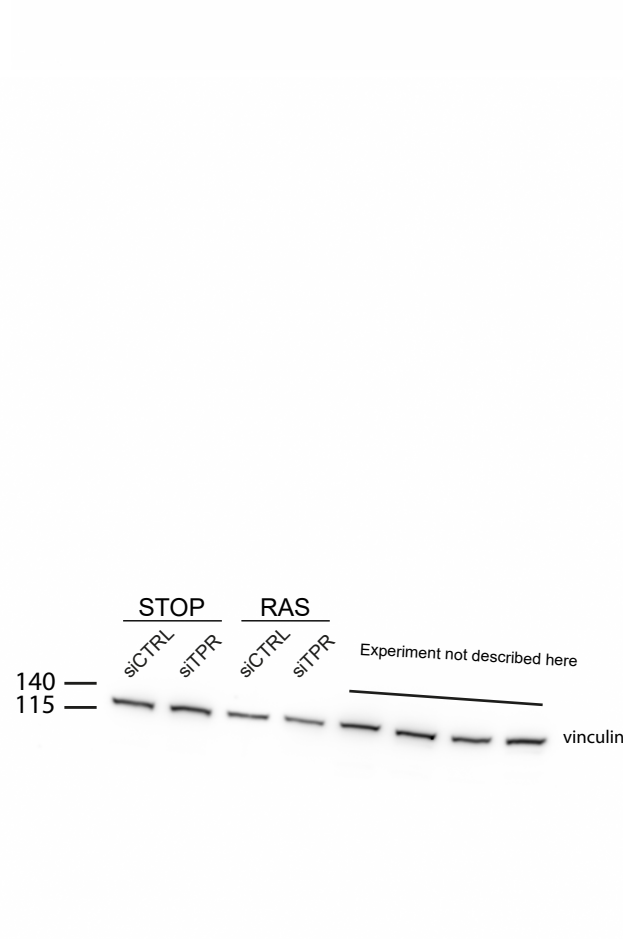

Supplement: Figure 2—figure supplement 1—source data 1. [file elife-101702-fig2-figsupp1-data1.zip › Figure 2 - figure supplement 1 NF-kB blots.pdf]

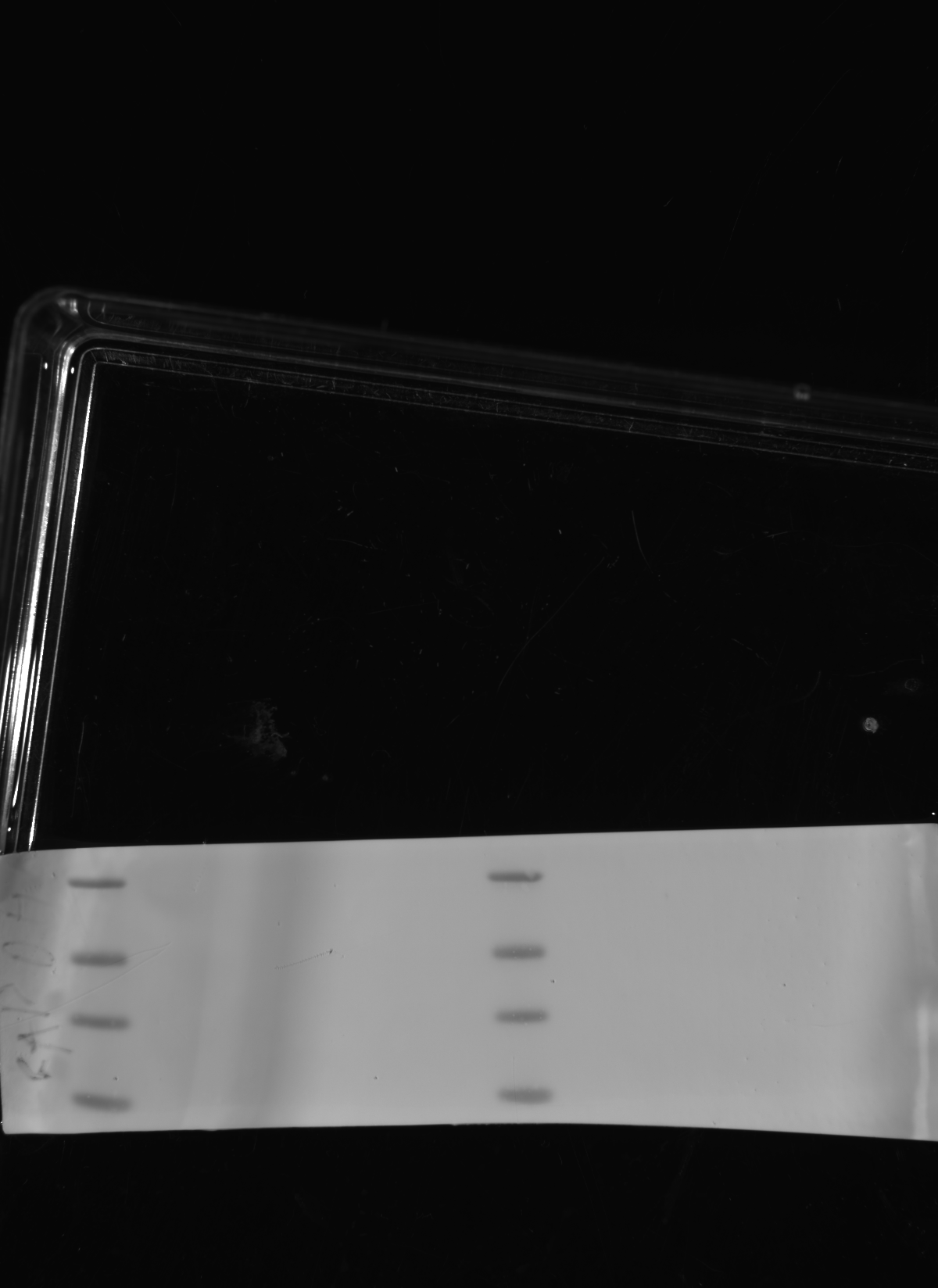

Supplement: Figure 2—figure supplement 1—source data 2. [file elife-101702-fig2-figsupp1-data2.zip › GAPDH for IKK and p-IKK WL.tif]

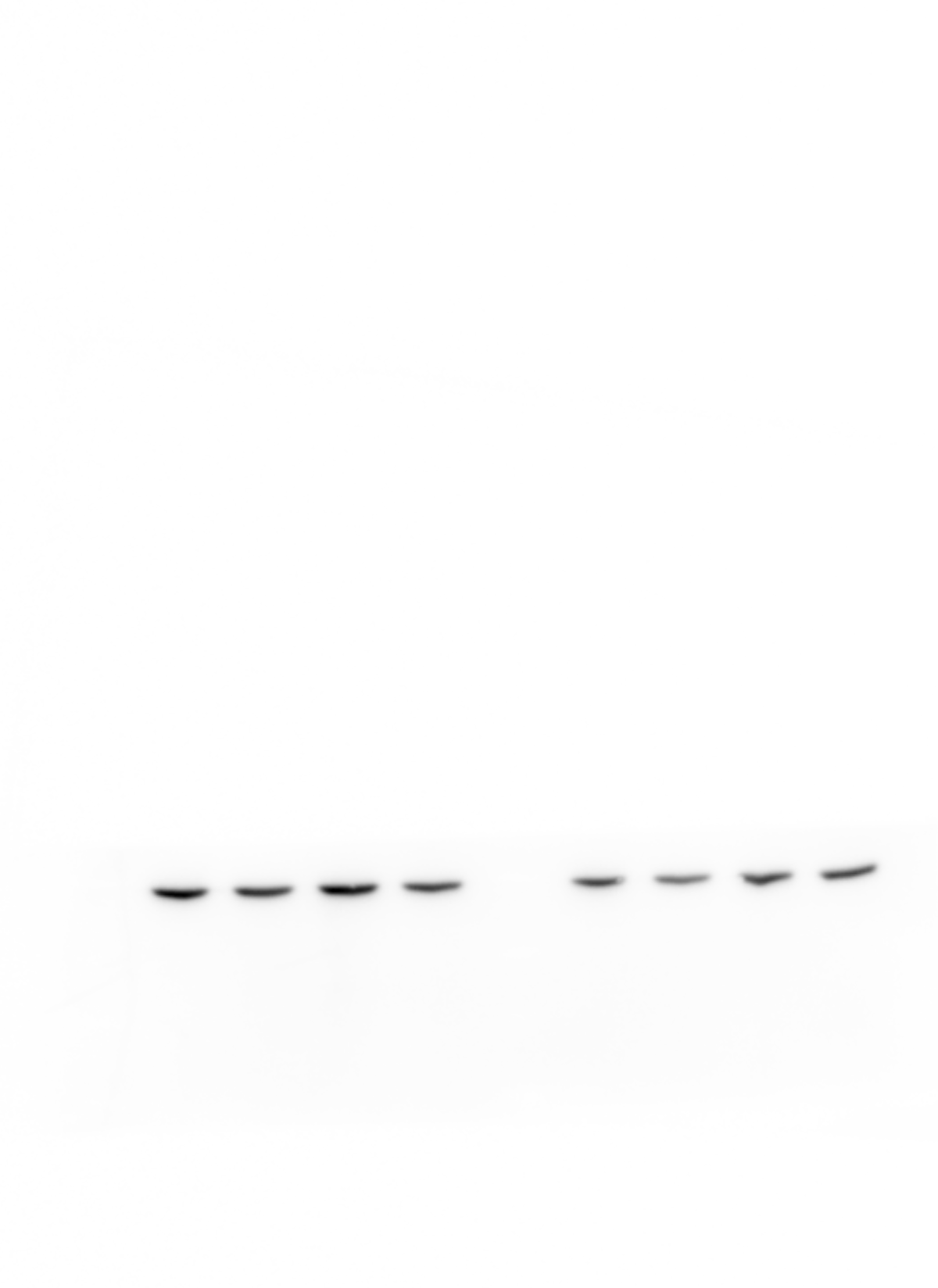

Supplement: Figure 2—figure supplement 1—source data 2. [file elife-101702-fig2-figsupp1-data2.zip › GAPDH for IKK and p-IKK.tif]

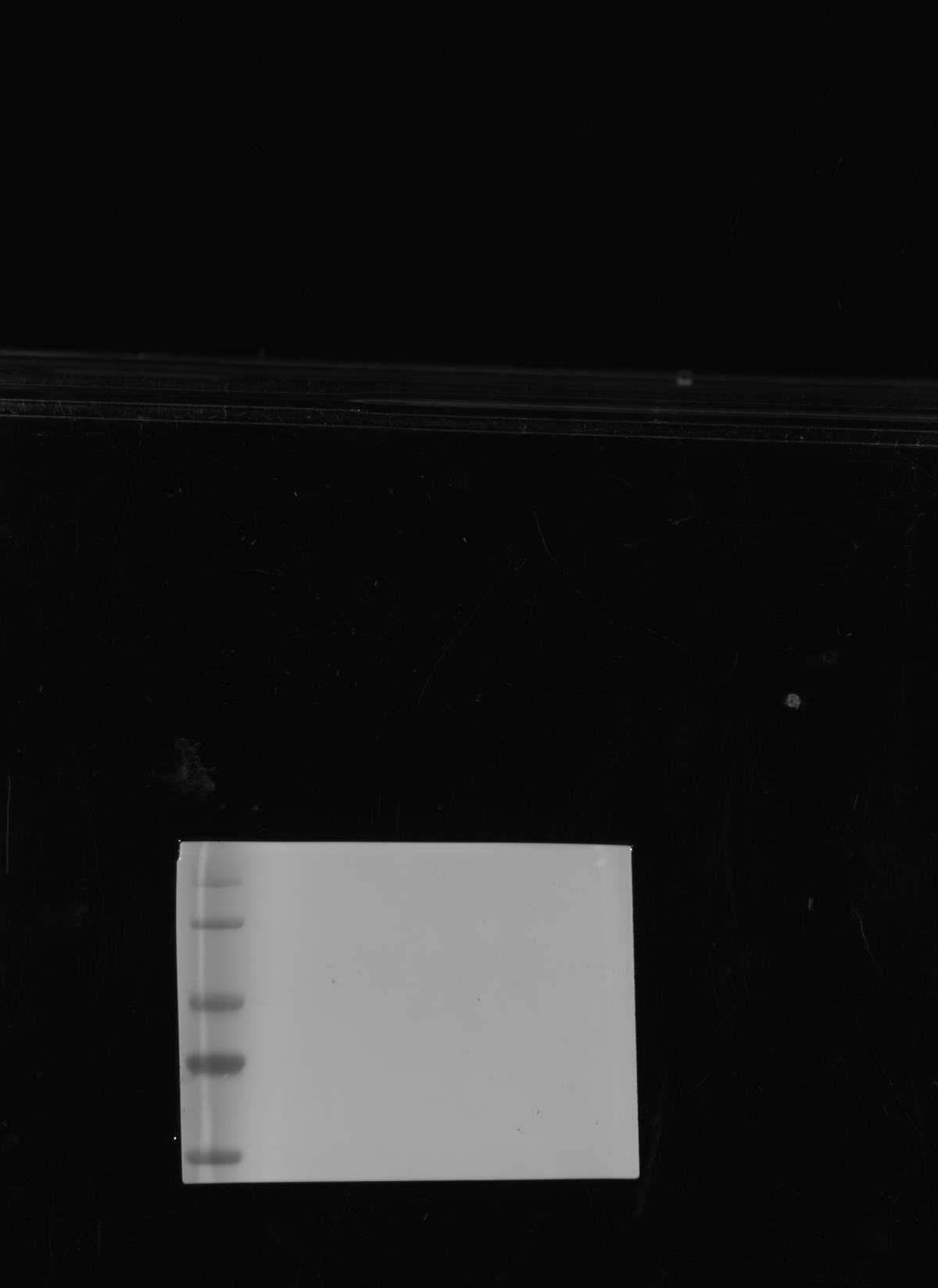

Supplement: Figure 2—figure supplement 1—source data 2. [file elife-101702-fig2-figsupp1-data2.zip › IKK WL.tif]

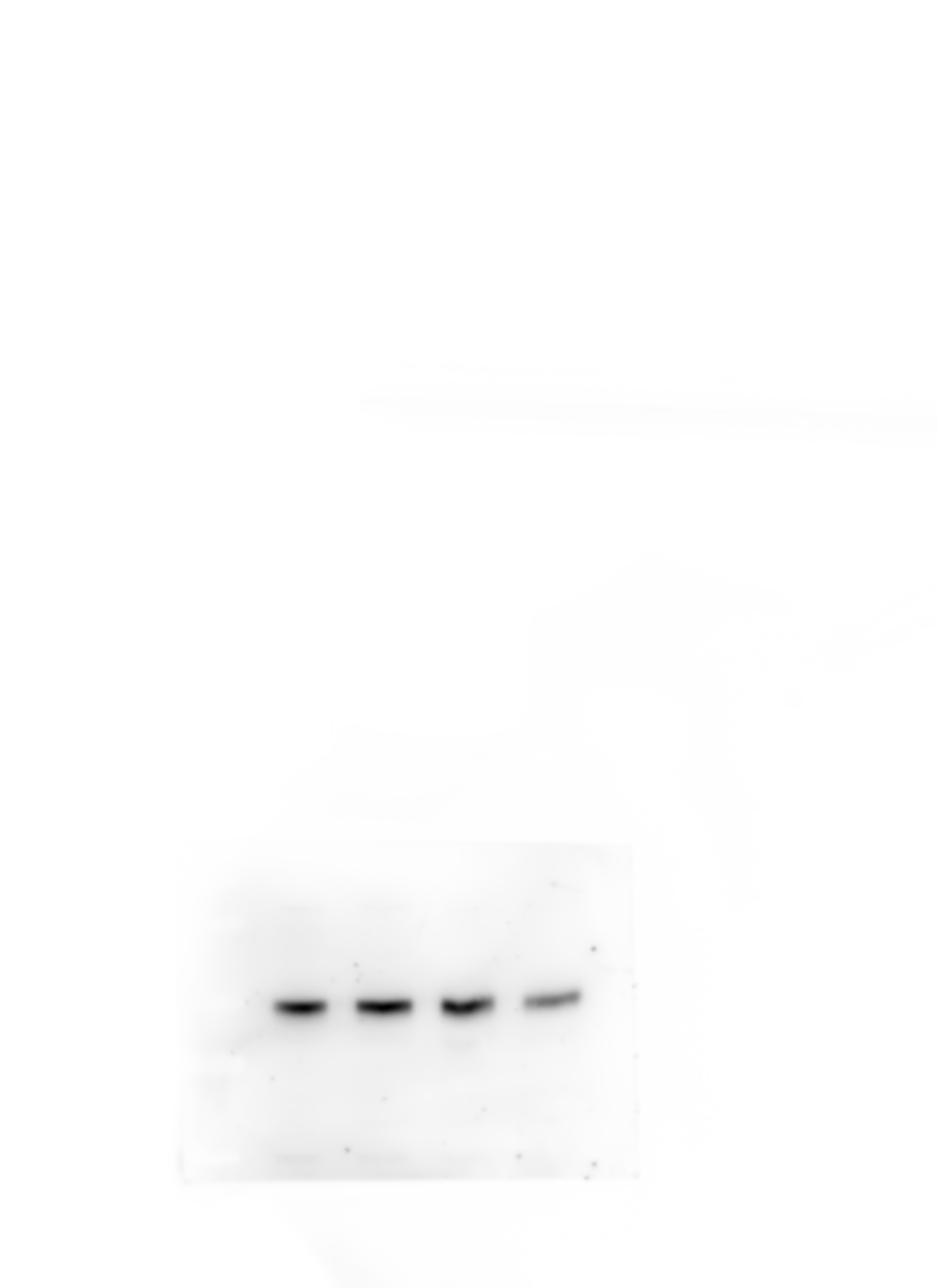

Supplement: Figure 2—figure supplement 1—source data 2. [file elife-101702-fig2-figsupp1-data2.zip › IKK.tif]

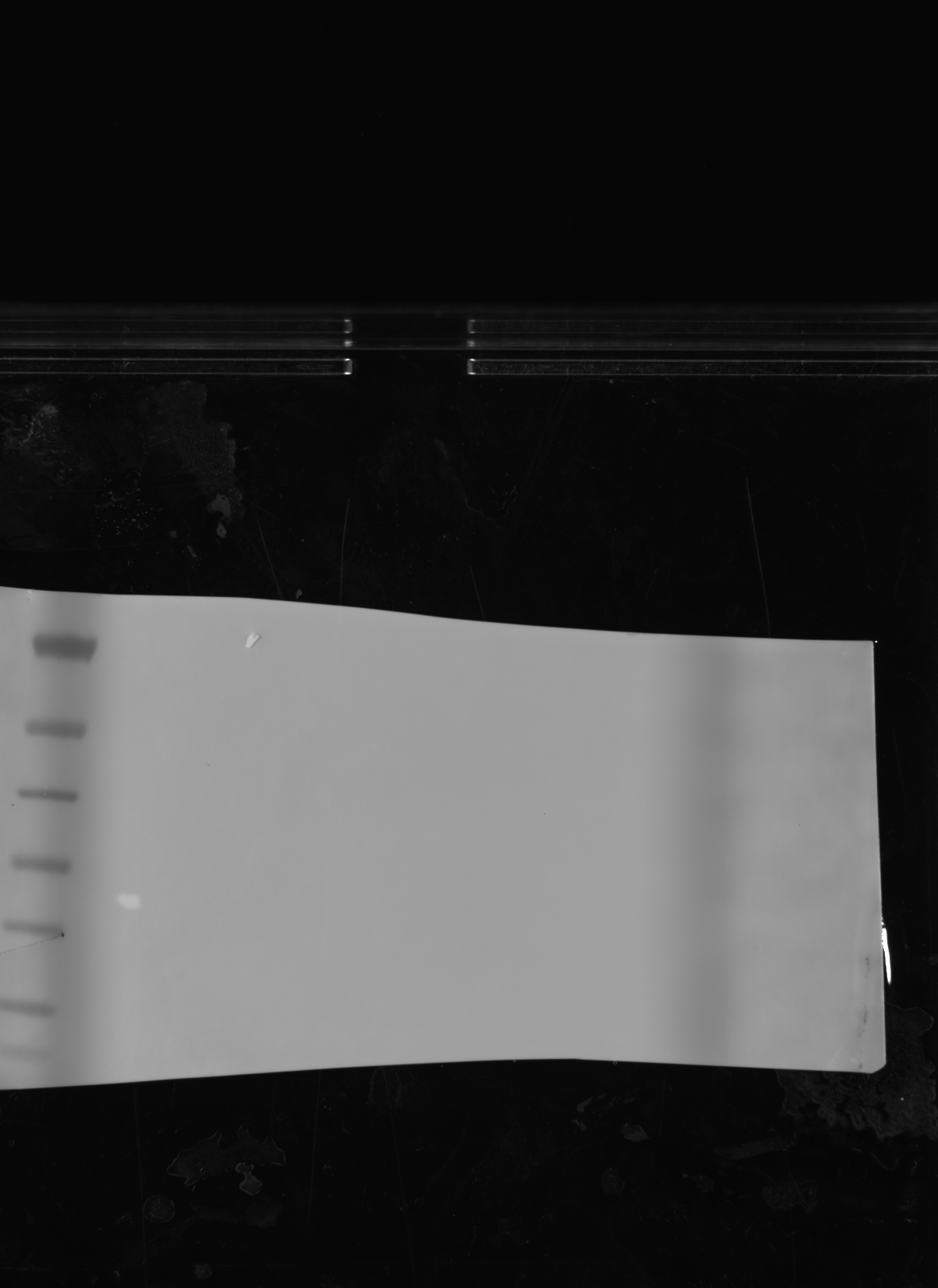

Supplement: Figure 2—figure supplement 1—source data 2. [file elife-101702-fig2-figsupp1-data2.zip › NF-kB WL.tif]

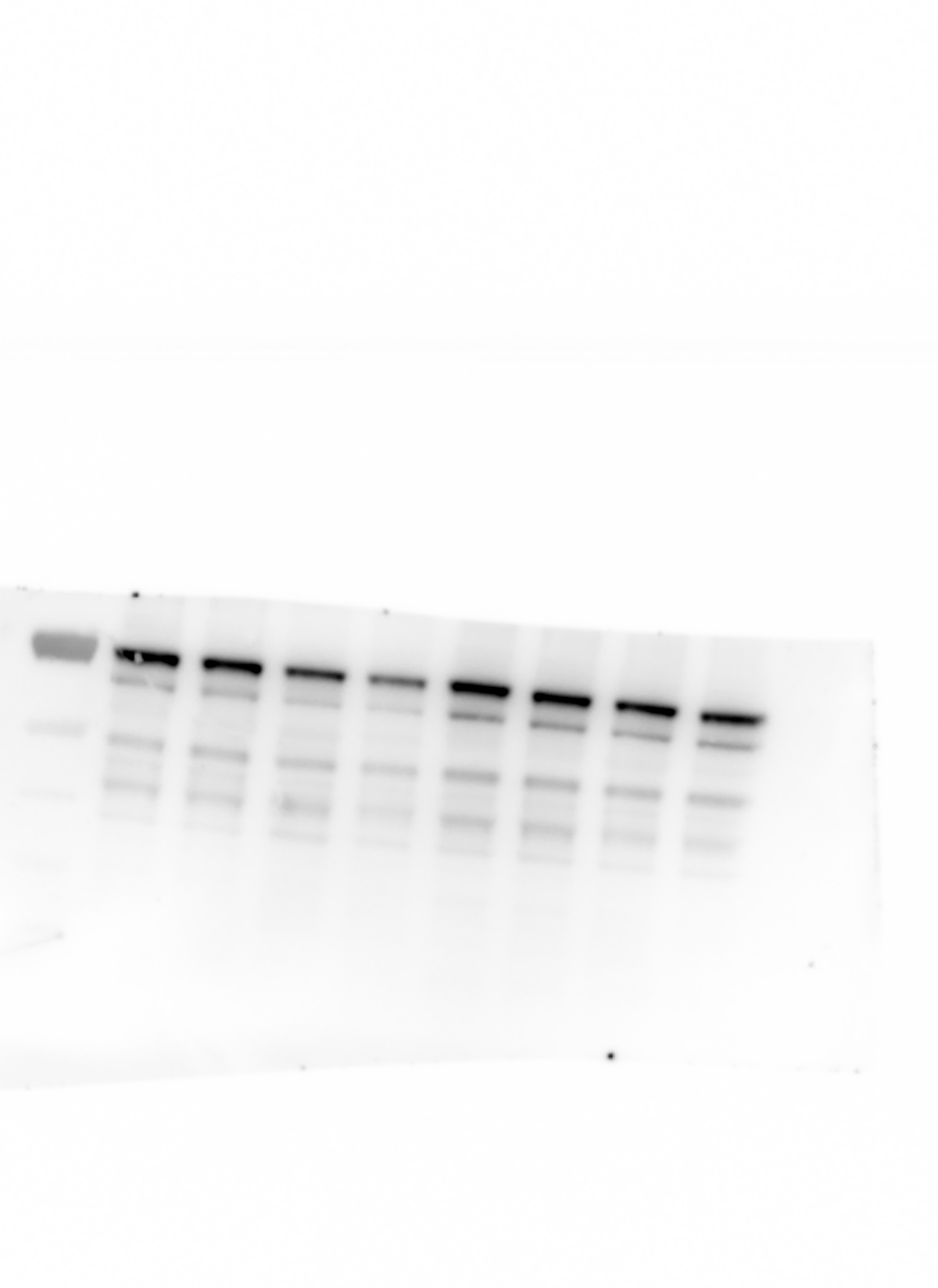

Supplement: Figure 2—figure supplement 1—source data 2. [file elife-101702-fig2-figsupp1-data2.zip › NF-kB.tif]

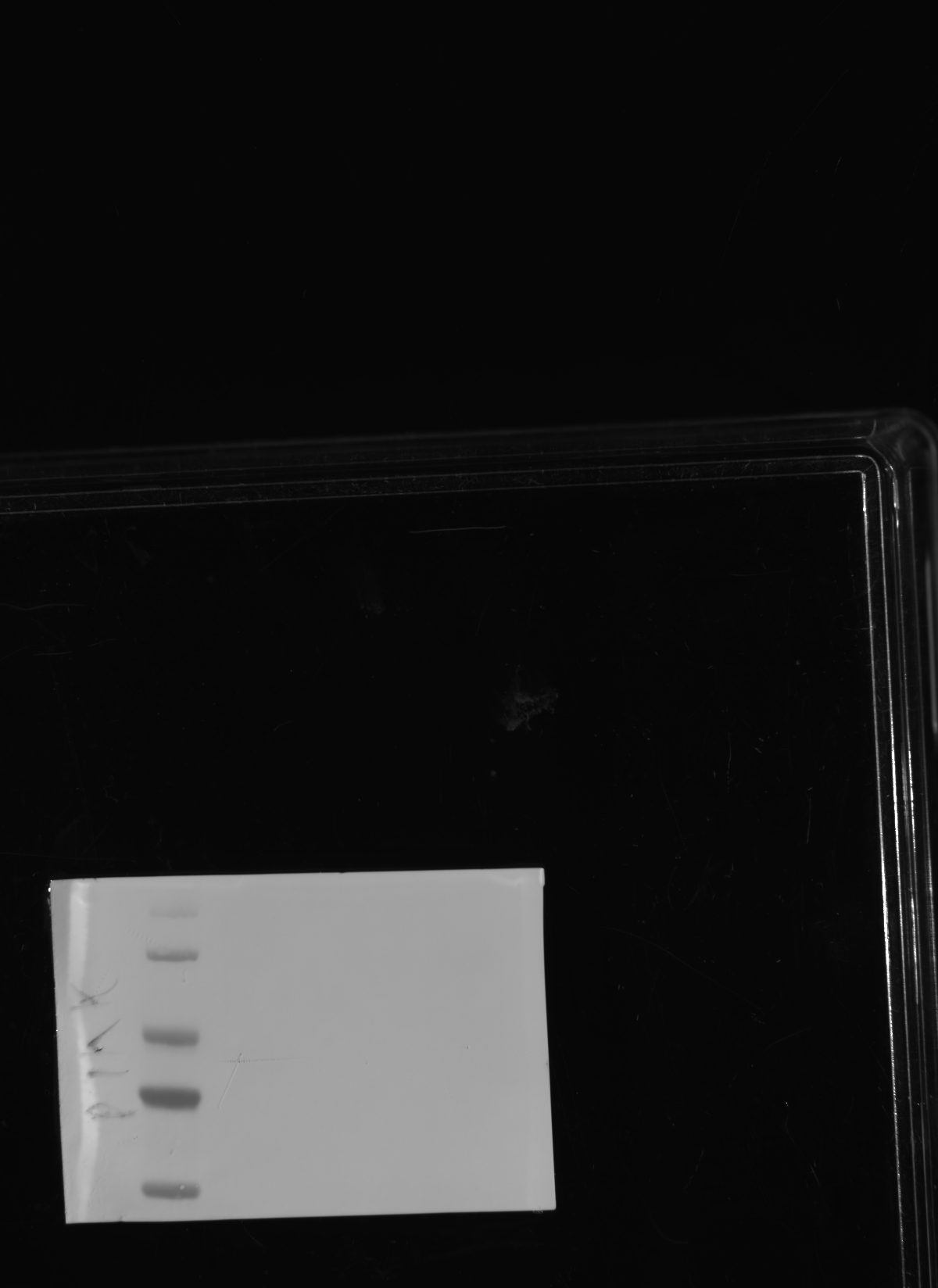

Supplement: Figure 2—figure supplement 1—source data 2. [file elife-101702-fig2-figsupp1-data2.zip › p-IKK WL.tif]

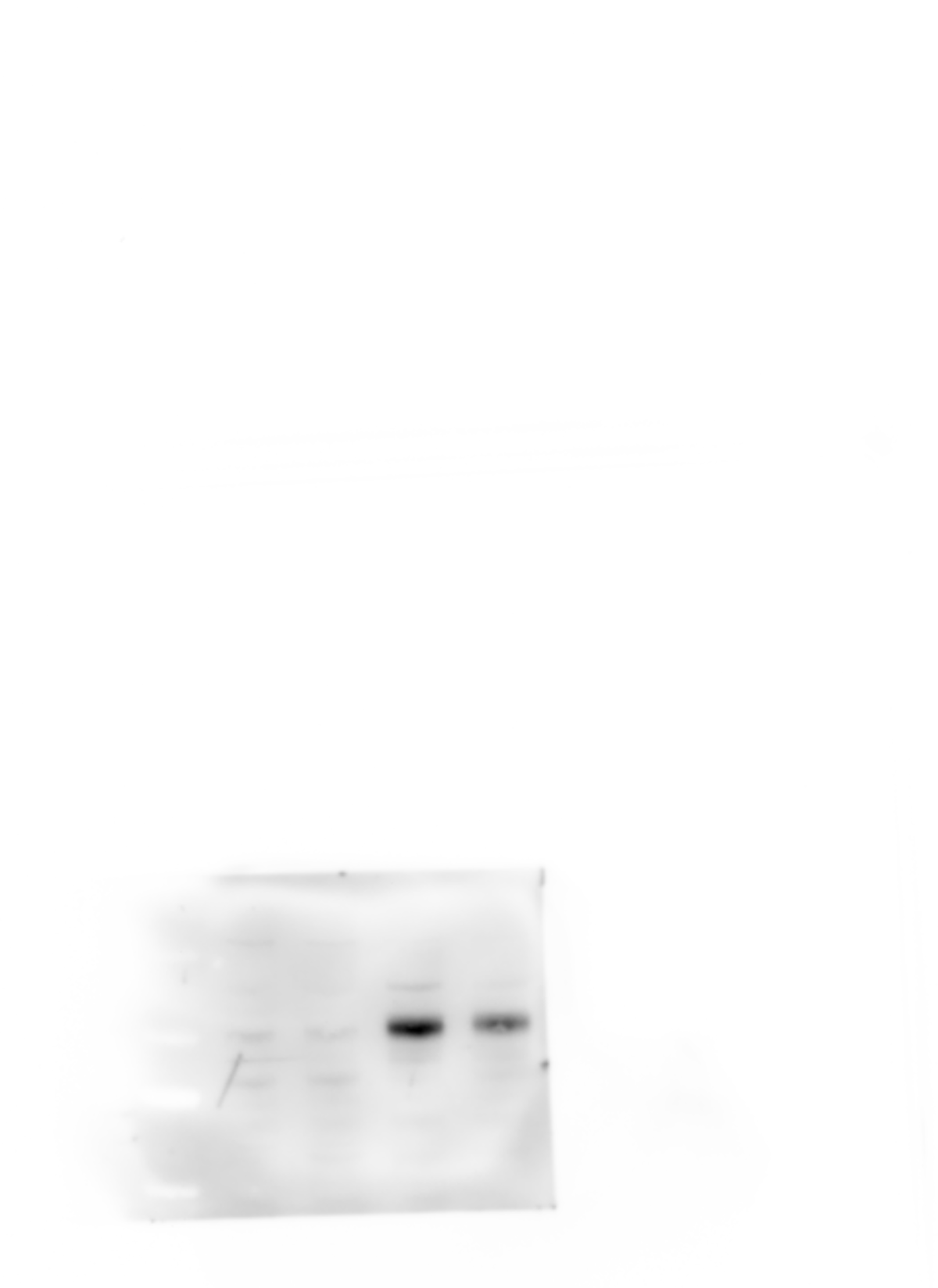

Supplement: Figure 2—figure supplement 1—source data 2. [file elife-101702-fig2-figsupp1-data2.zip › p-IKK.tif]

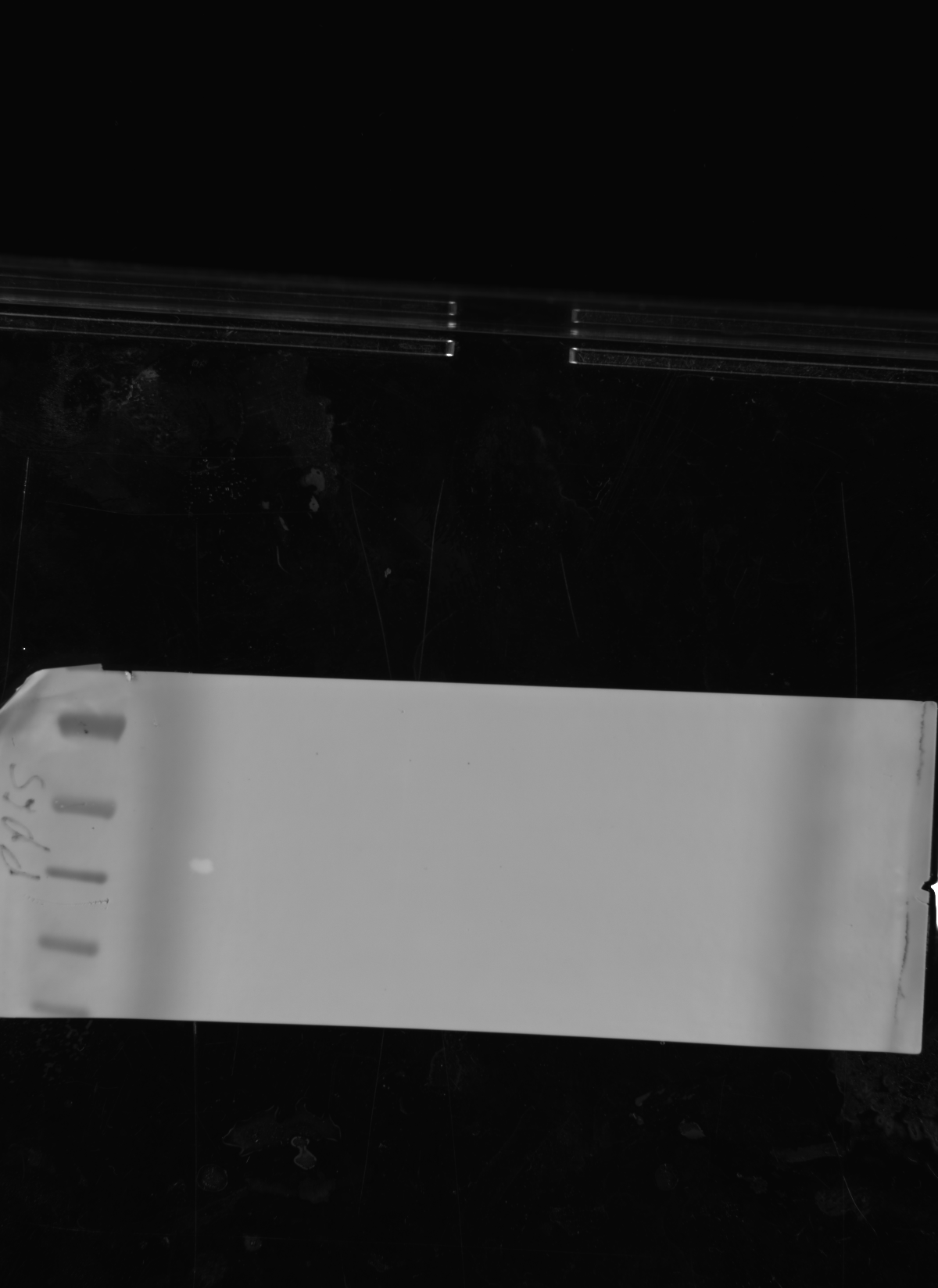

Supplement: Figure 2—figure supplement 1—source data 2. [file elife-101702-fig2-figsupp1-data2.zip › p-NF-kB WL.tif]

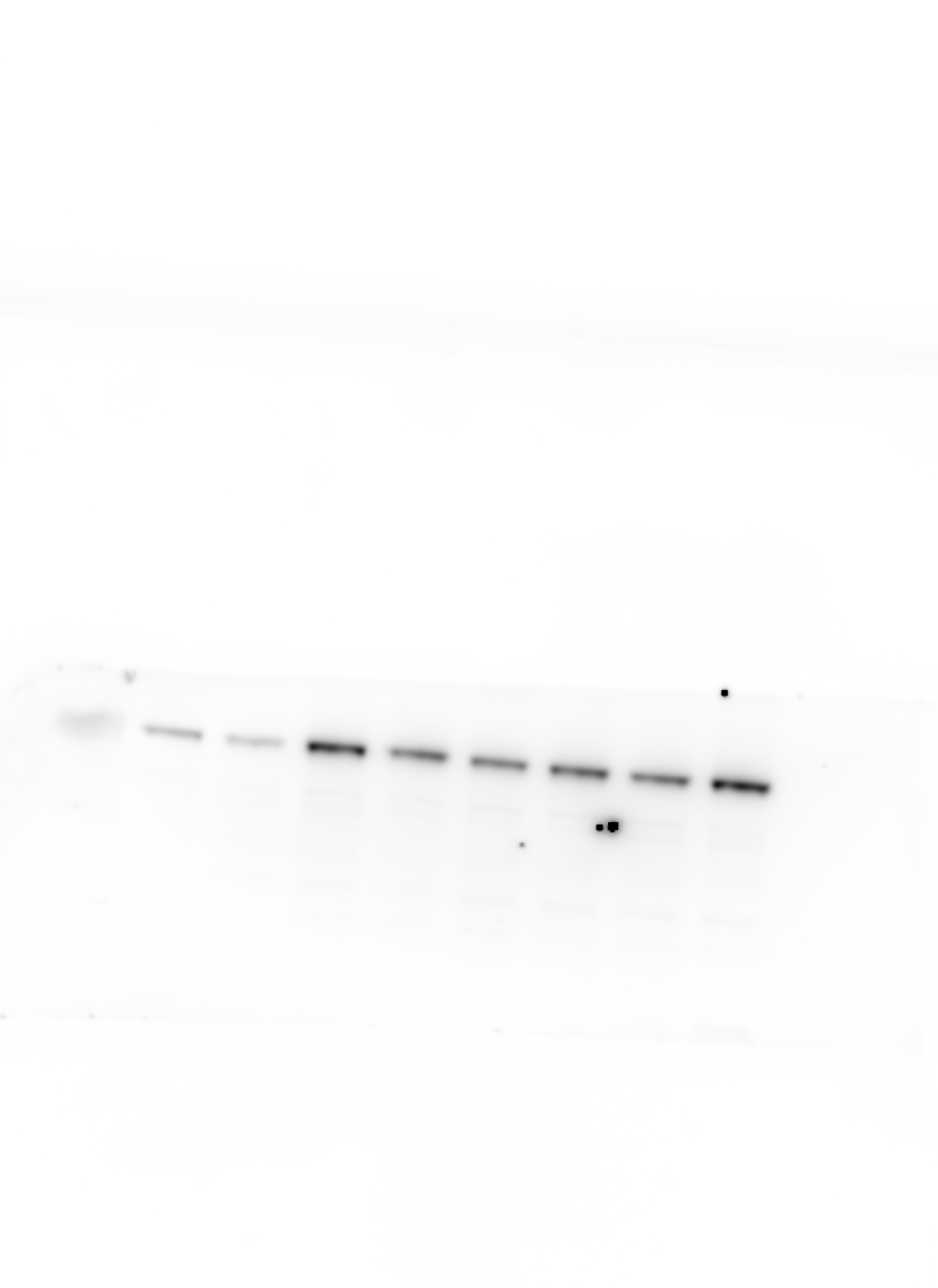

Supplement: Figure 2—figure supplement 1—source data 2. [file elife-101702-fig2-figsupp1-data2.zip › p-NF-kB.tif]

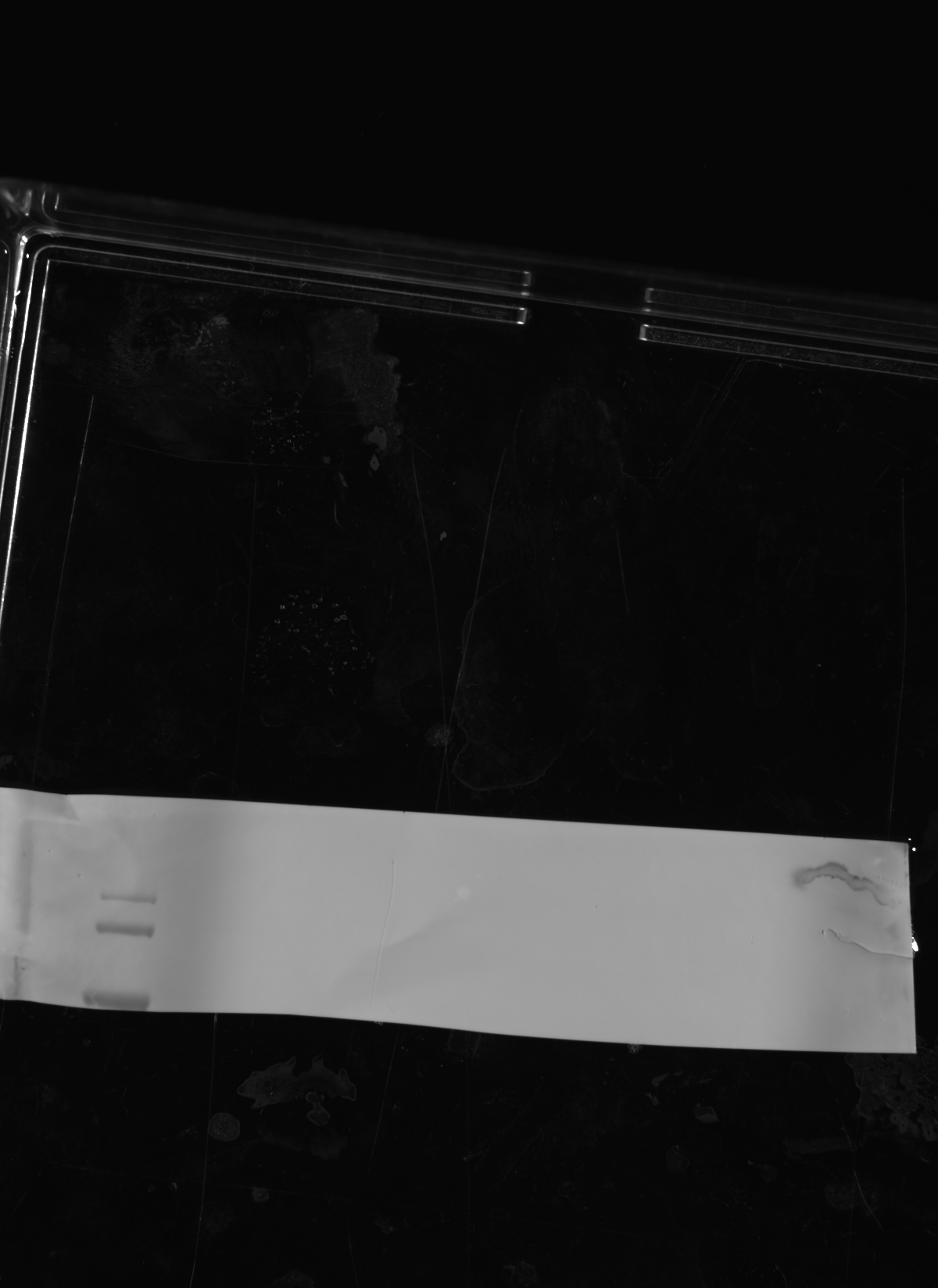

Supplement: Figure 2—figure supplement 1—source data 2. [file elife-101702-fig2-figsupp1-data2.zip › Vinculin for NF-kB WL.tif]

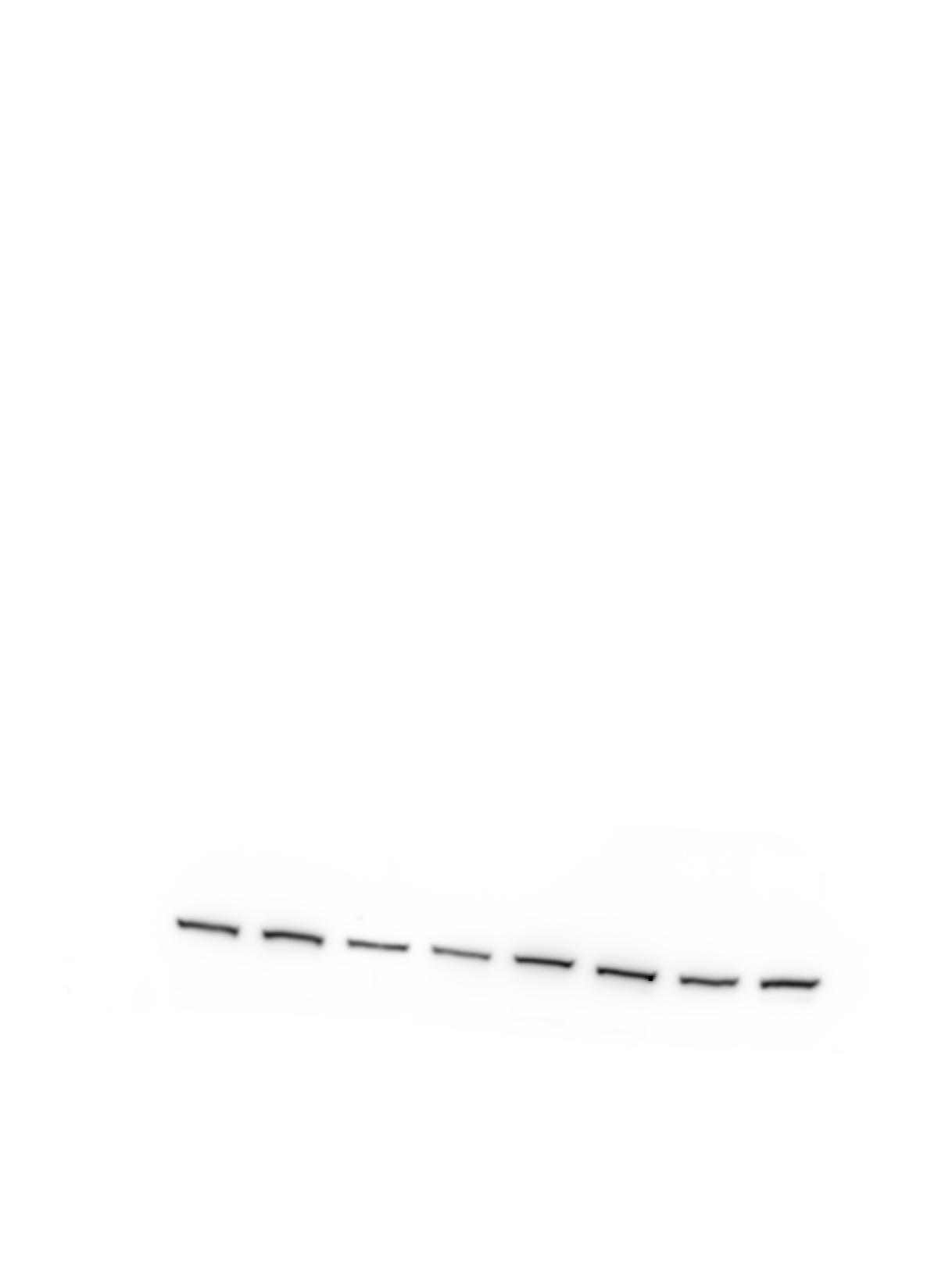

Supplement: Figure 2—figure supplement 1—source data 2. [file elife-101702-fig2-figsupp1-data2.zip › Vinculin for NF-kB.tif]

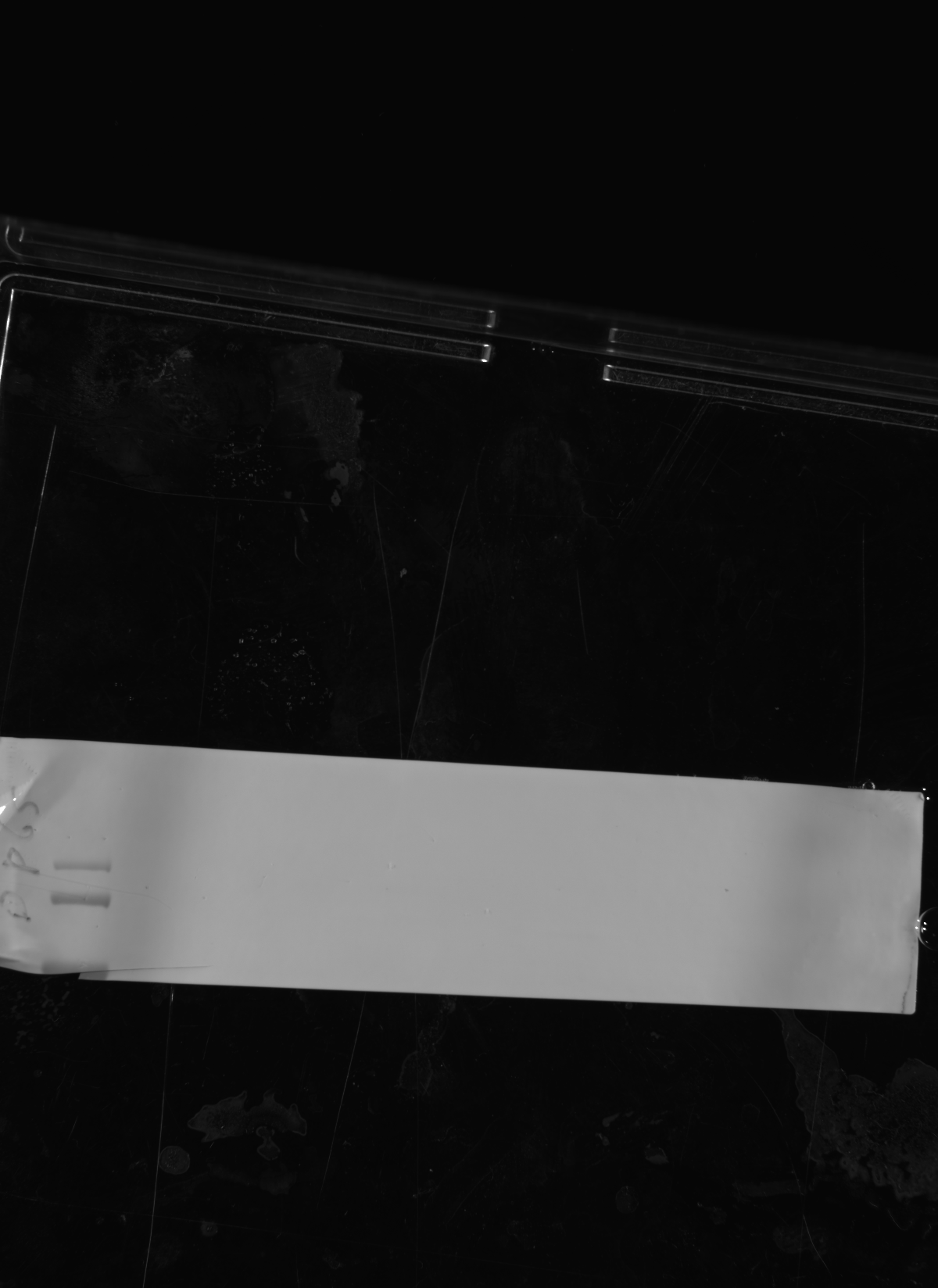

Supplement: Figure 2—figure supplement 1—source data 2. [file elife-101702-fig2-figsupp1-data2.zip › Vinculin for p-NF-kB WL.tif]

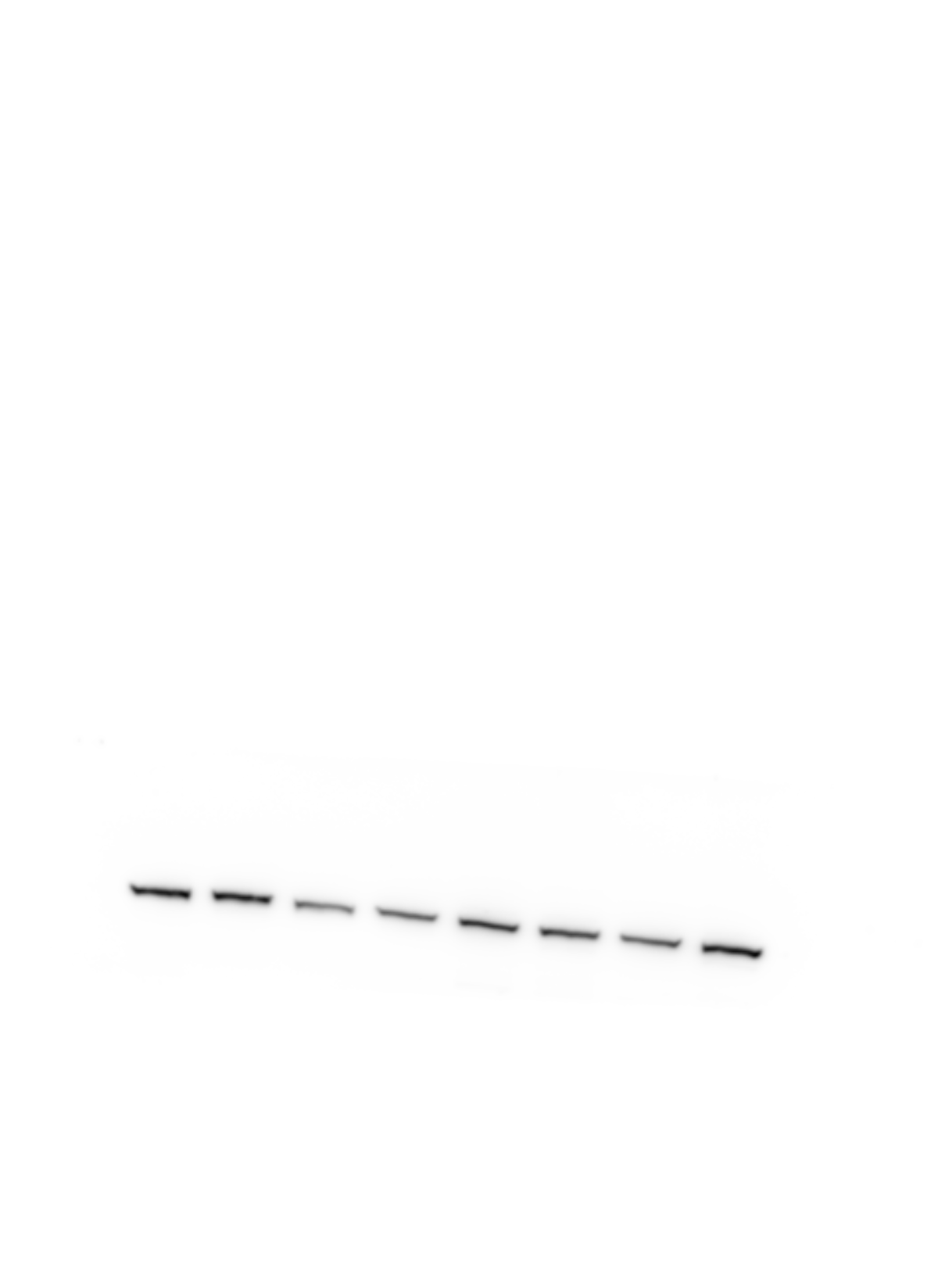

Supplement: Figure 2—figure supplement 1—source data 2. [file elife-101702-fig2-figsupp1-data2.zip › Vinculin for p-NF-kB.tif]

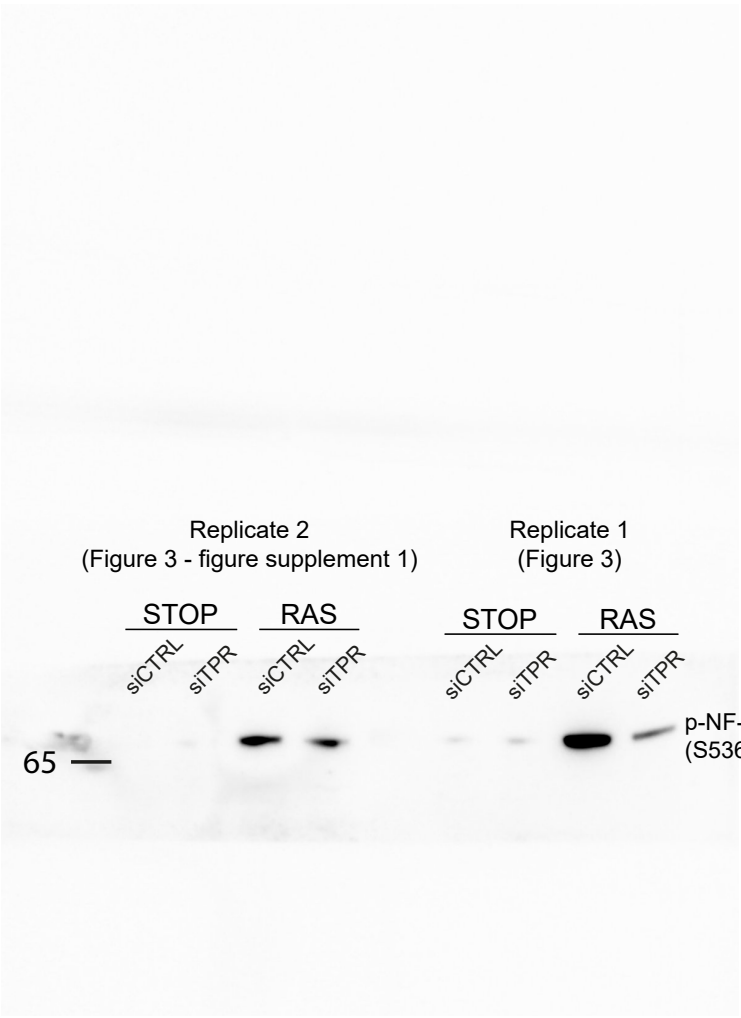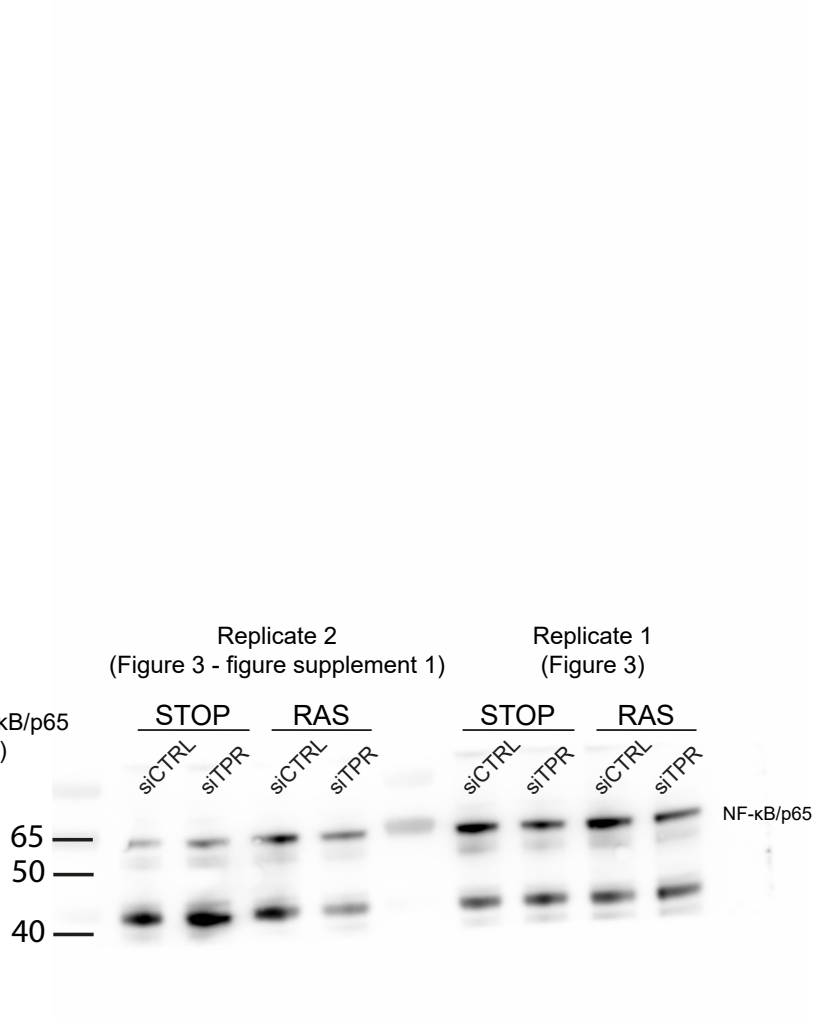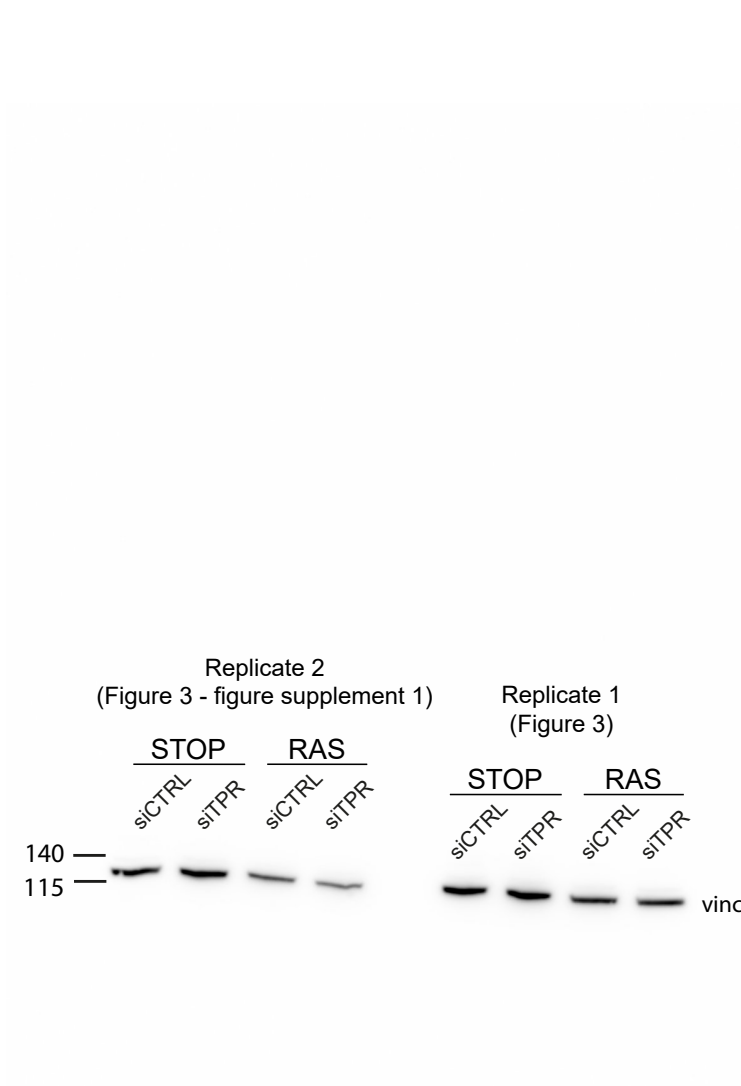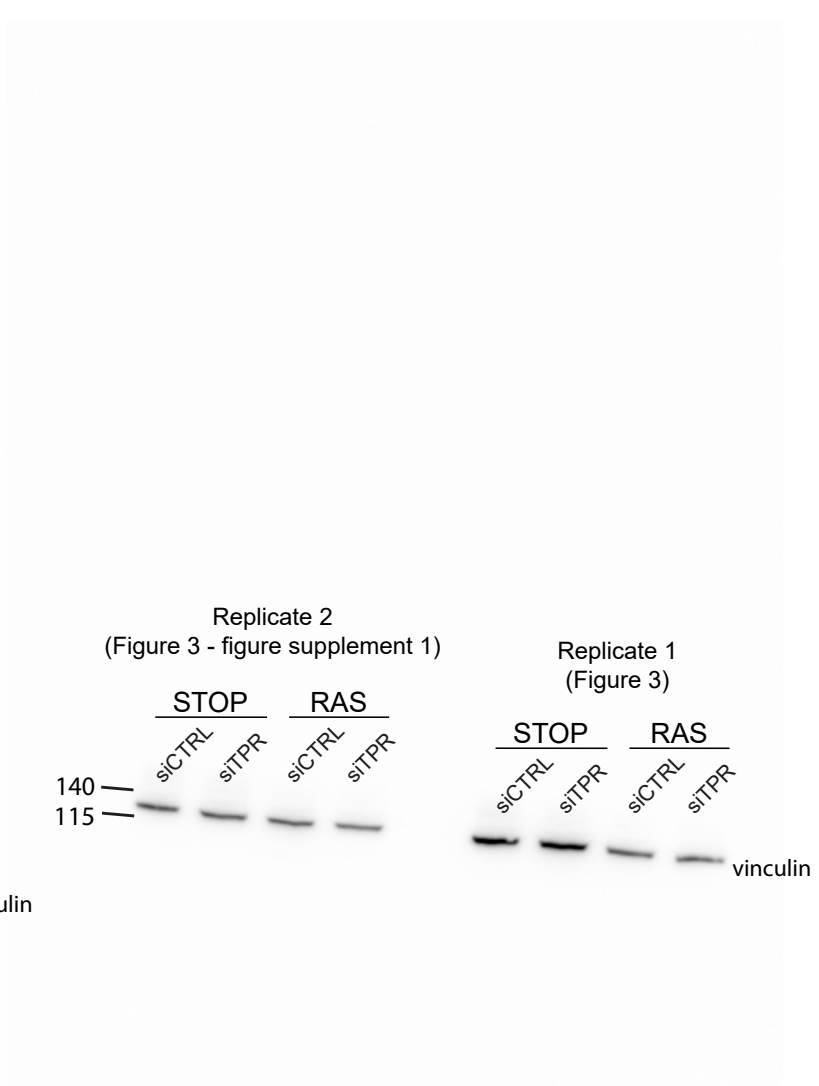

Supplement: Figure 3—source data 2. [file elife-101702-fig3-data2.zip › Figure 3 Uncropped NF-kB day 5 blots.pdf]

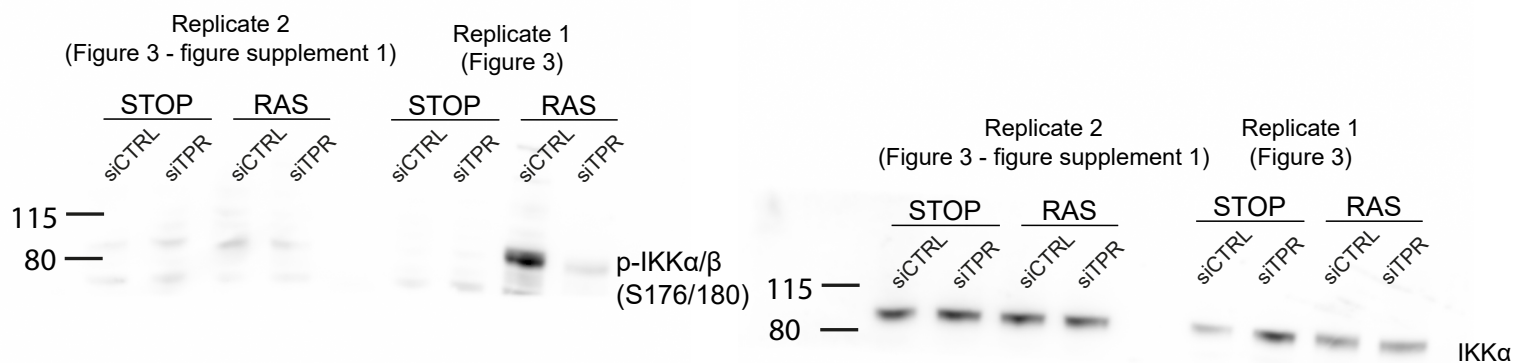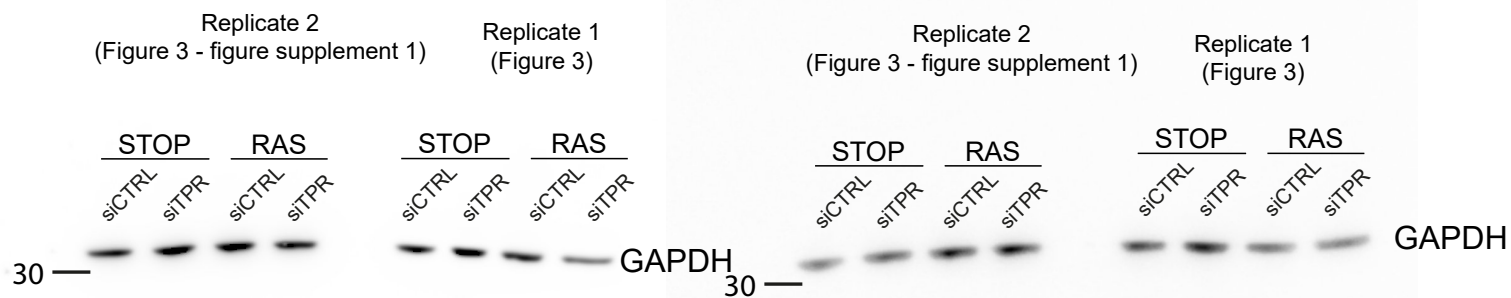

Supplement: Figure 3—source data 2. [file elife-101702-fig3-data2.zip › Figure 3 uncropped IKK day 3.pdf]

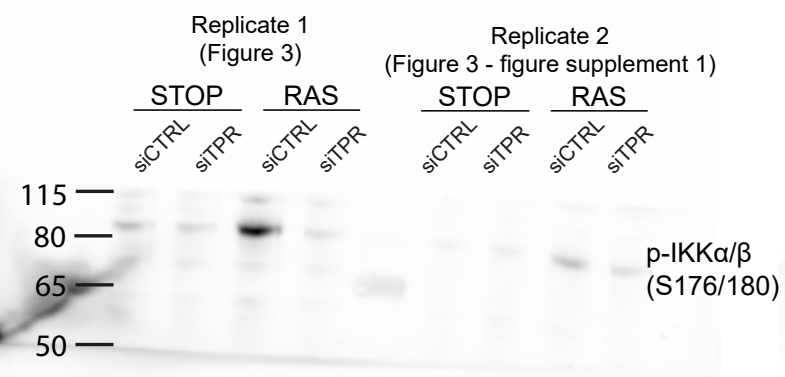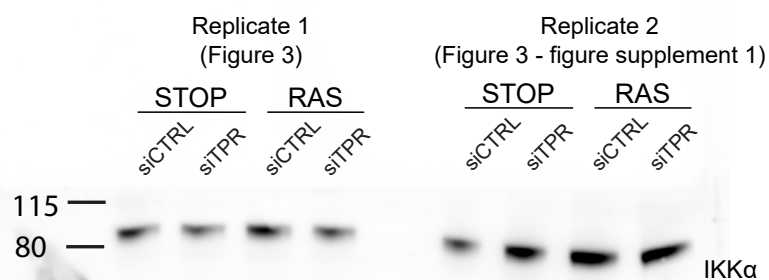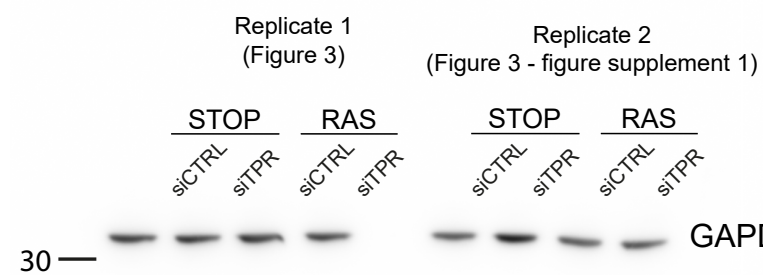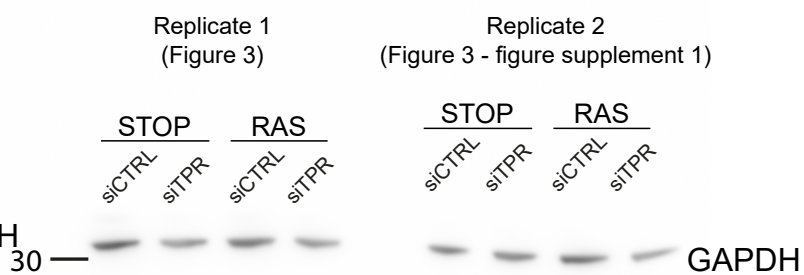

Supplement: Figure 3—source data 2. [file elife-101702-fig3-data2.zip › Figure 3 uncropped IKK day 5.pdf]

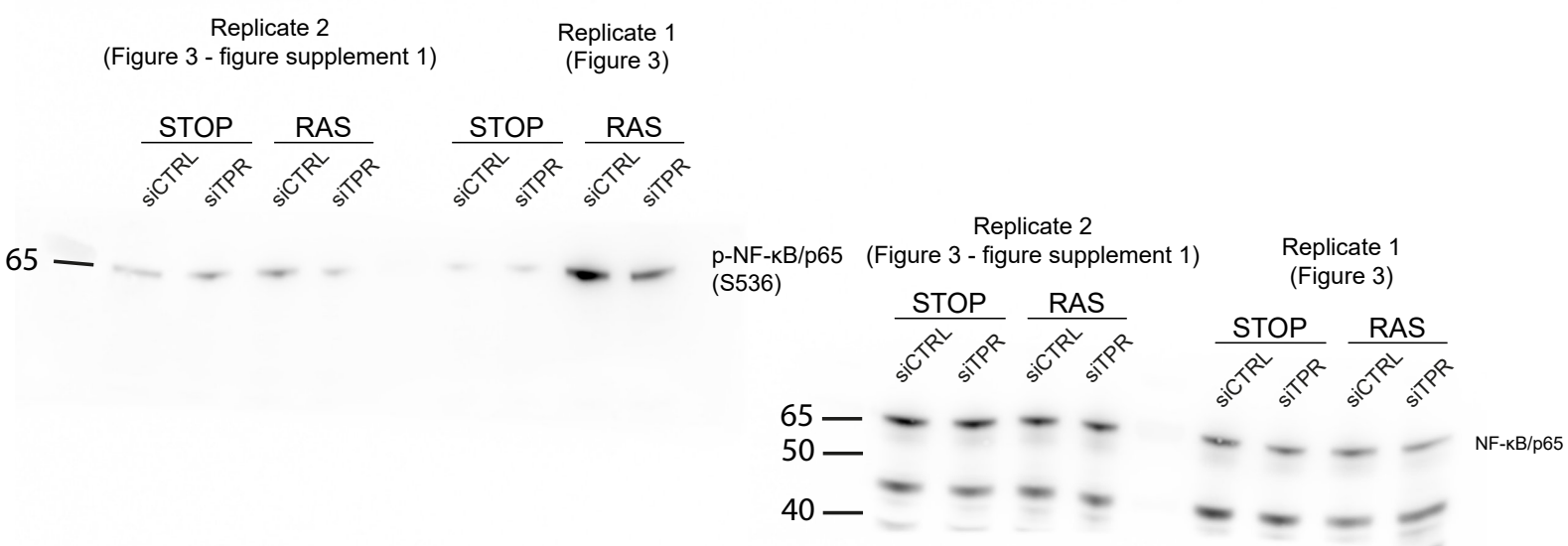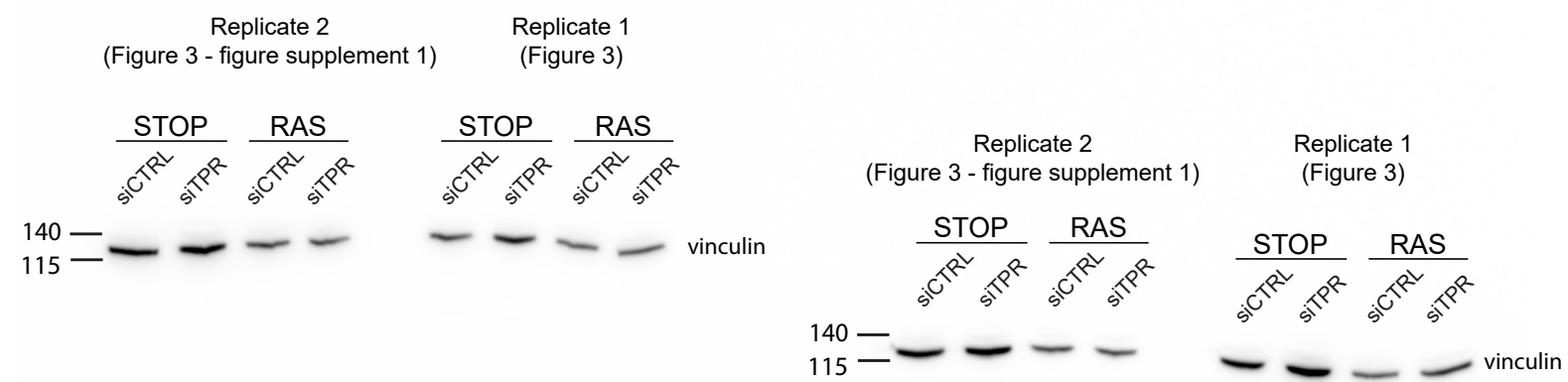

Supplement: Figure 3—source data 2. [file elife-101702-fig3-data2.zip › Figure 3 Uncropped NF-kB day 3 blots.pdf]

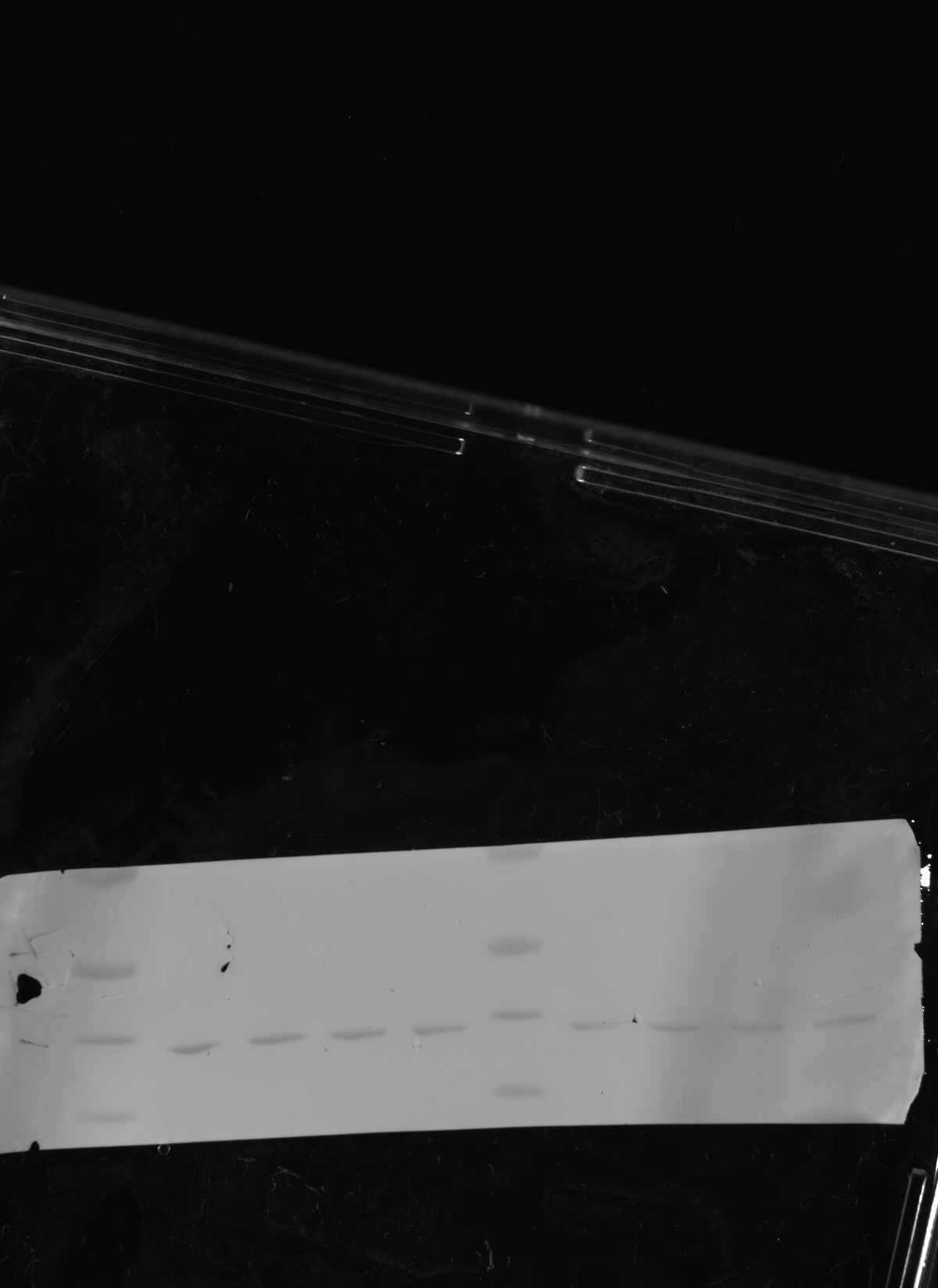

Supplement: Figure 3—source data 3. [file elife-101702-fig3-data3.zip › GAPDH for IKK day 3 WL.tif]

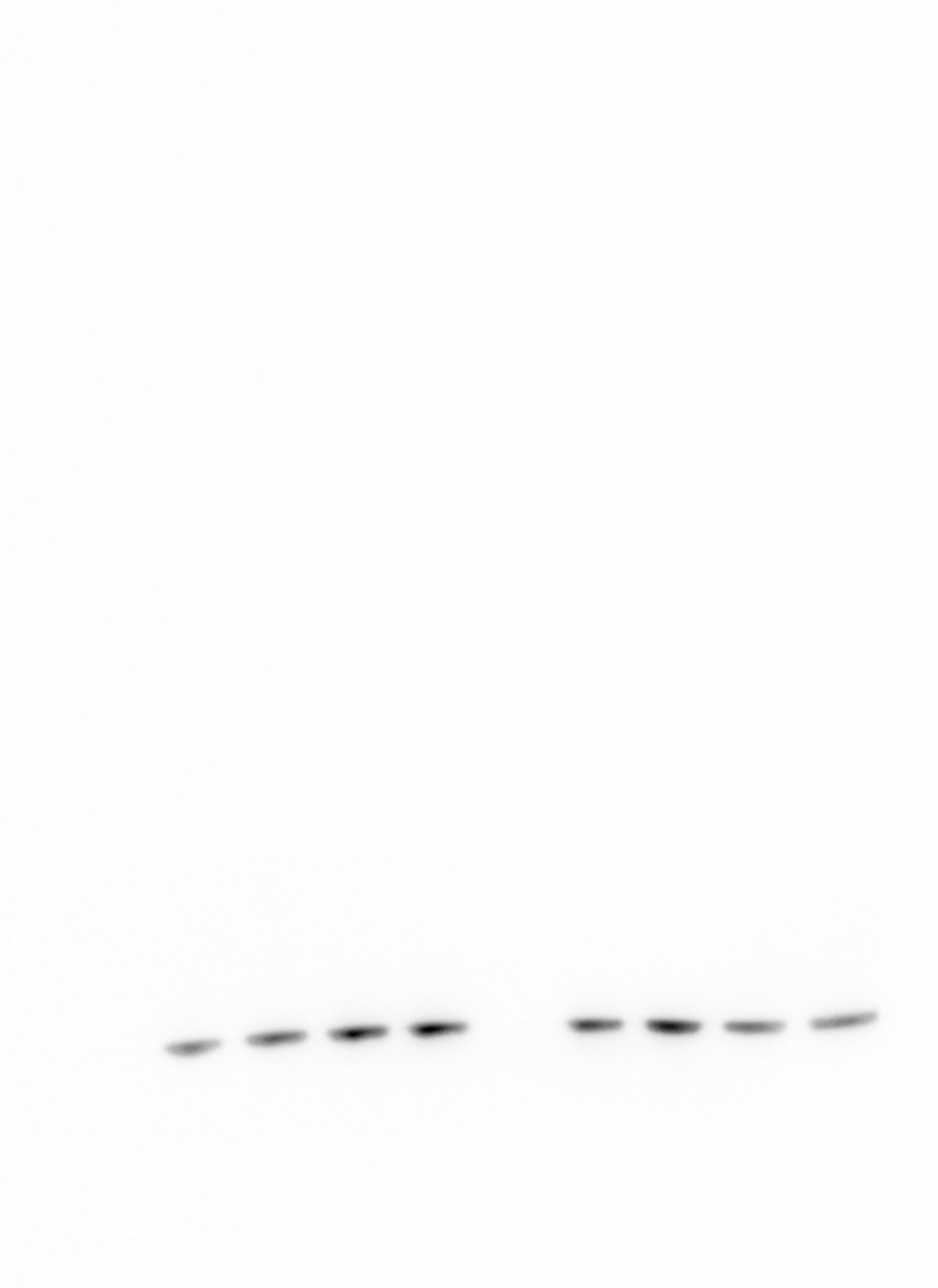

Supplement: Figure 3—source data 3. [file elife-101702-fig3-data3.zip › GAPDH for IKK day 3.tif]

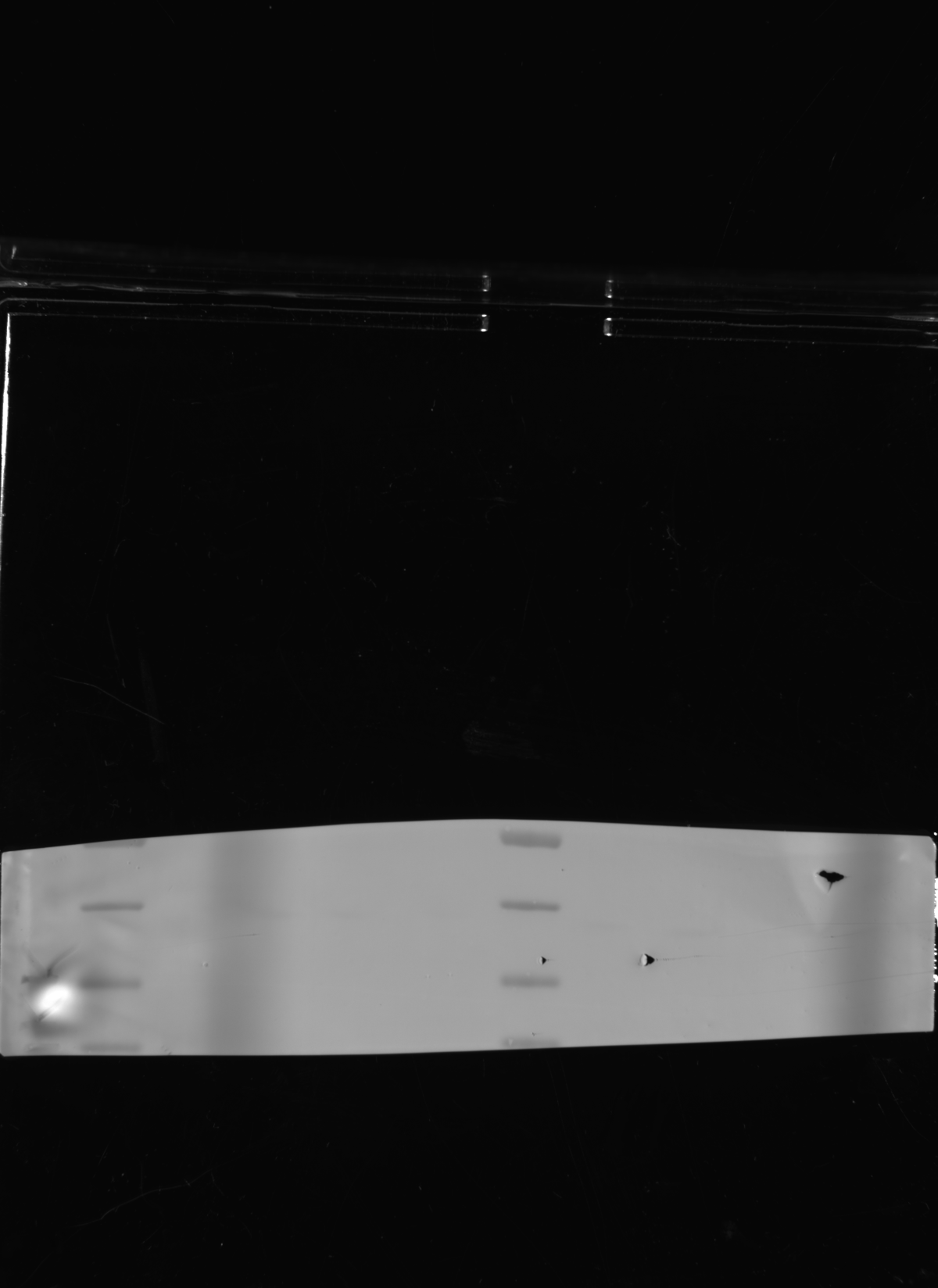

Supplement: Figure 3—source data 3. [file elife-101702-fig3-data3.zip › GAPDH for IKK day 5 WL.tif]

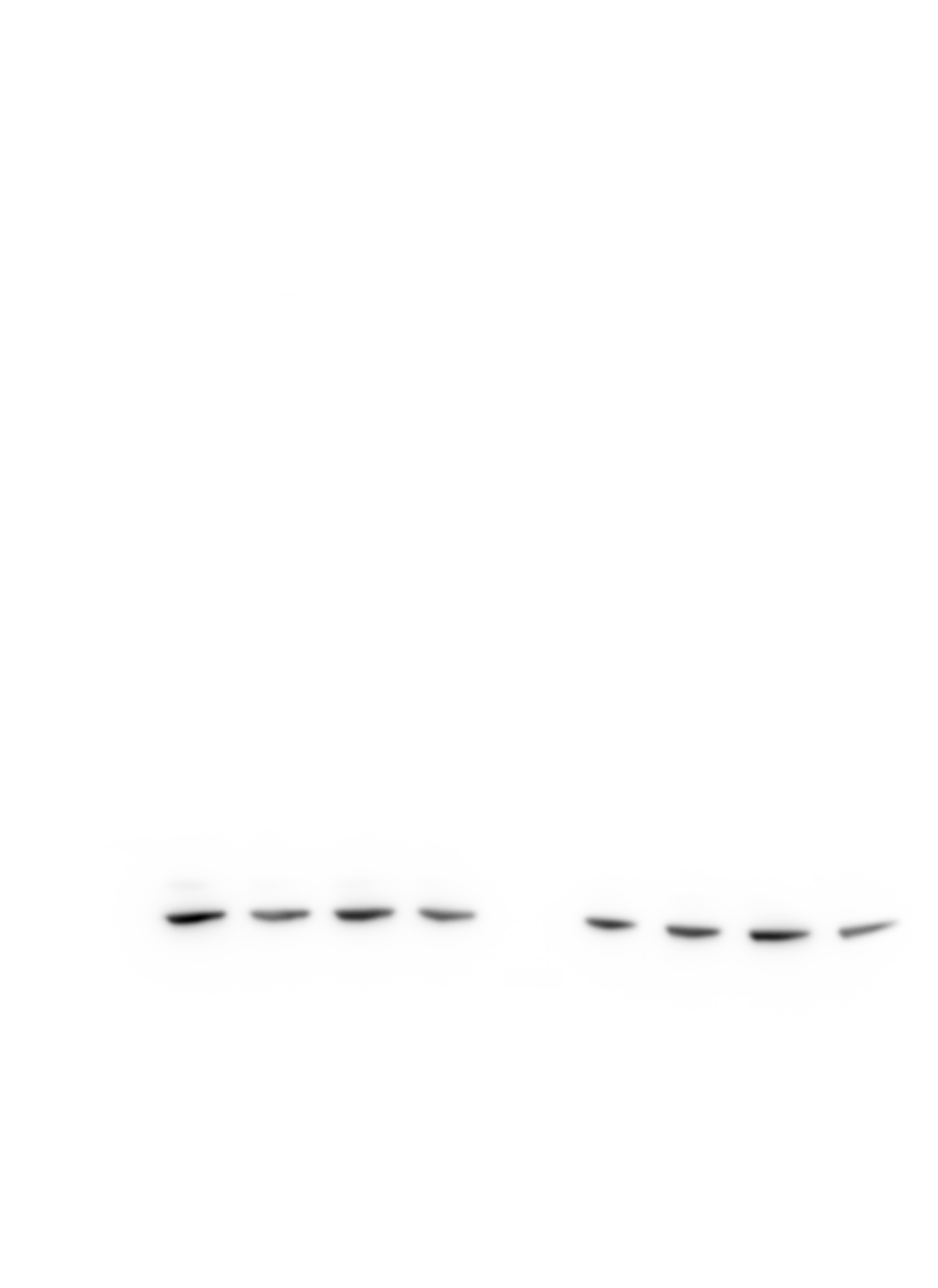

Supplement: Figure 3—source data 3. [file elife-101702-fig3-data3.zip › GAPDH for IKK day 5.tif]

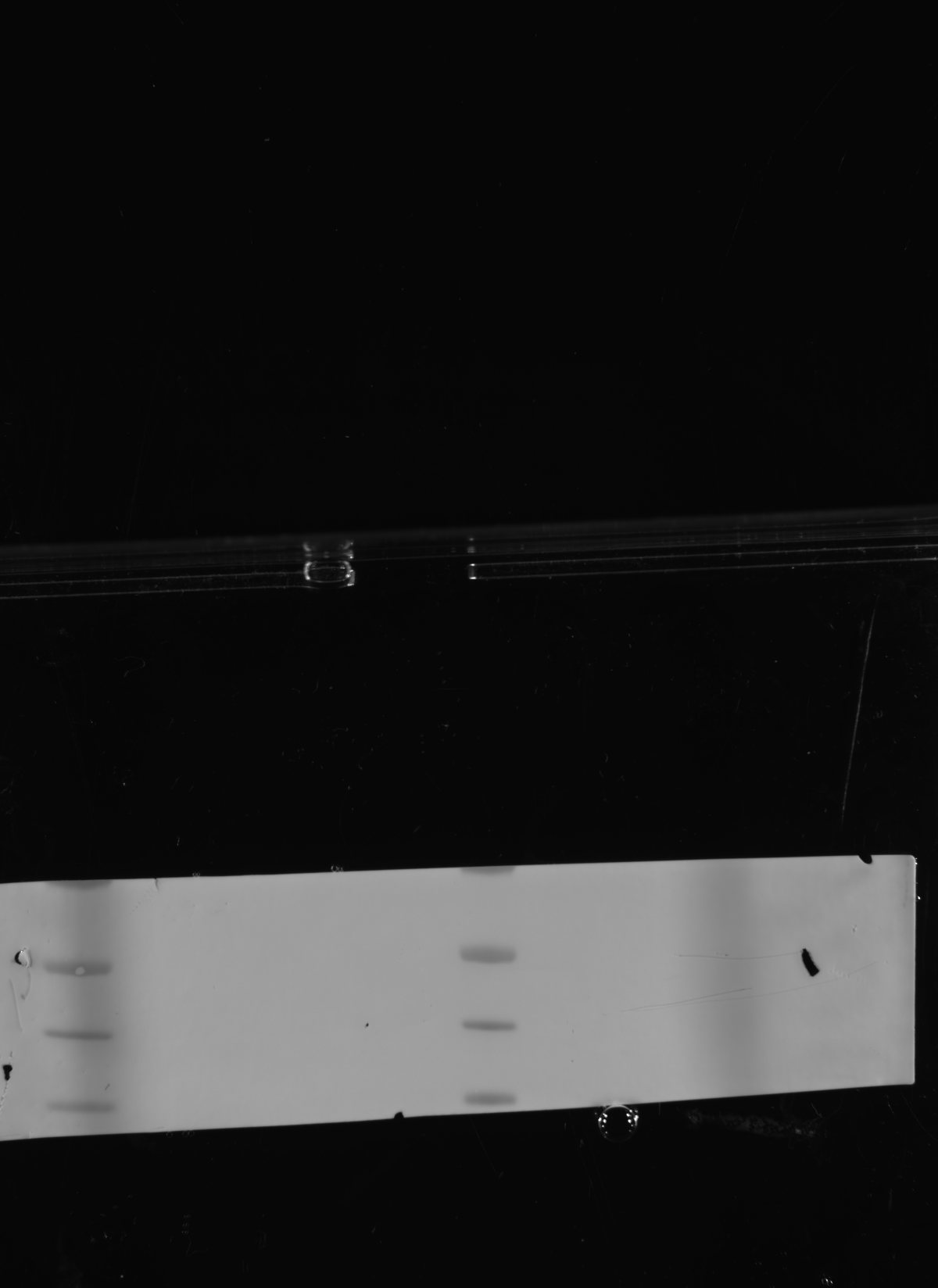

Supplement: Figure 3—source data 3. [file elife-101702-fig3-data3.zip › GAPDH for p-IKK day 3 WL.tif]

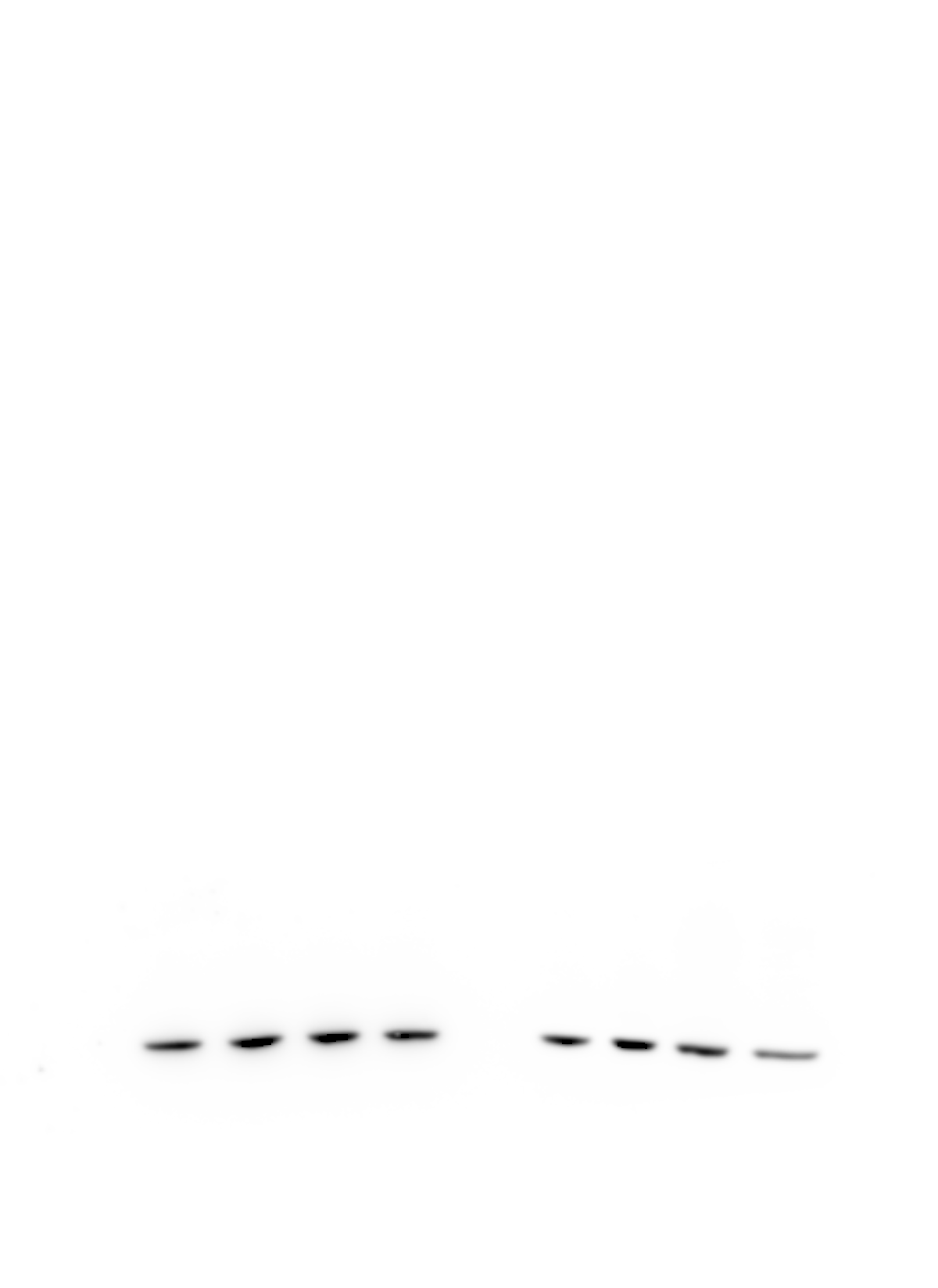

Supplement: Figure 3—source data 3. [file elife-101702-fig3-data3.zip › GAPDH for p-IKK day 3.tif]

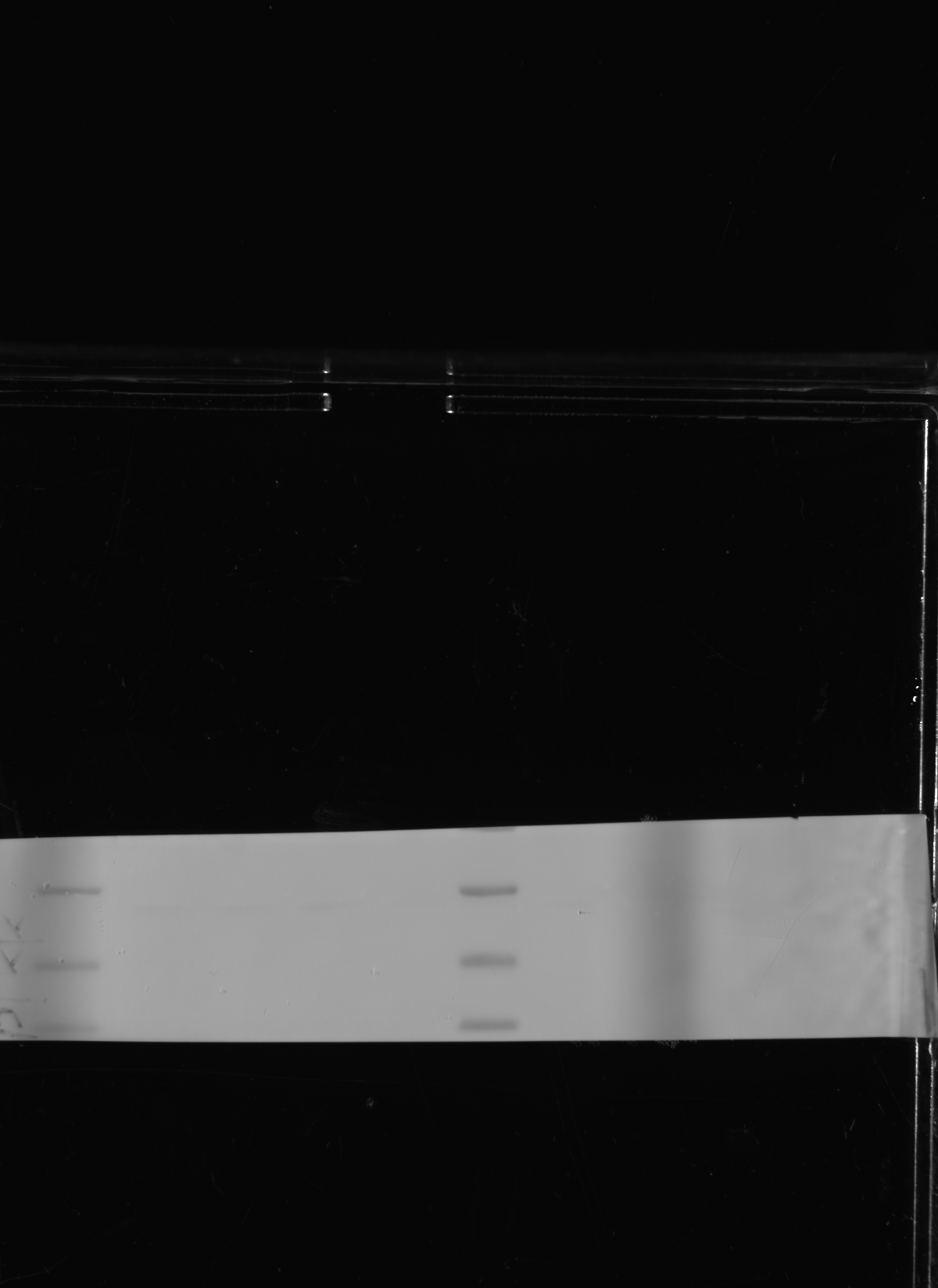

Supplement: Figure 3—source data 3. [file elife-101702-fig3-data3.zip › GAPDH for p-IKK day 5 WL.tif]

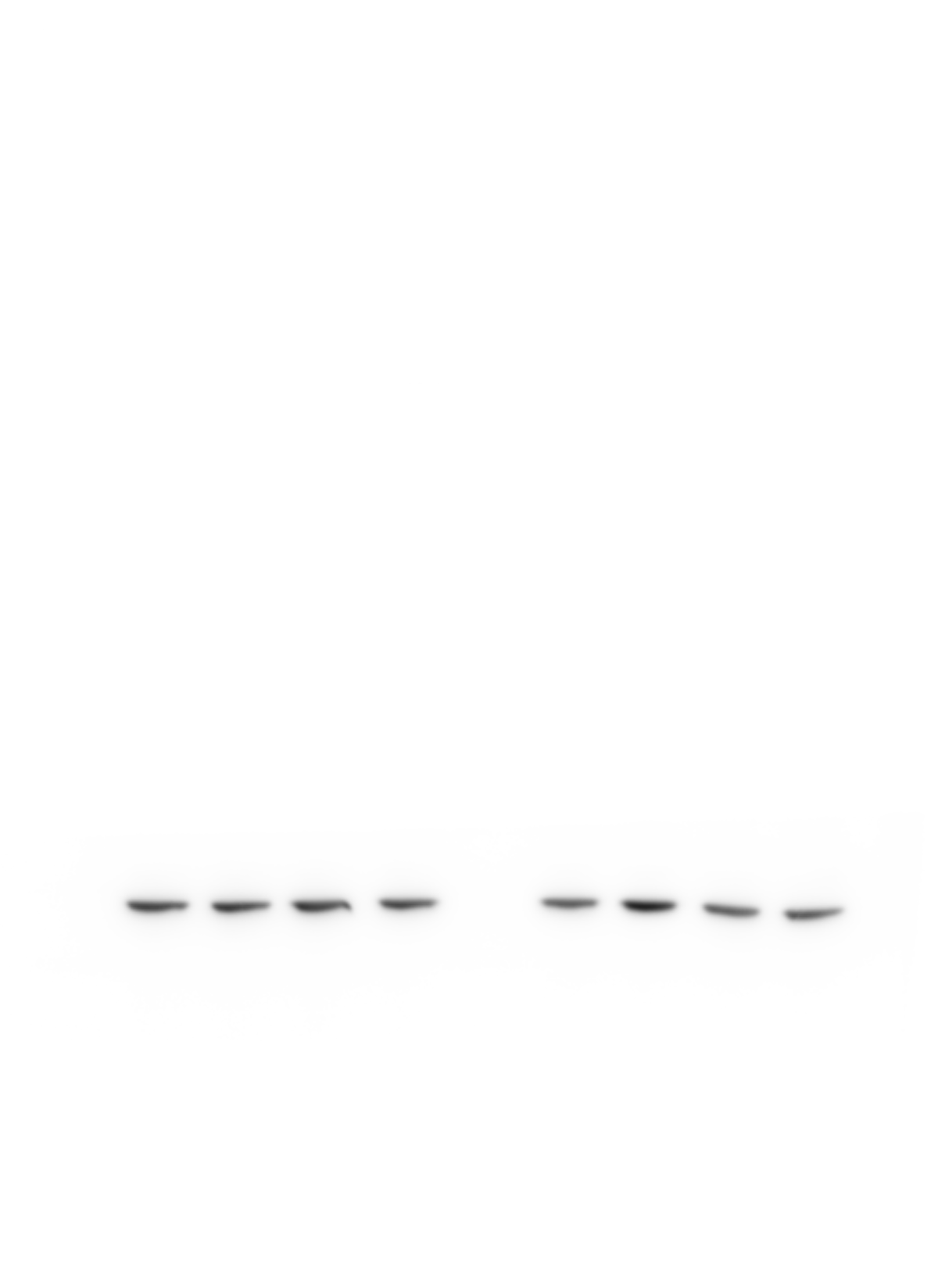

Supplement: Figure 3—source data 3. [file elife-101702-fig3-data3.zip › GAPDH for p-IKK day 5.tif]

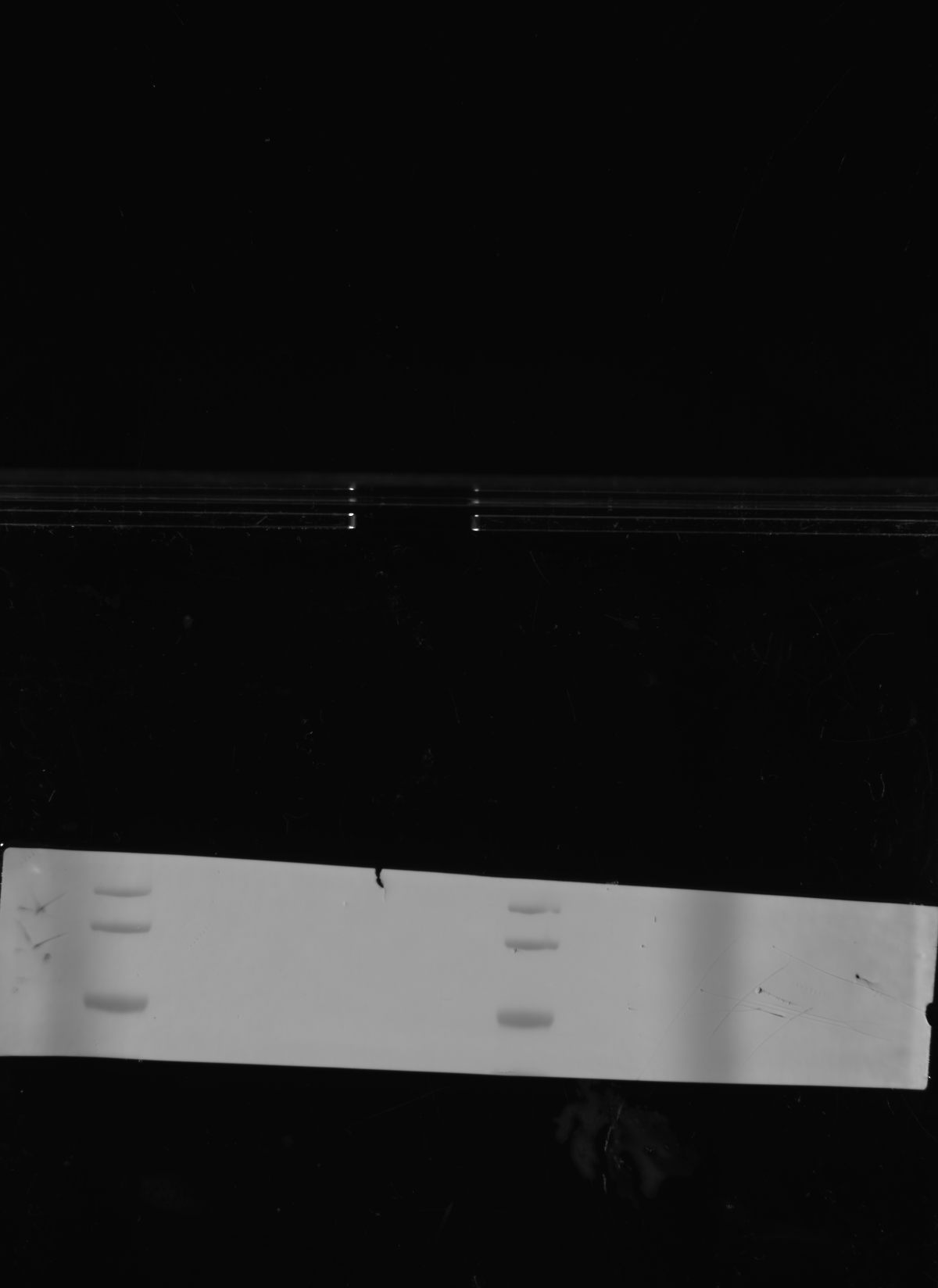

Supplement: Figure 3—source data 3. [file elife-101702-fig3-data3.zip › IKK day 3 WL.tif]

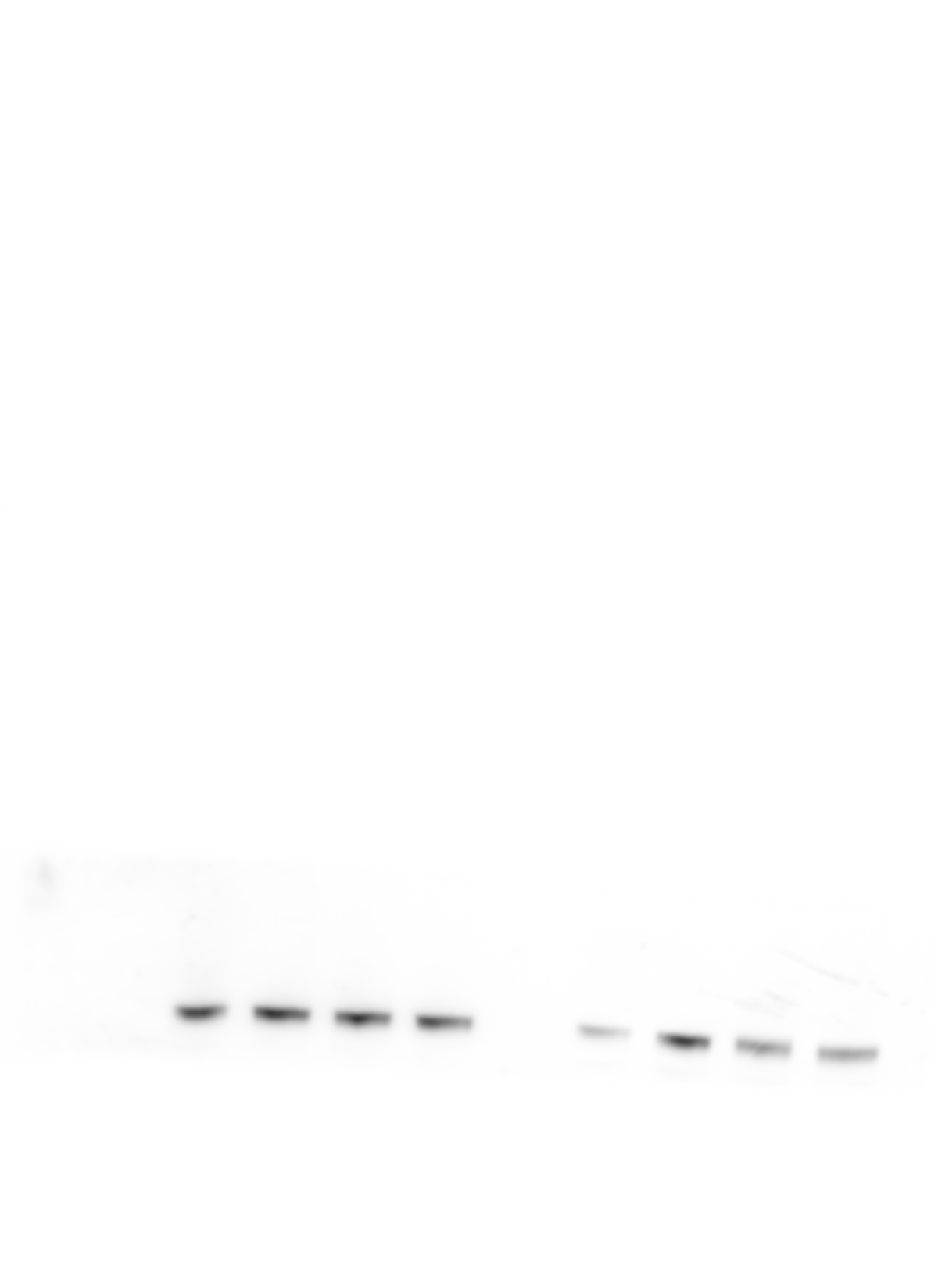

Supplement: Figure 3—source data 3. [file elife-101702-fig3-data3.zip › IKK day 3.tif]

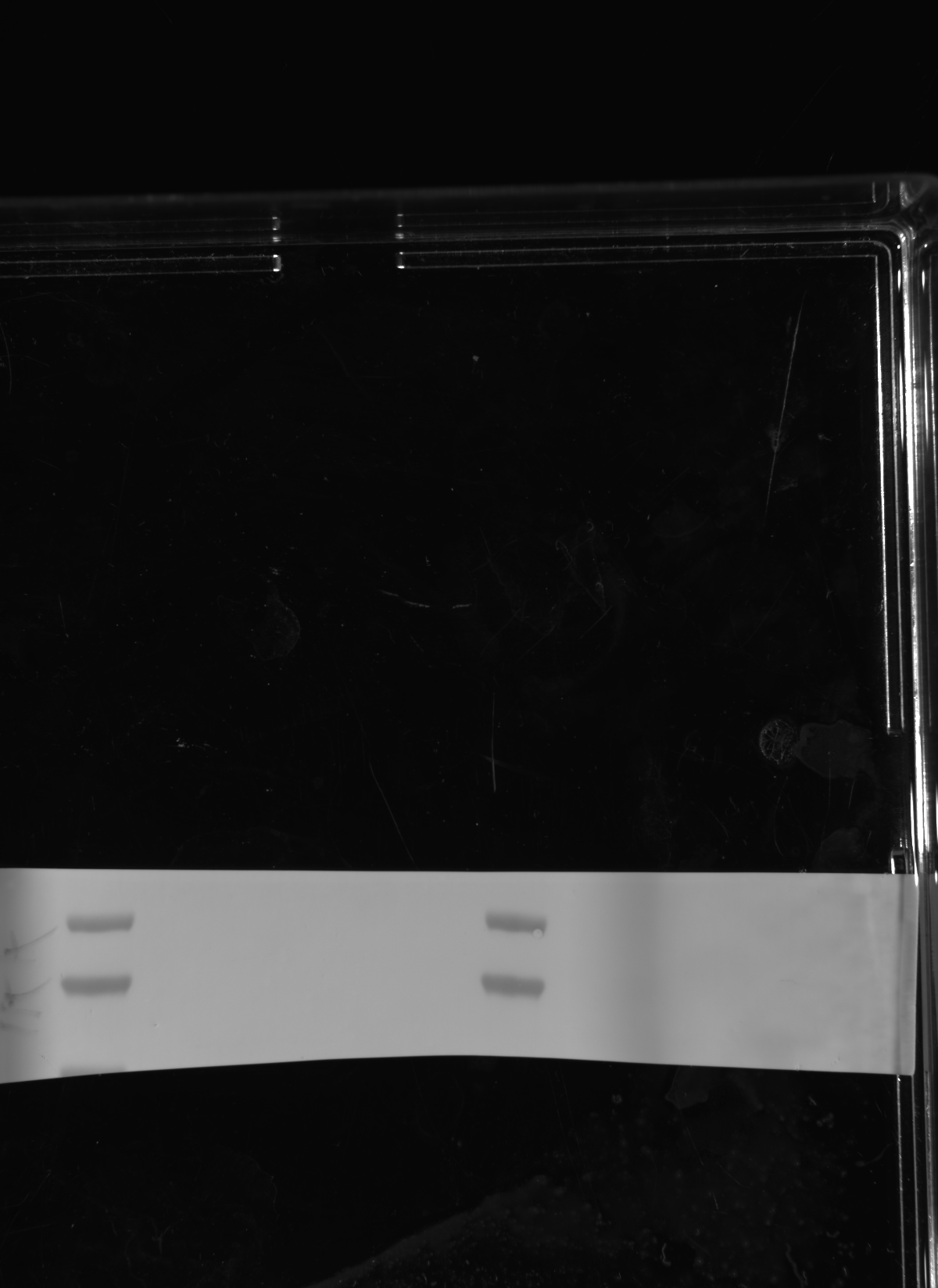

Supplement: Figure 3—source data 3. [file elife-101702-fig3-data3.zip › IKK day 5 WL.tif]

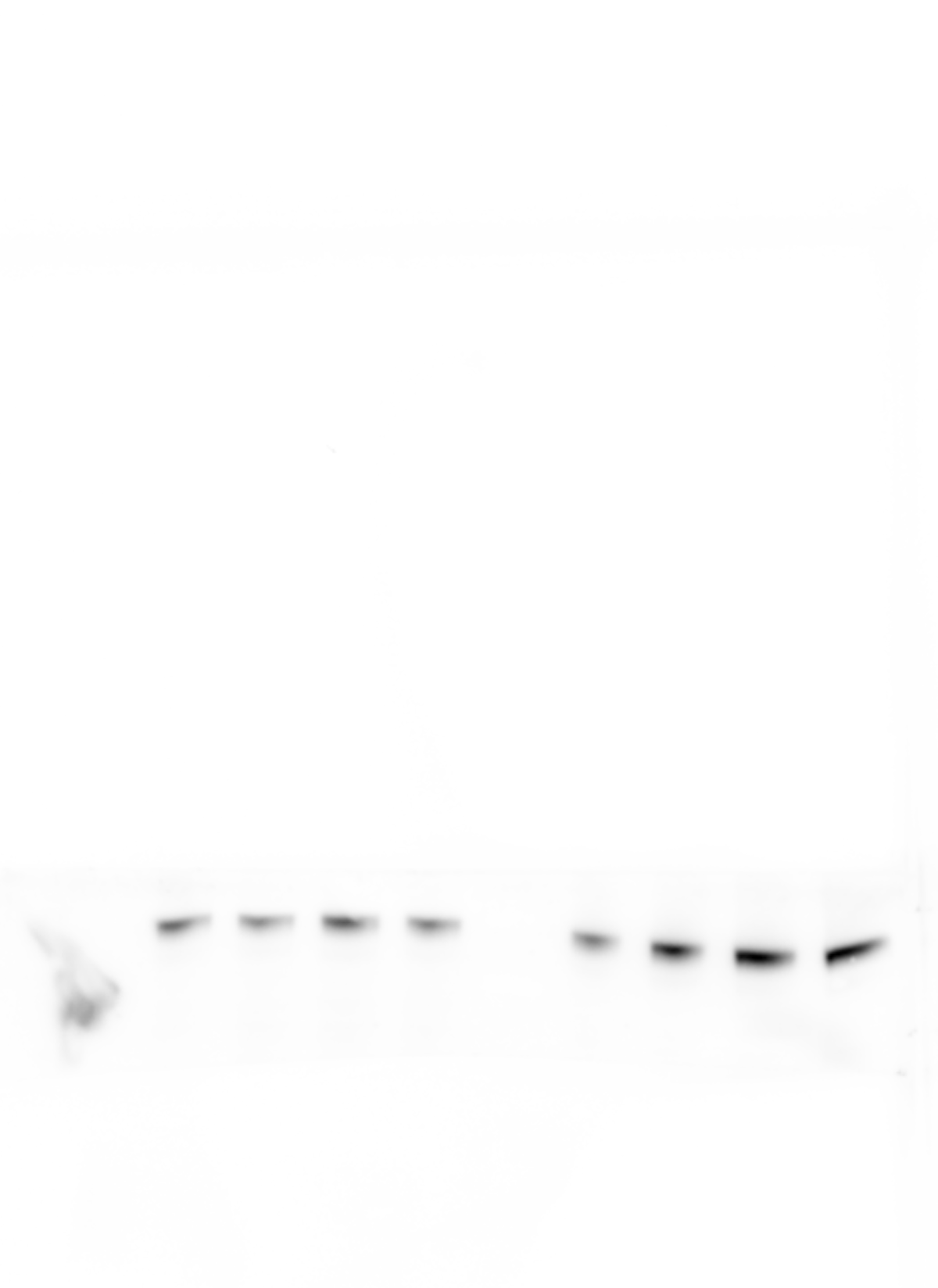

Supplement: Figure 3—source data 3. [file elife-101702-fig3-data3.zip › IKK day 5.tif]

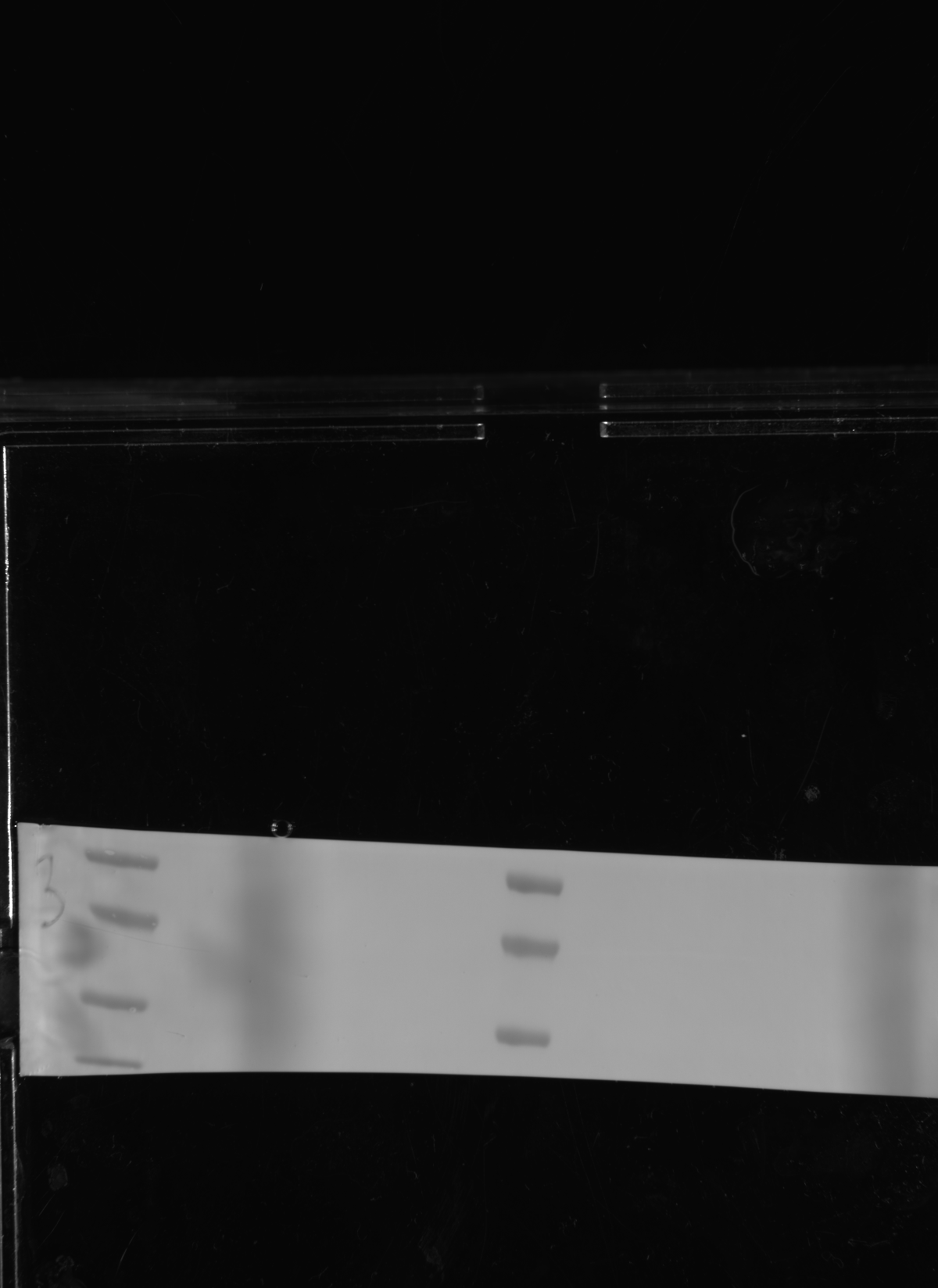

Supplement: Figure 3—source data 3. [file elife-101702-fig3-data3.zip › NF-kB day 3 WL.tif]

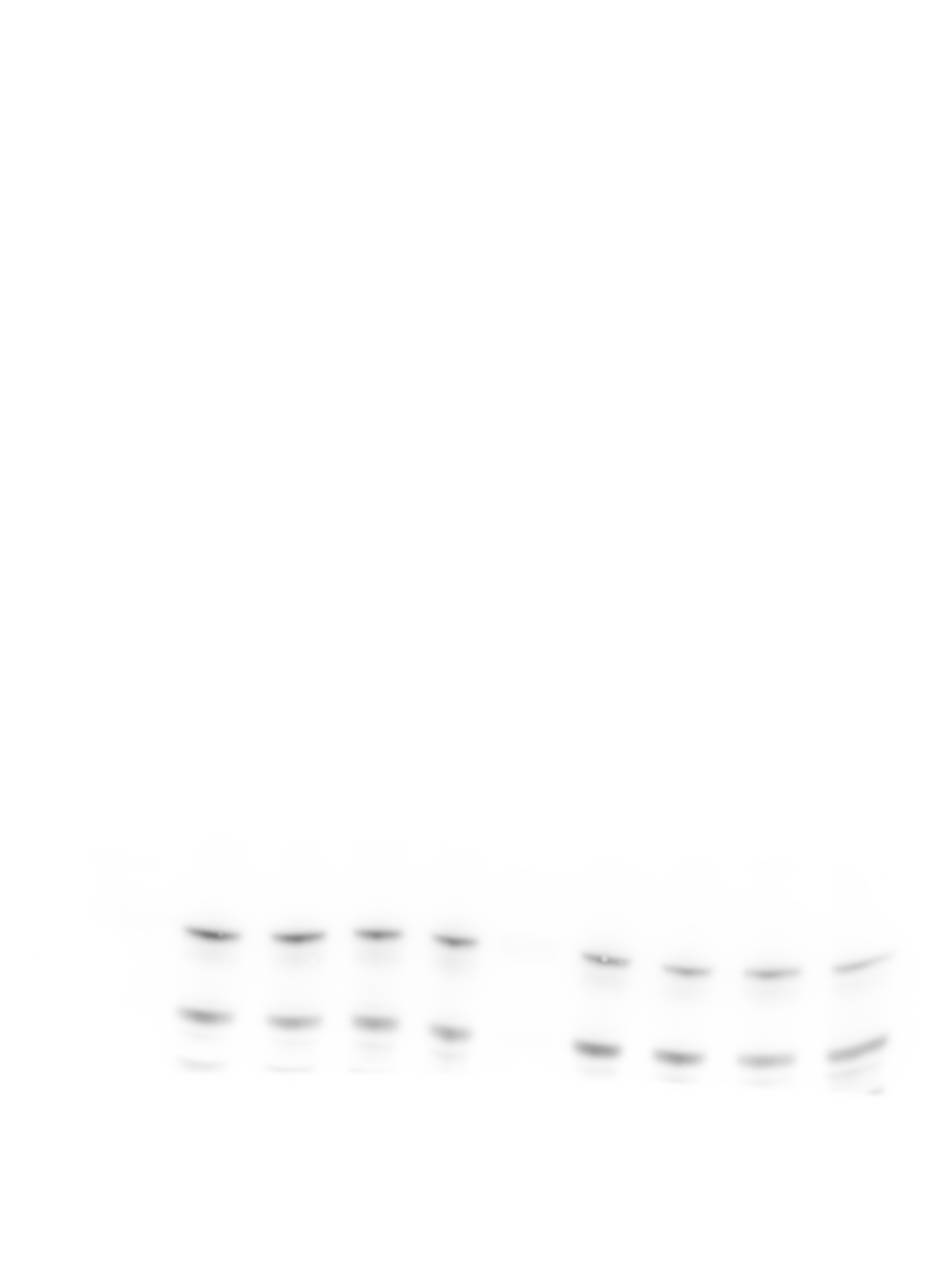

Supplement: Figure 3—source data 3. [file elife-101702-fig3-data3.zip › NF-kB day 3.tif]

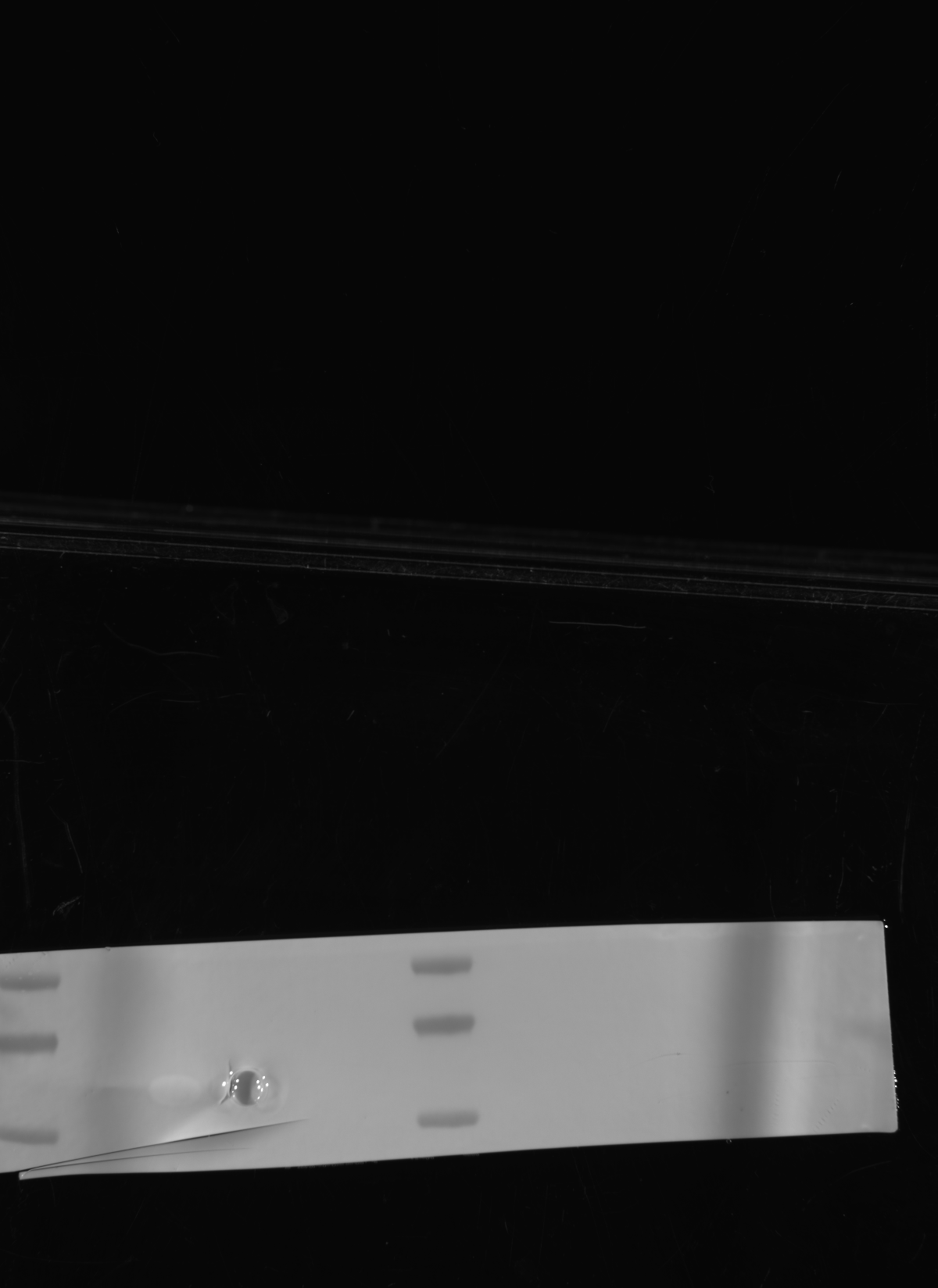

Supplement: Figure 3—source data 3. [file elife-101702-fig3-data3.zip › NF-kB day 5 WL.tif]

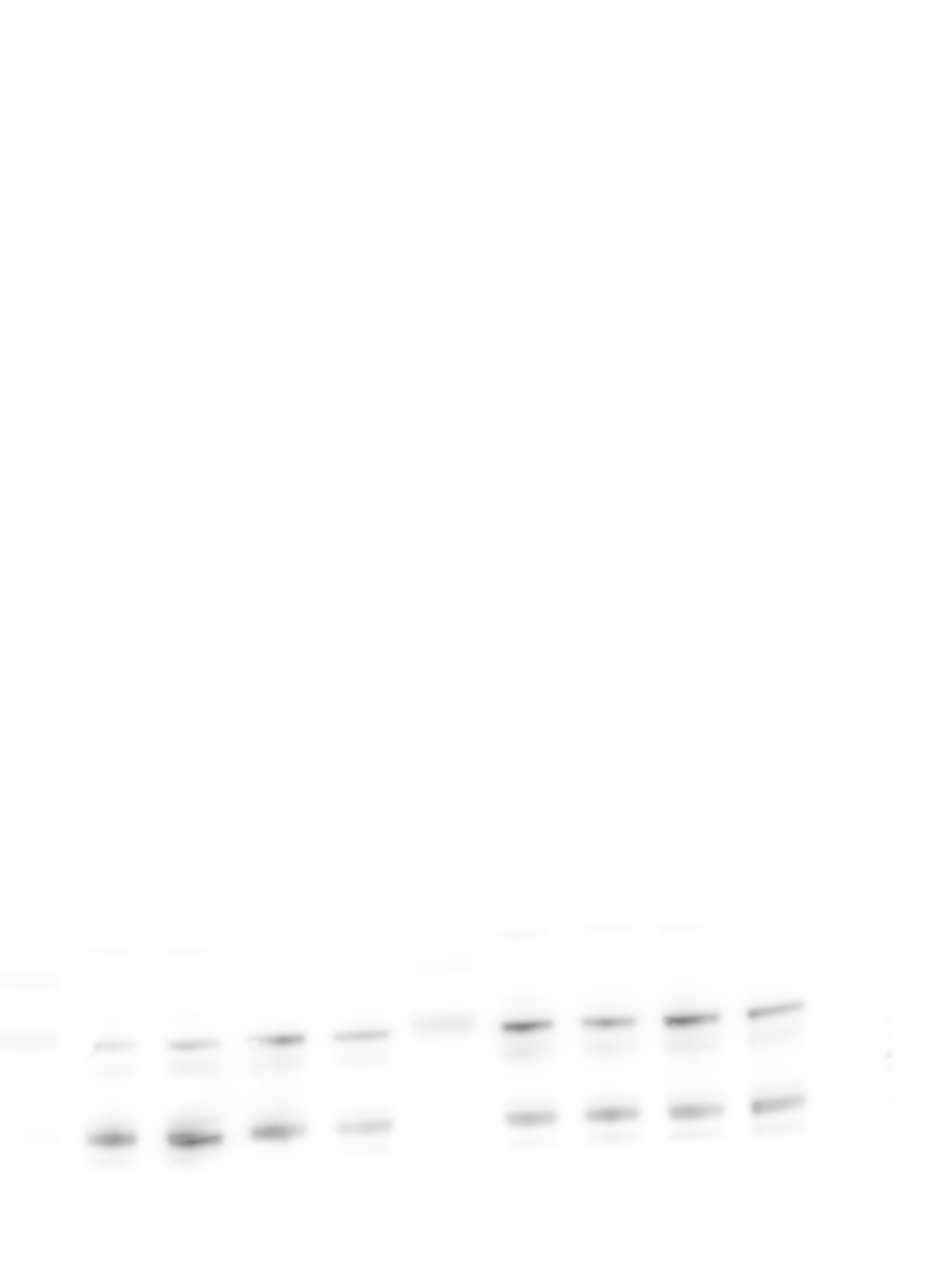

Supplement: Figure 3—source data 3. [file elife-101702-fig3-data3.zip › NF-kB day 5.tif]

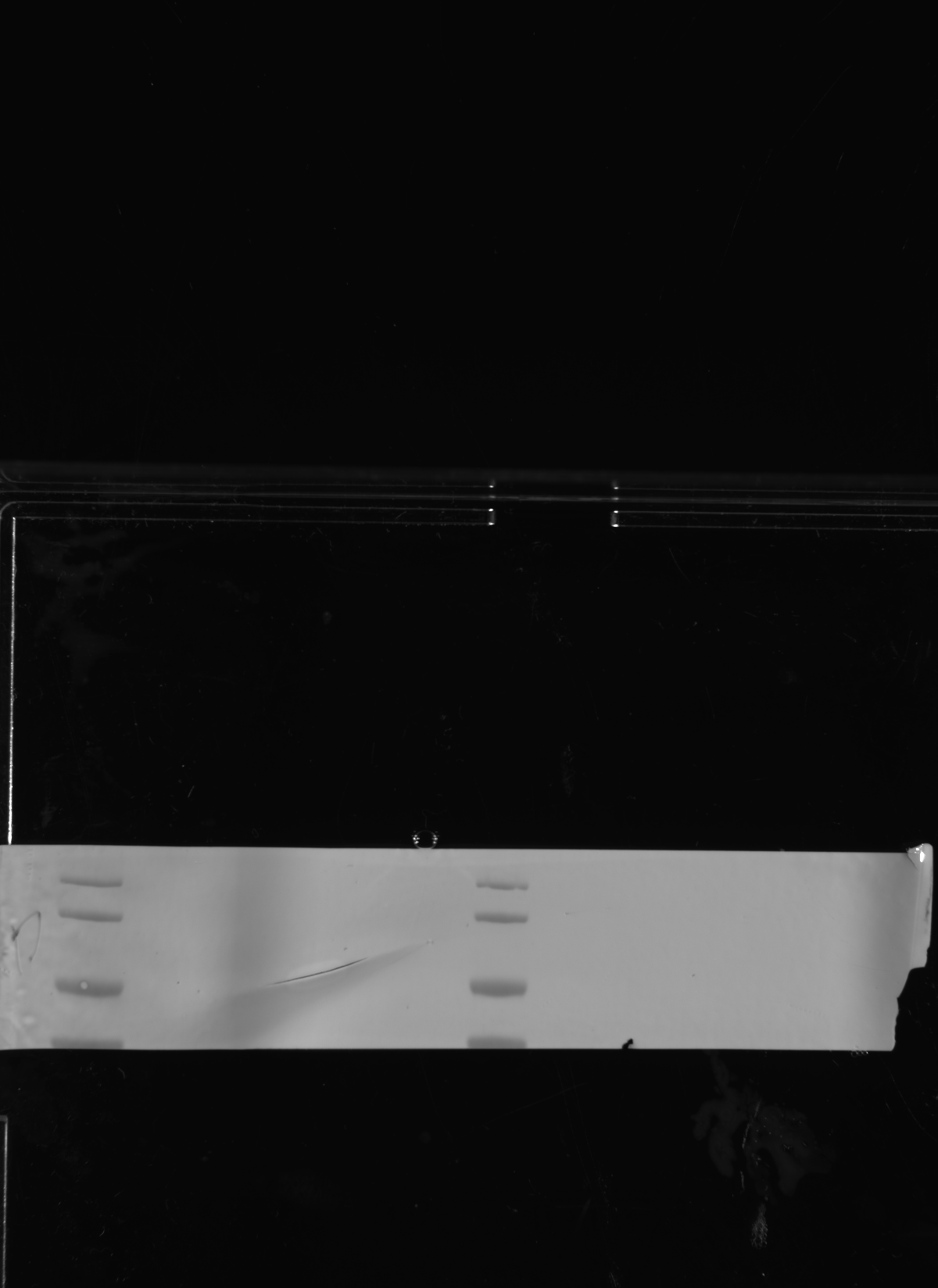

Supplement: Figure 3—source data 3. [file elife-101702-fig3-data3.zip › p-IKK day 3 WL.tif]

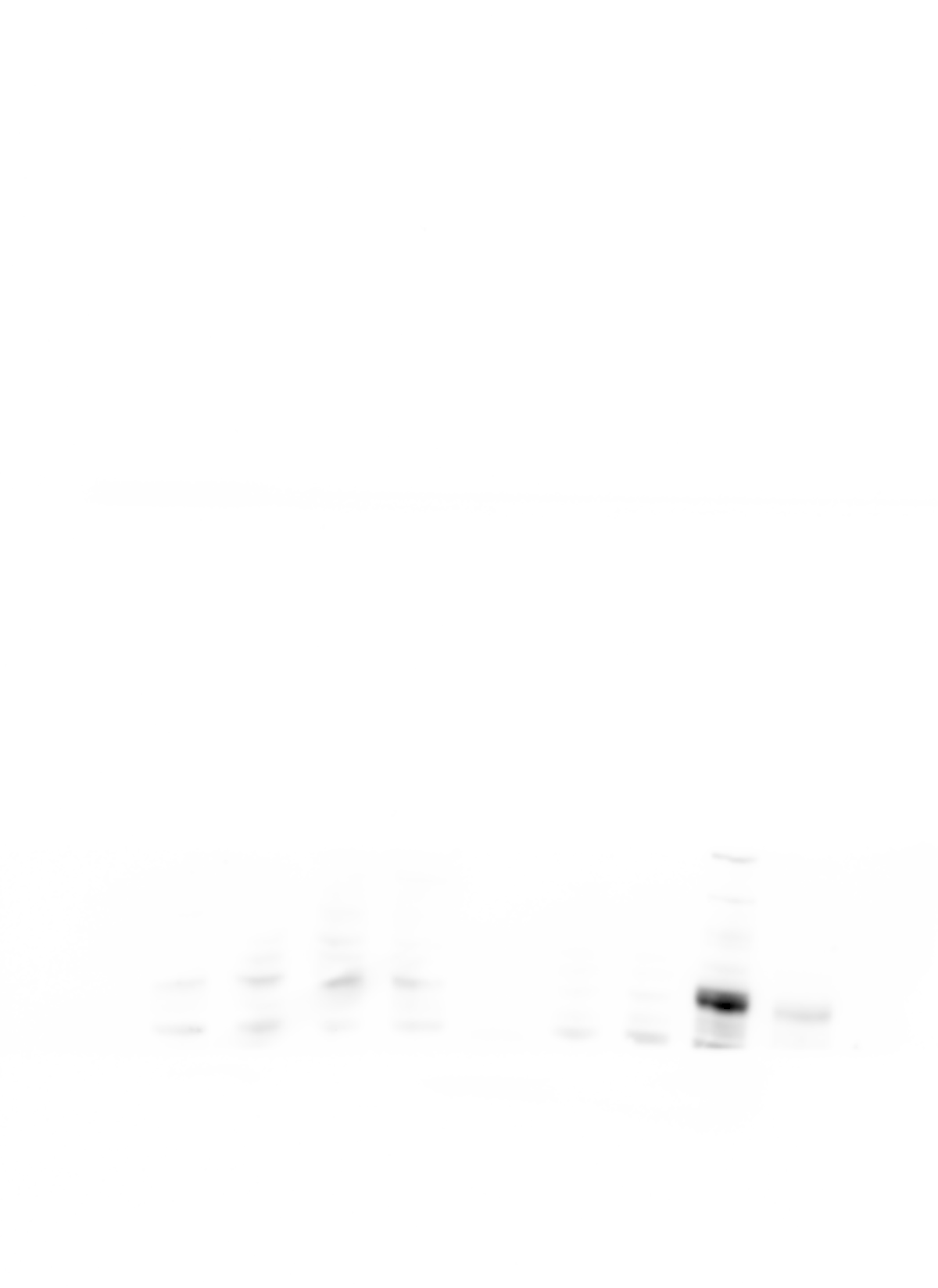

Supplement: Figure 3—source data 3. [file elife-101702-fig3-data3.zip › p-IKK day 3.tif]

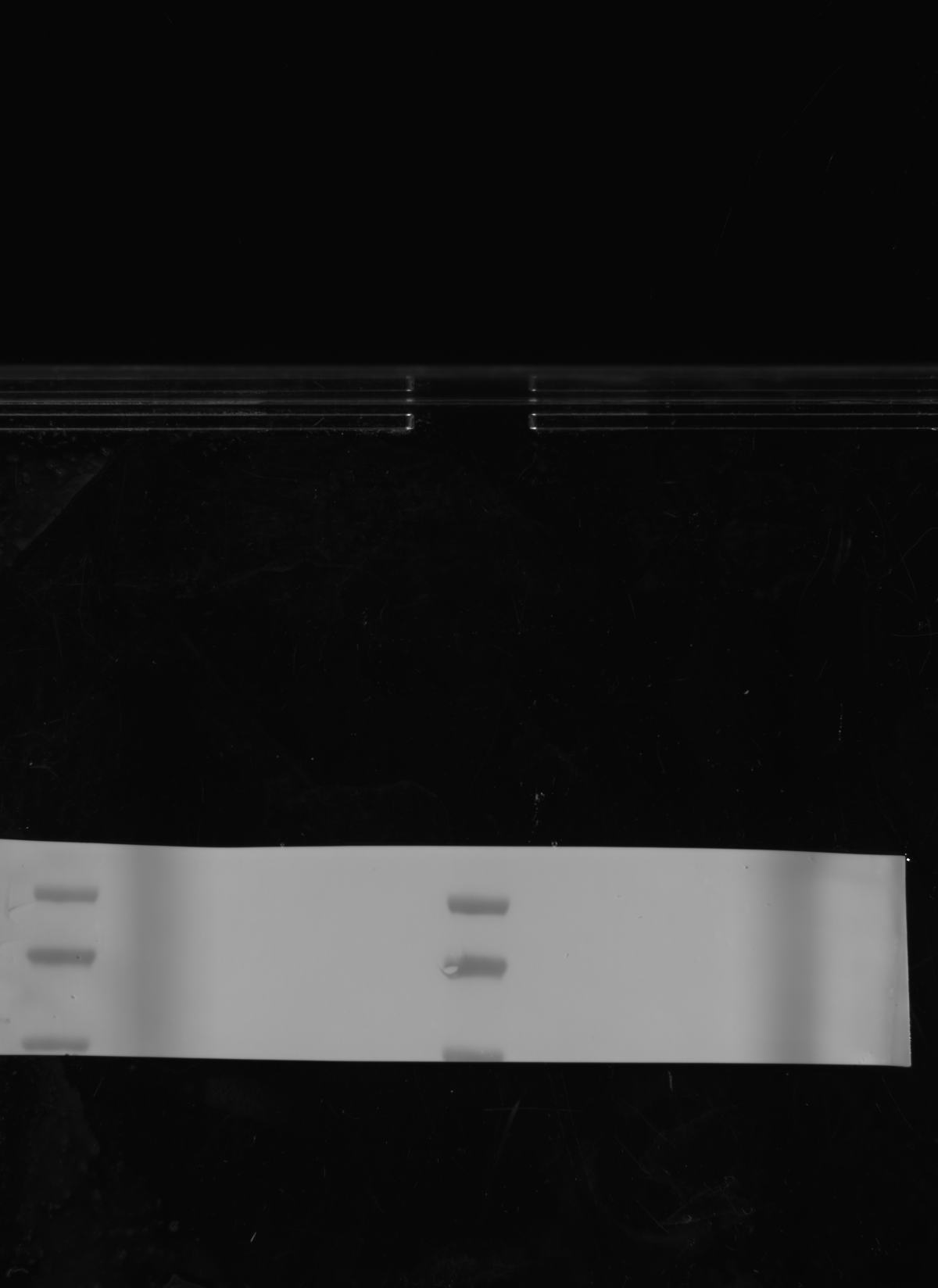

Supplement: Figure 3—source data 3. [file elife-101702-fig3-data3.zip › p-IKK day 5 WL.tif]

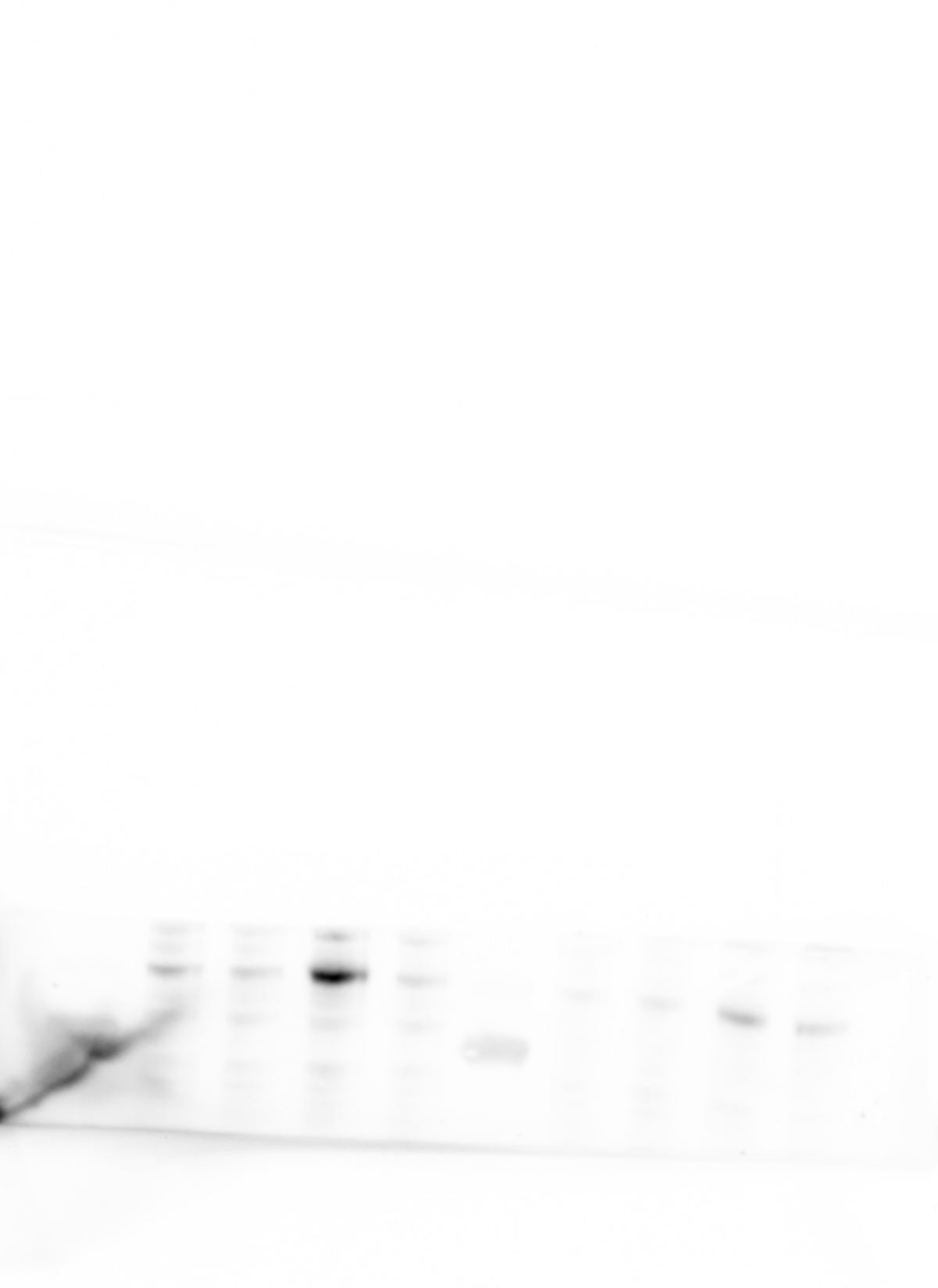

Supplement: Figure 3—source data 3. [file elife-101702-fig3-data3.zip › p-IKK day 5.tif]

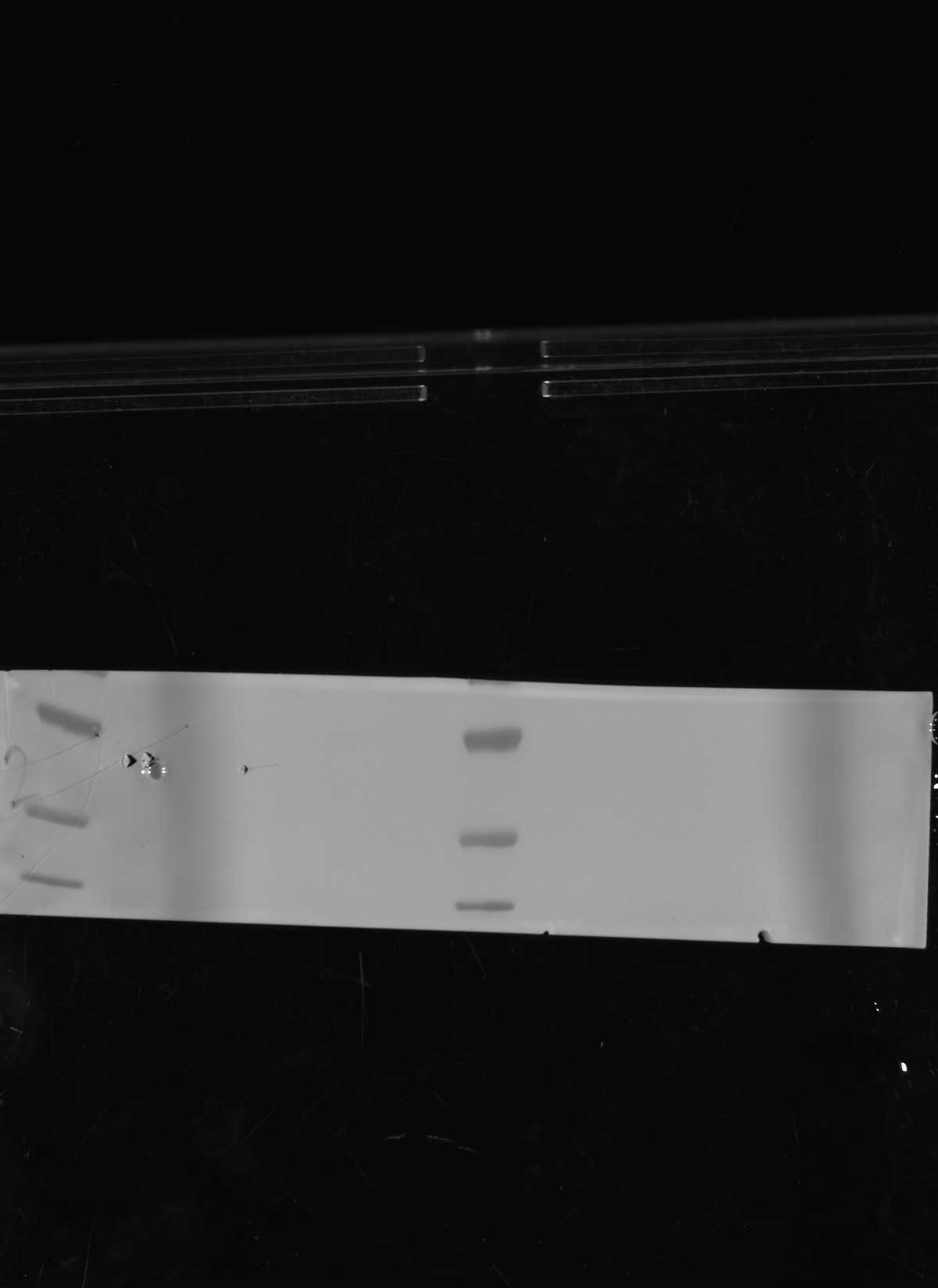

Supplement: Figure 3—source data 3. [file elife-101702-fig3-data3.zip › p-NF-kB day 3 WL.tif]

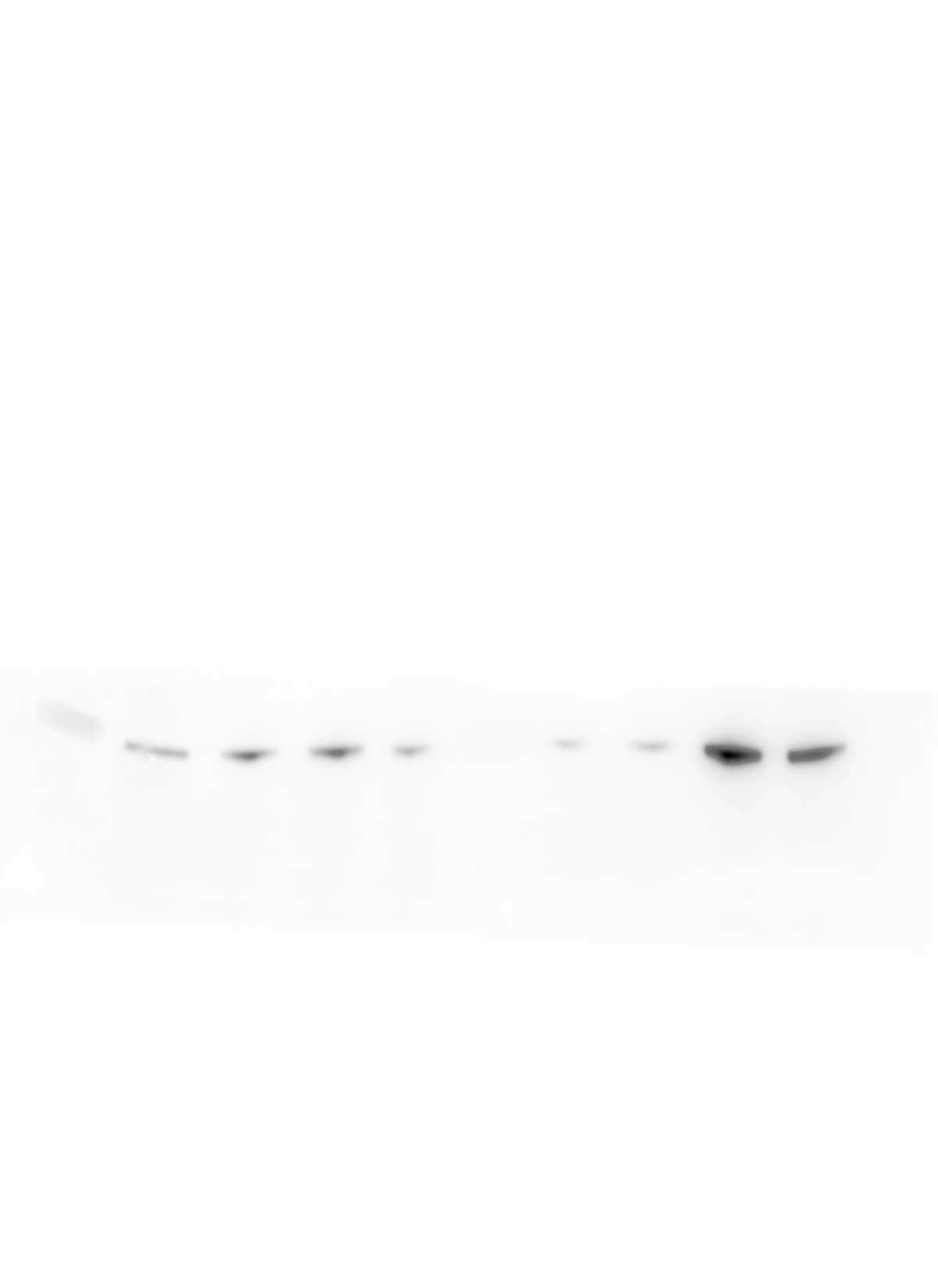

Supplement: Figure 3—source data 3. [file elife-101702-fig3-data3.zip › p-NF-kB day 3.tif]

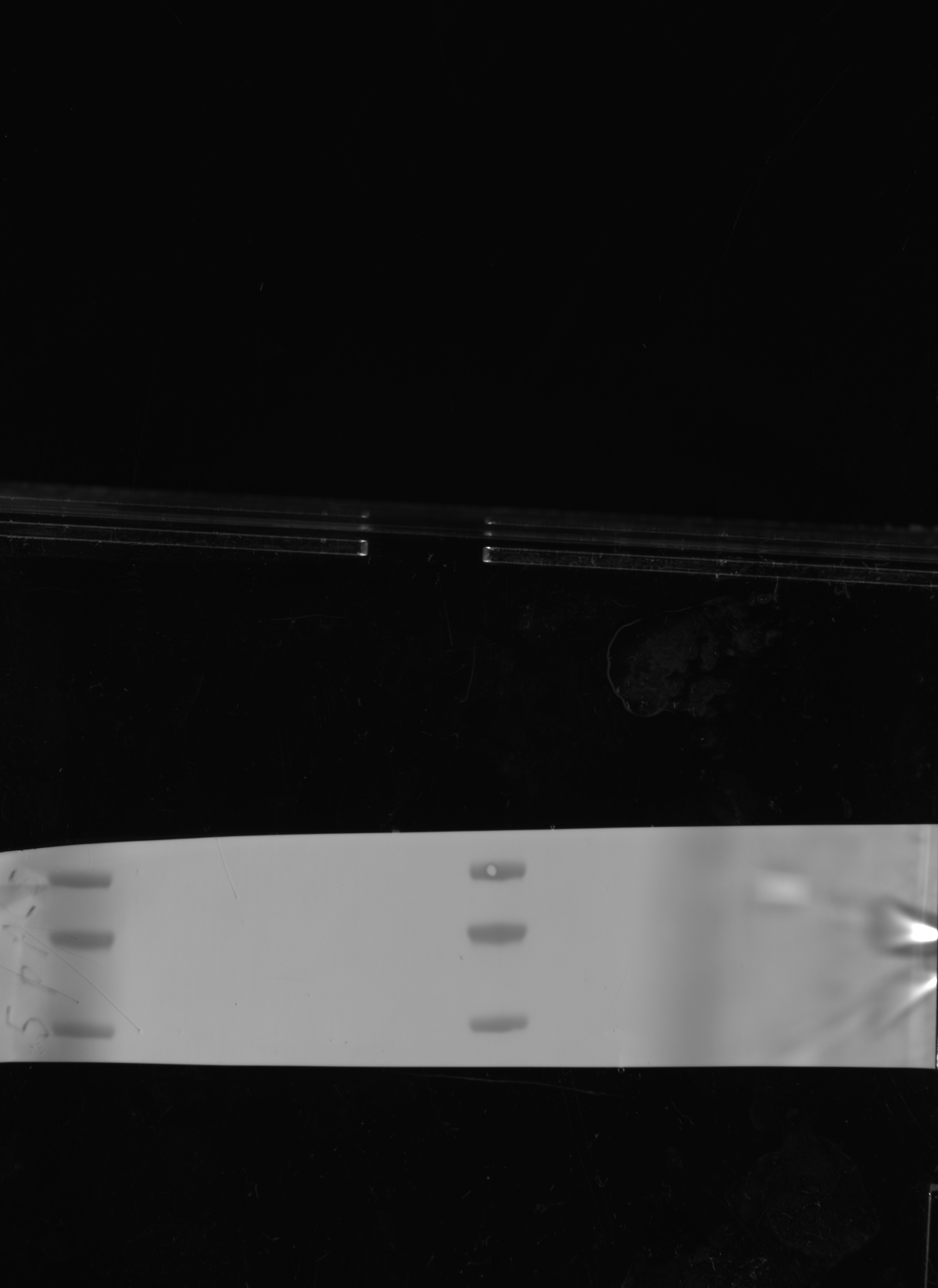

Supplement: Figure 3—source data 3. [file elife-101702-fig3-data3.zip › p-NF-kB day 5 WL.tif]

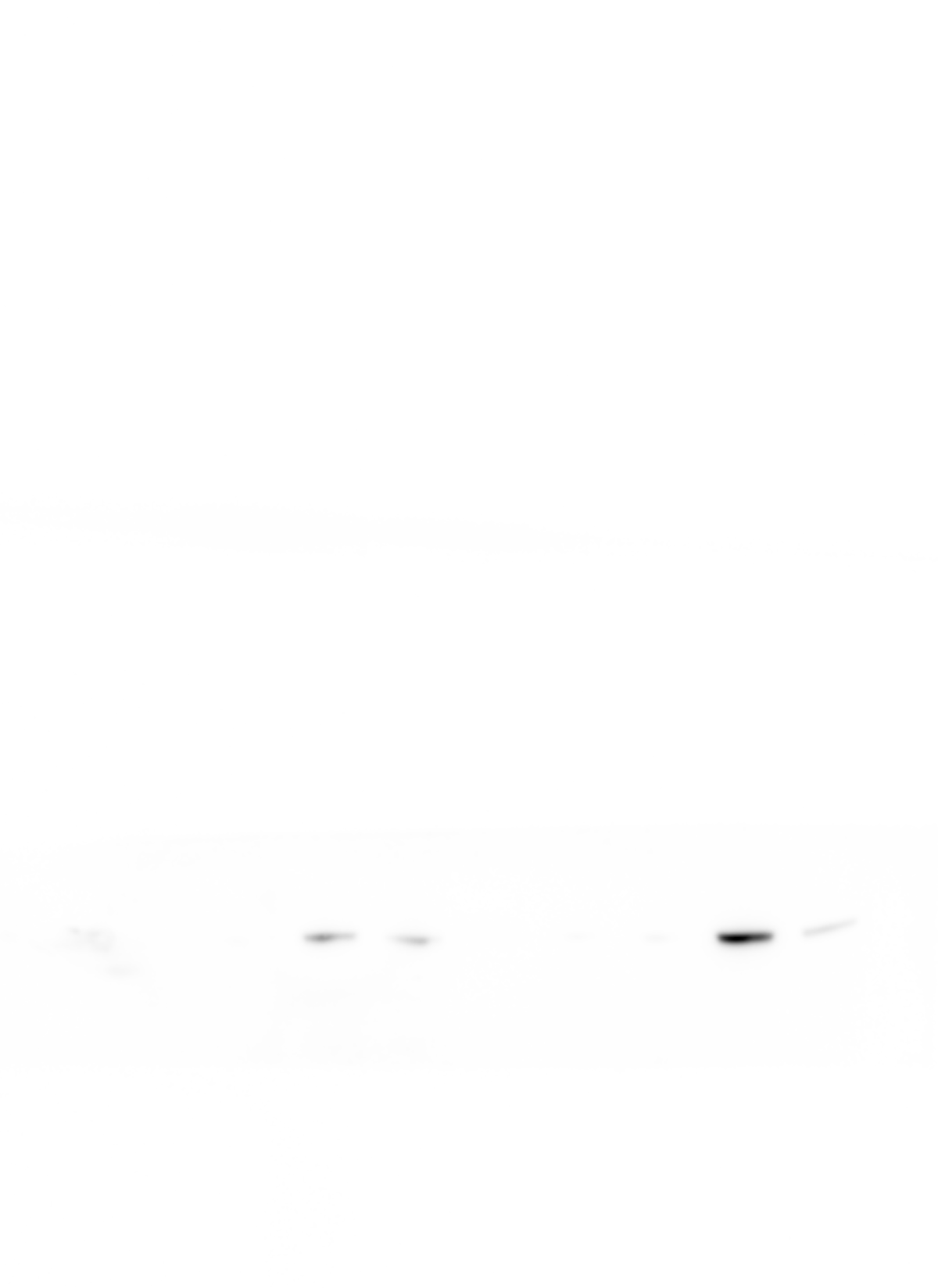

Supplement: Figure 3—source data 3. [file elife-101702-fig3-data3.zip › p-NF-kB day 5.tif]

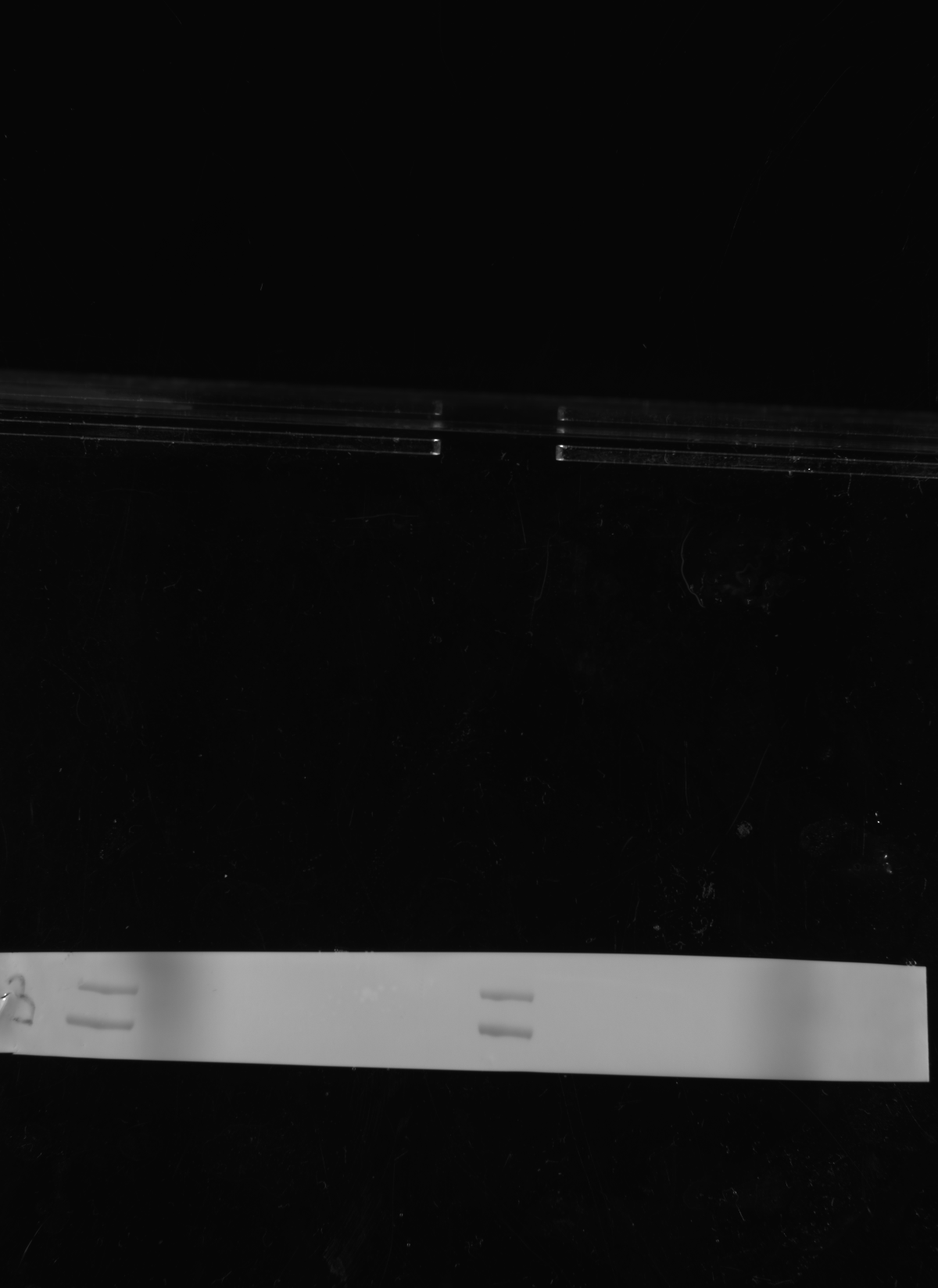

Supplement: Figure 3—source data 3. [file elife-101702-fig3-data3.zip › vinculin for NF-kB day 3 WL.tif]

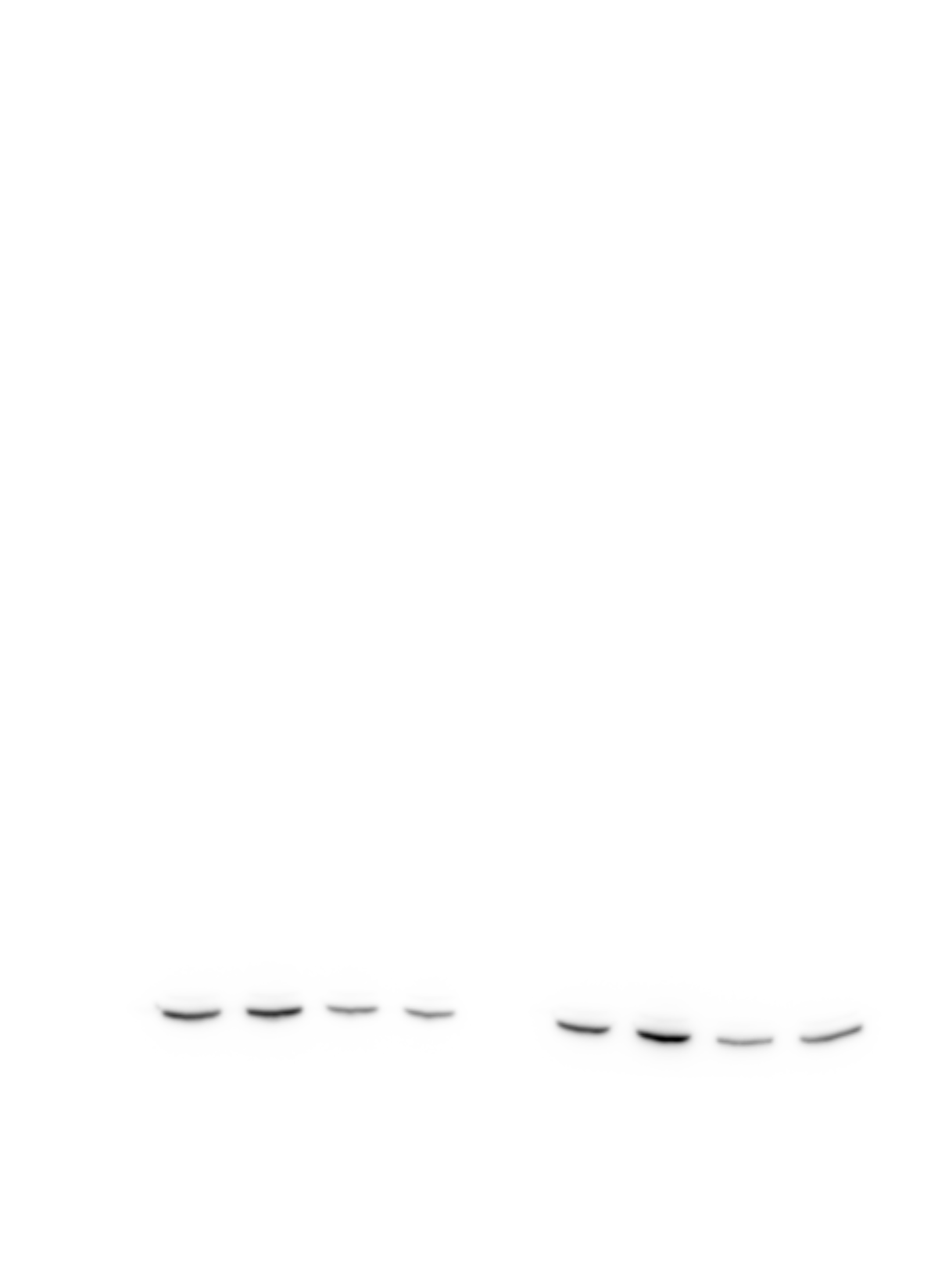

Supplement: Figure 3—source data 3. [file elife-101702-fig3-data3.zip › vinculin for NF-kB day 3.tif]

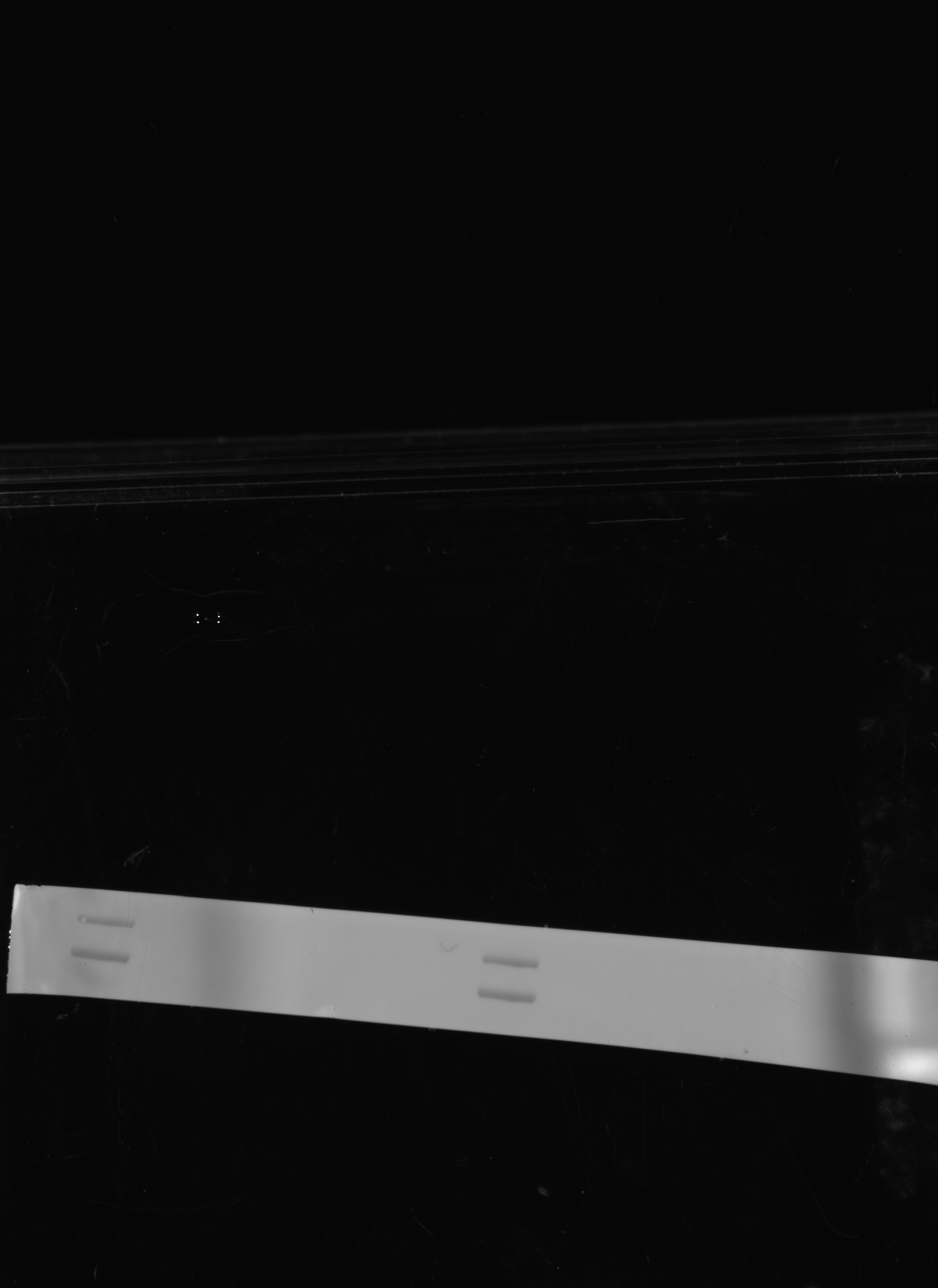

Supplement: Figure 3—source data 3. [file elife-101702-fig3-data3.zip › vinculin for NF-kB day 5 WL.tif]

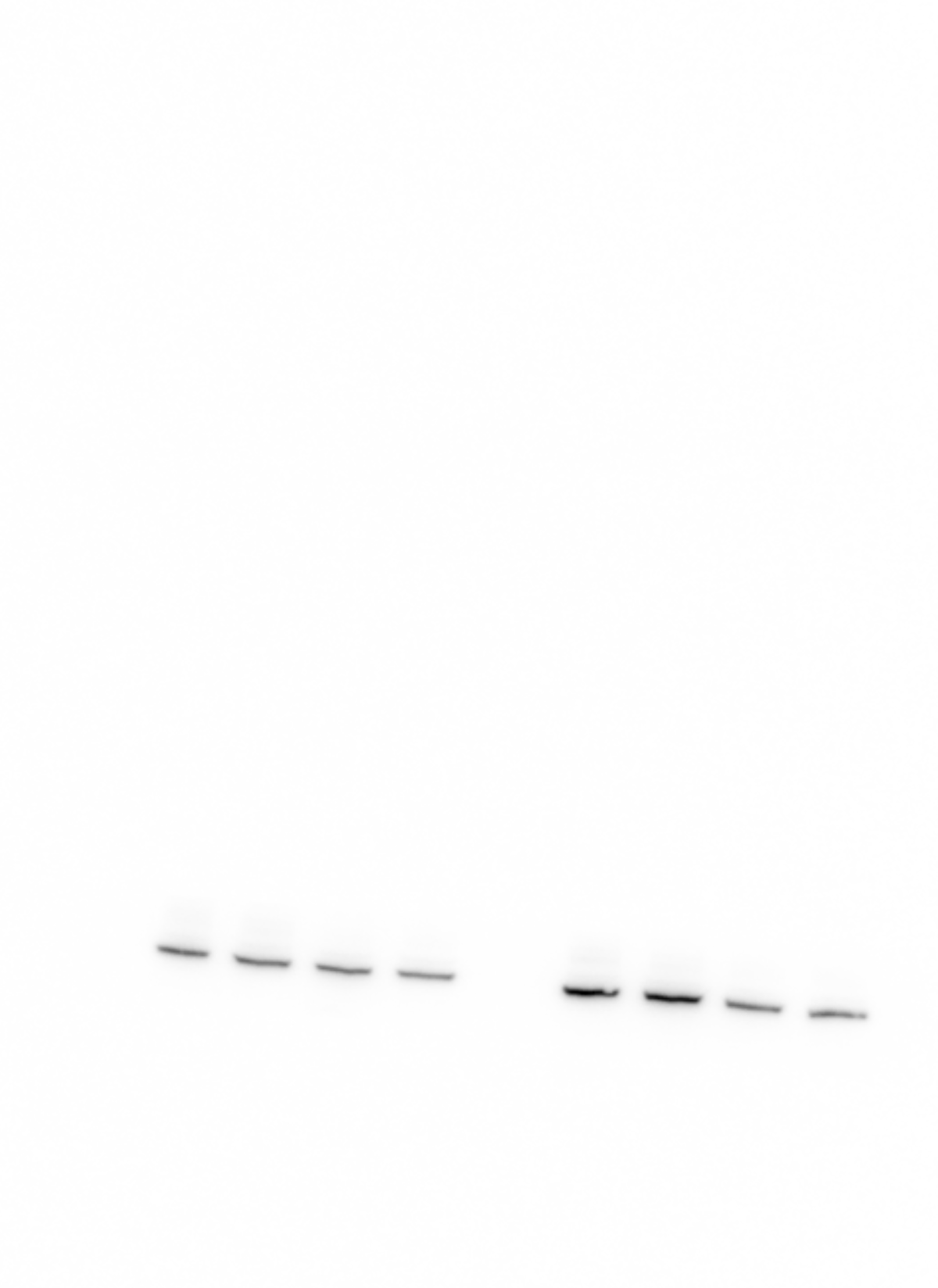

Supplement: Figure 3—source data 3. [file elife-101702-fig3-data3.zip › vinculin for NF-kB day 5.tif]

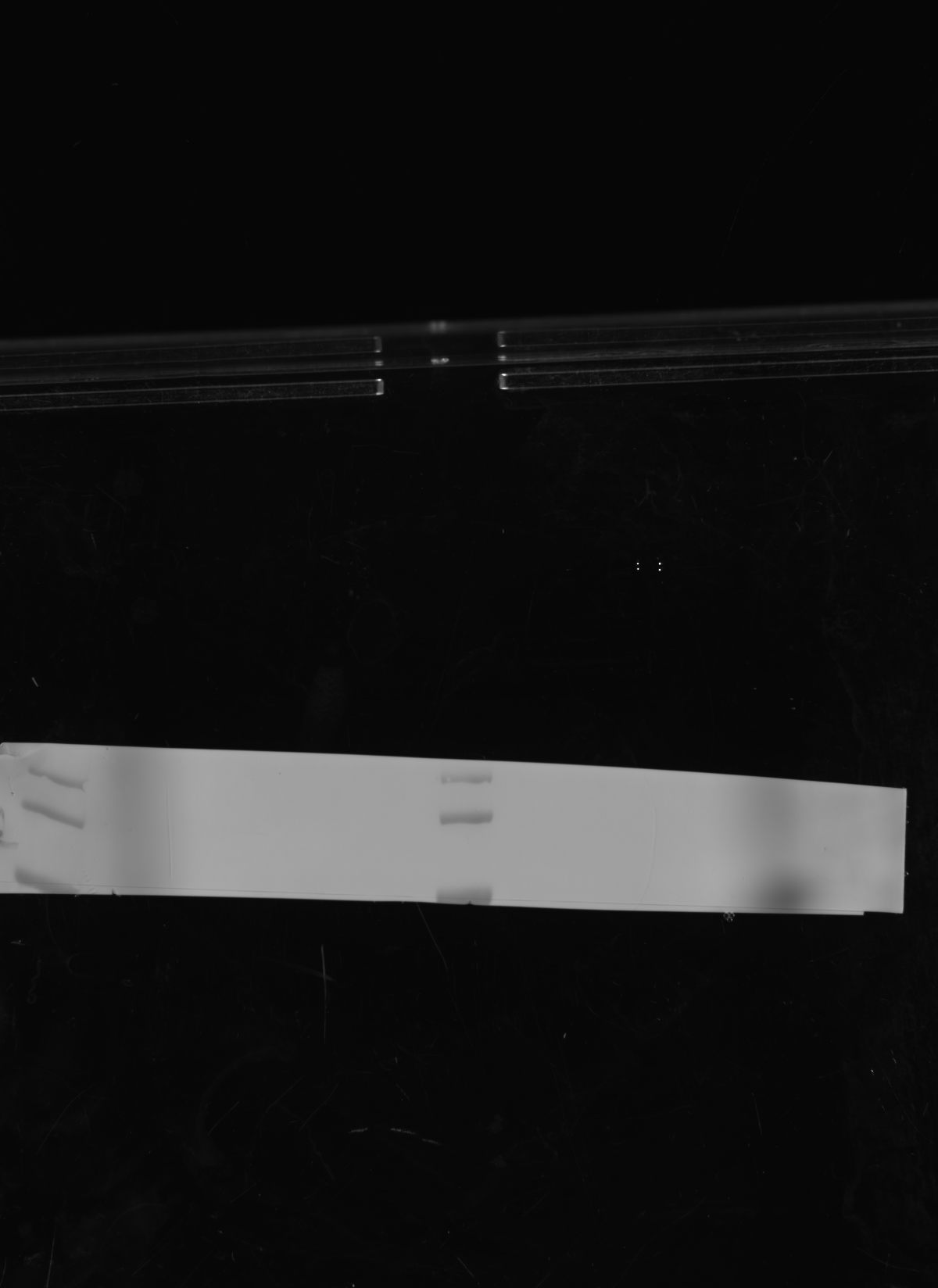

Supplement: Figure 3—source data 3. [file elife-101702-fig3-data3.zip › vinculin for p-NF-kB day 3 WL.tif]

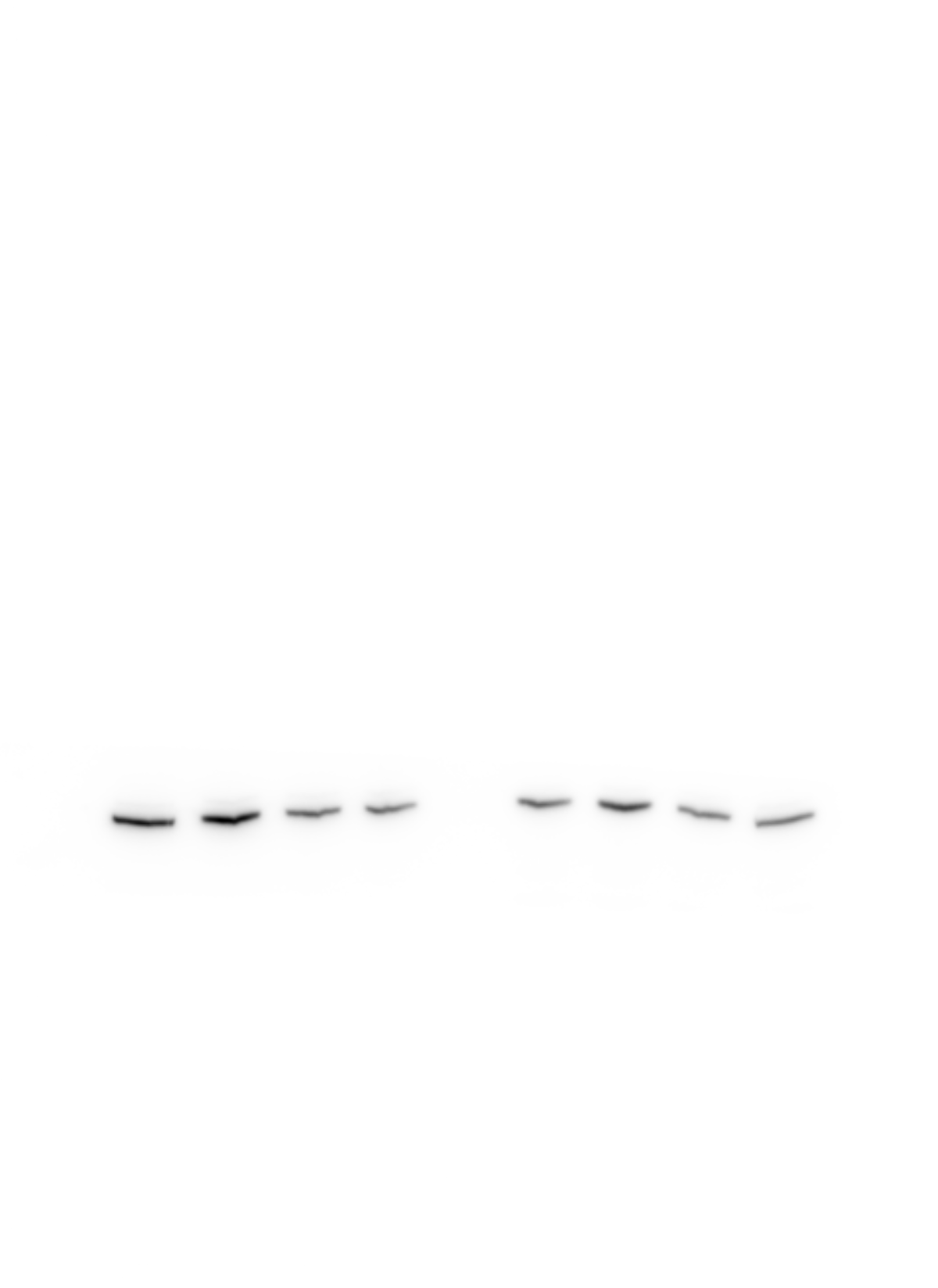

Supplement: Figure 3—source data 3. [file elife-101702-fig3-data3.zip › vinculin for p-NF-kB day 3.tif]

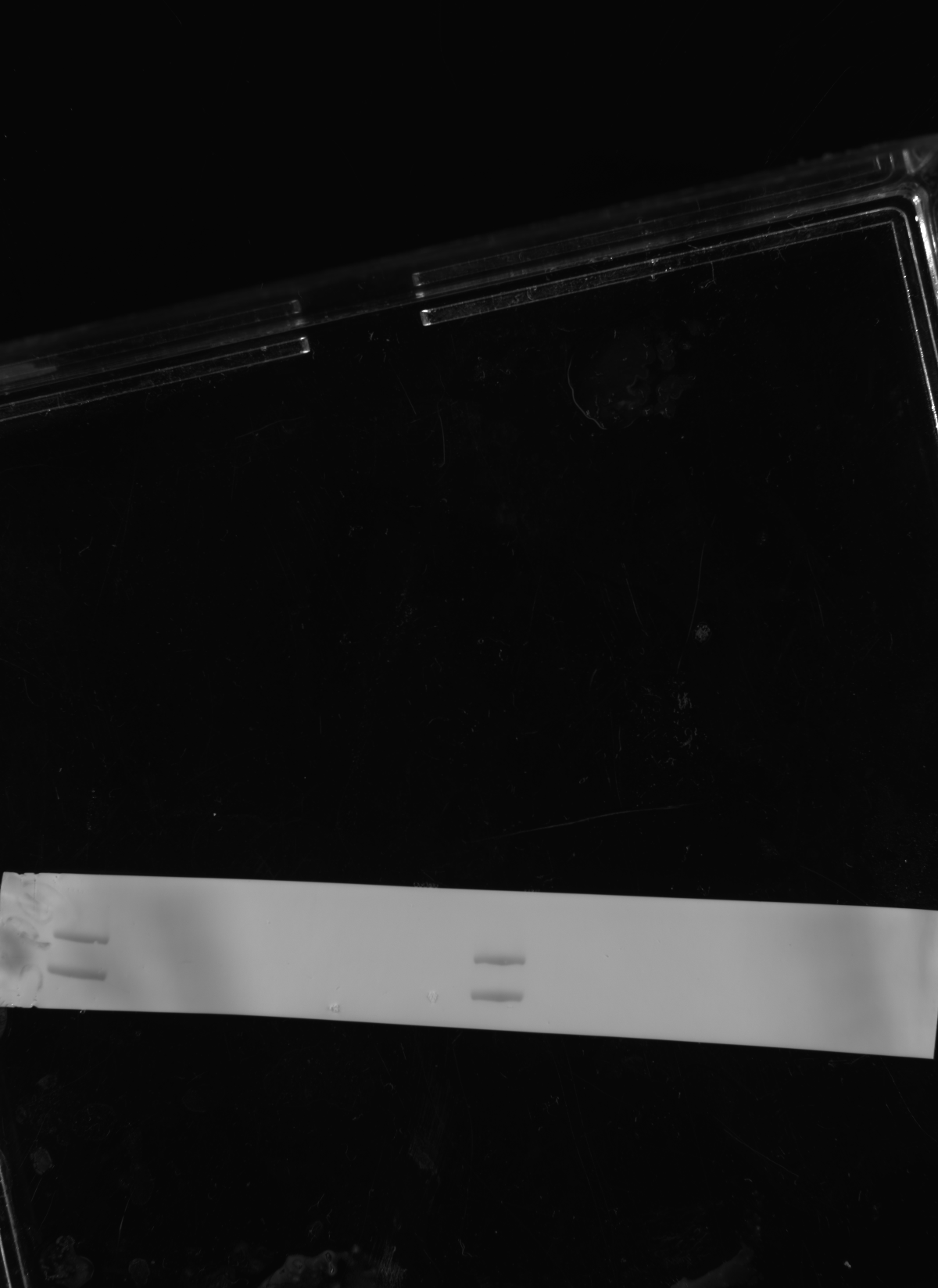

Supplement: Figure 3—source data 3. [file elife-101702-fig3-data3.zip › vinculin for p-NF-kB day 5 WL.tif]

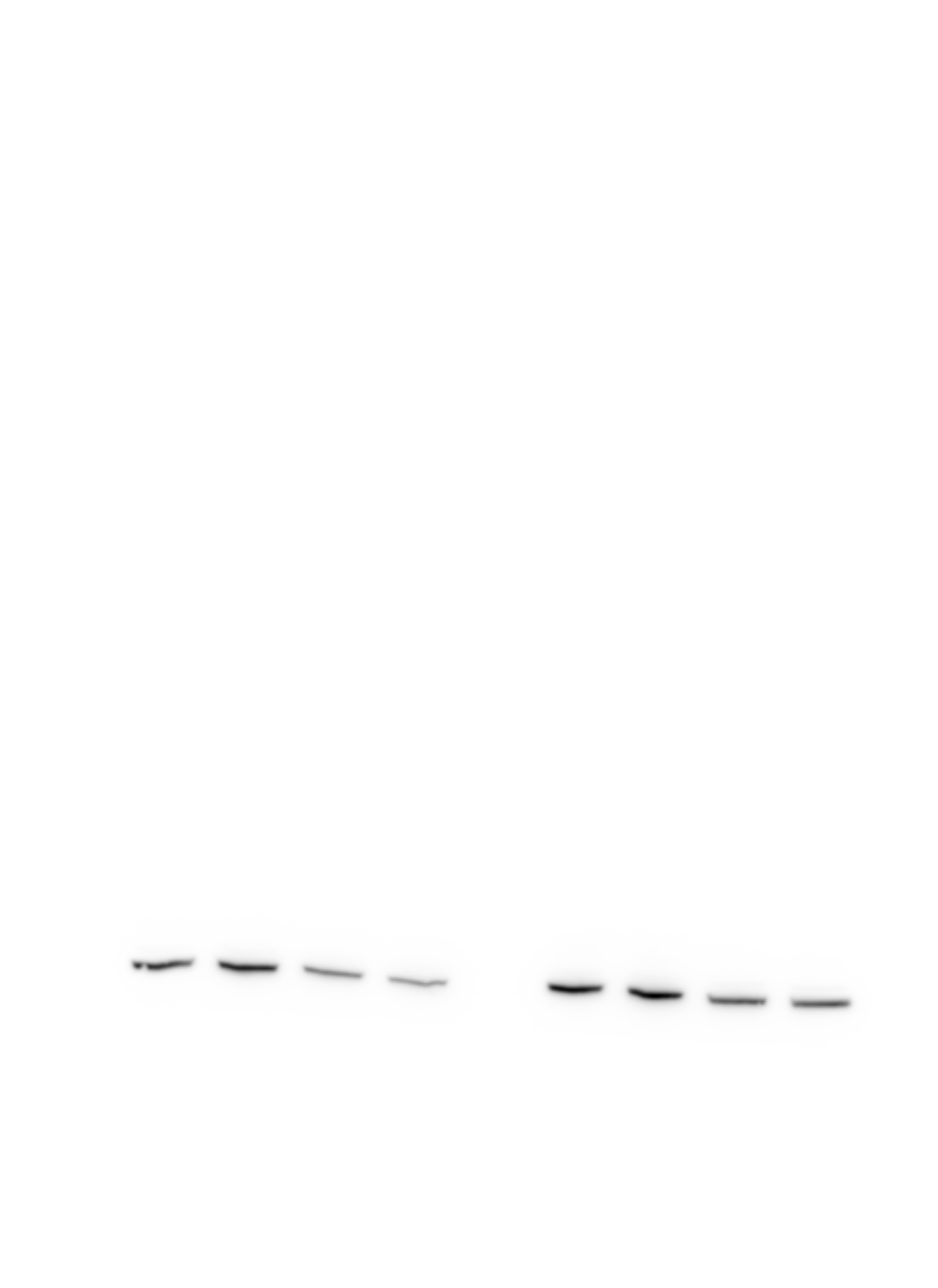

Supplement: Figure 3—source data 3. [file elife-101702-fig3-data3.zip › vinculin for p-NF-kB day 5.tif]

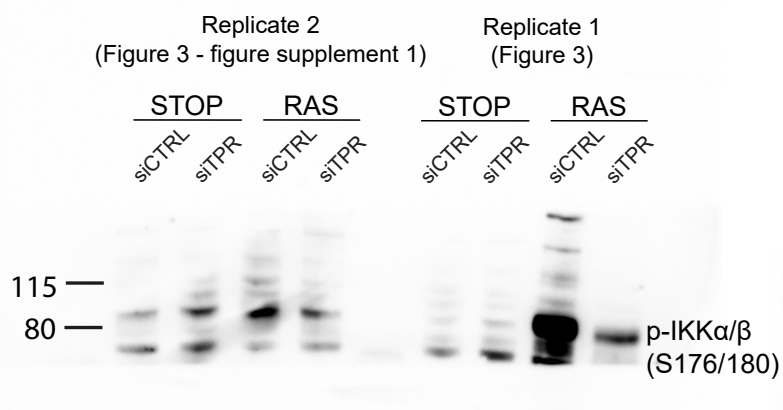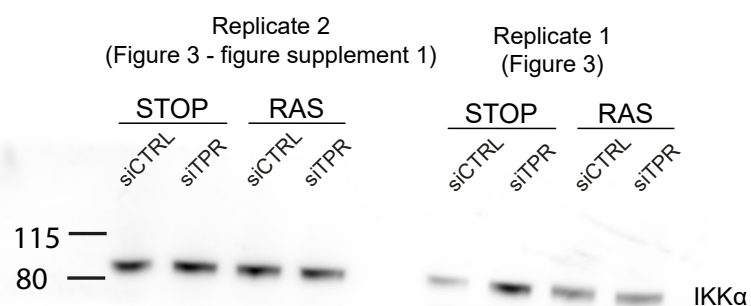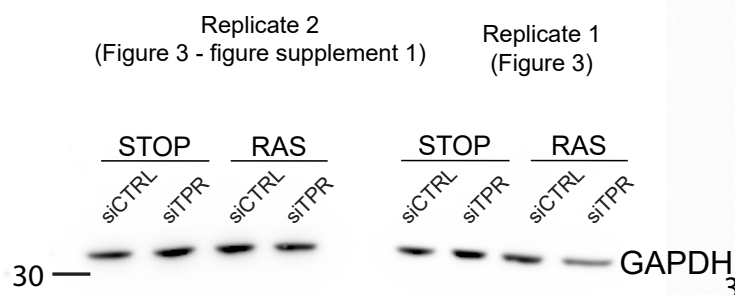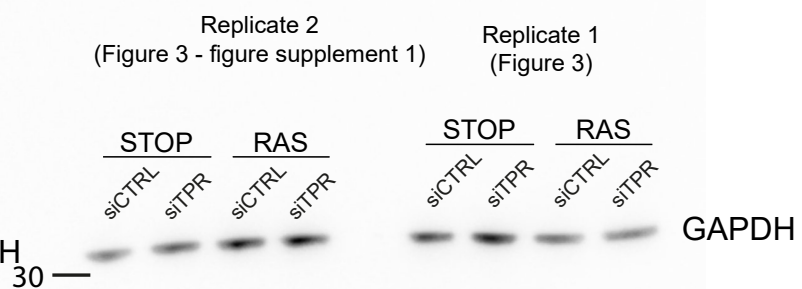

Supplement: Figure 3—figure supplement 1—source data 1. [file elife-101702-fig3-figsupp1-data1.zip › Figure 3 - figure supplement 1 uncropped IKK day 3.pdf]

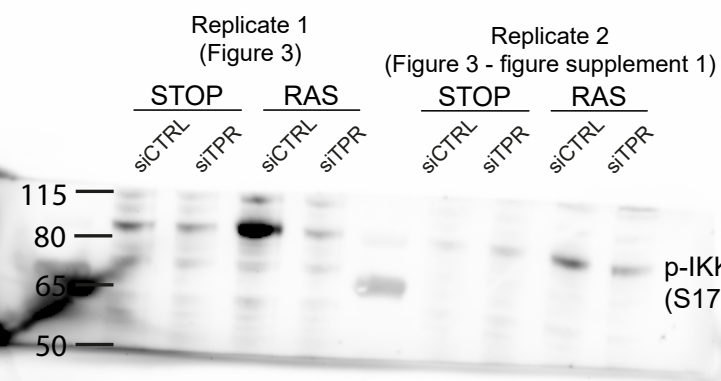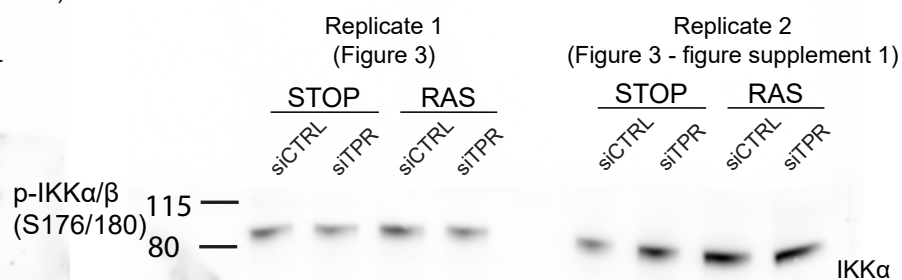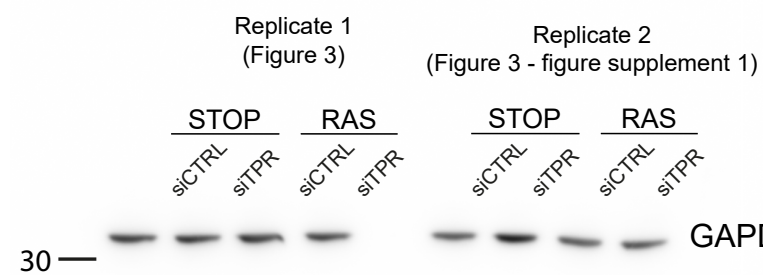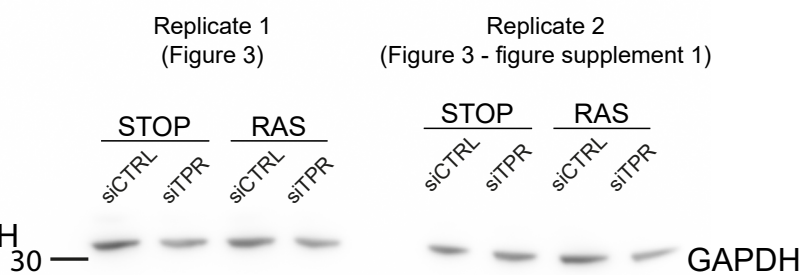

Supplement: Figure 3—figure supplement 1—source data 1. [file elife-101702-fig3-figsupp1-data1.zip › Figure 3 - figure supplement 1 uncropped IKK day 5.pdf]

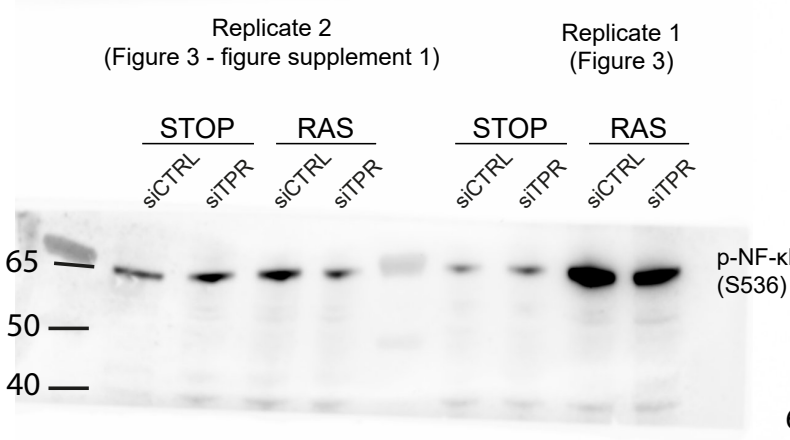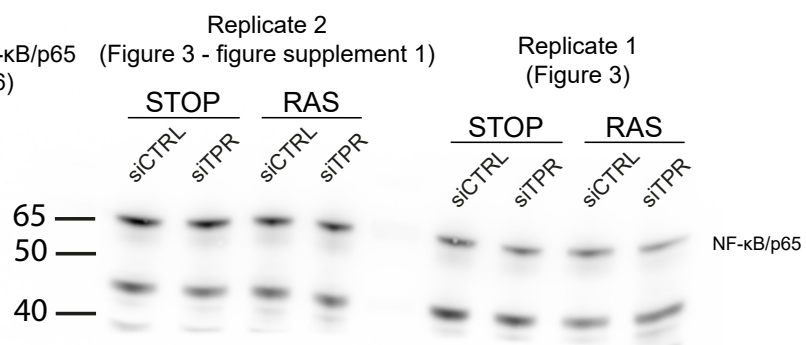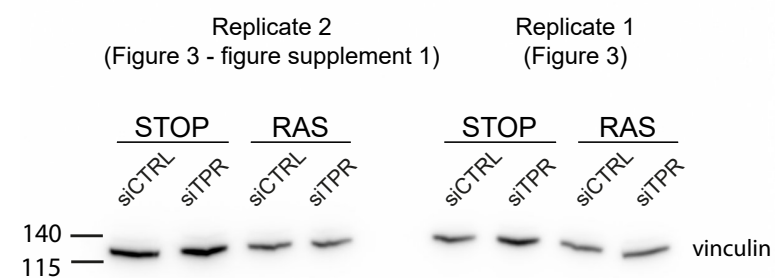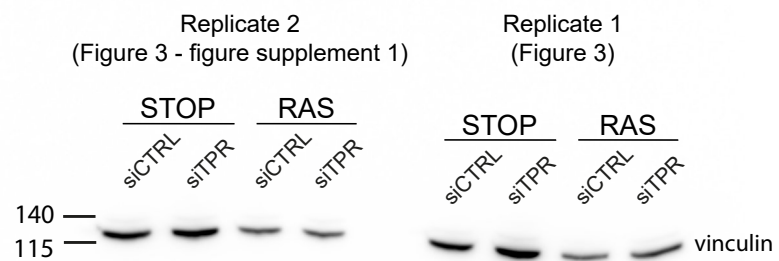

Supplement: Figure 3—figure supplement 1—source data 1. [file elife-101702-fig3-figsupp1-data1.zip › Figure 3 - figure supplement 1 Uncropped NF-kB day 3 blots.pdf]

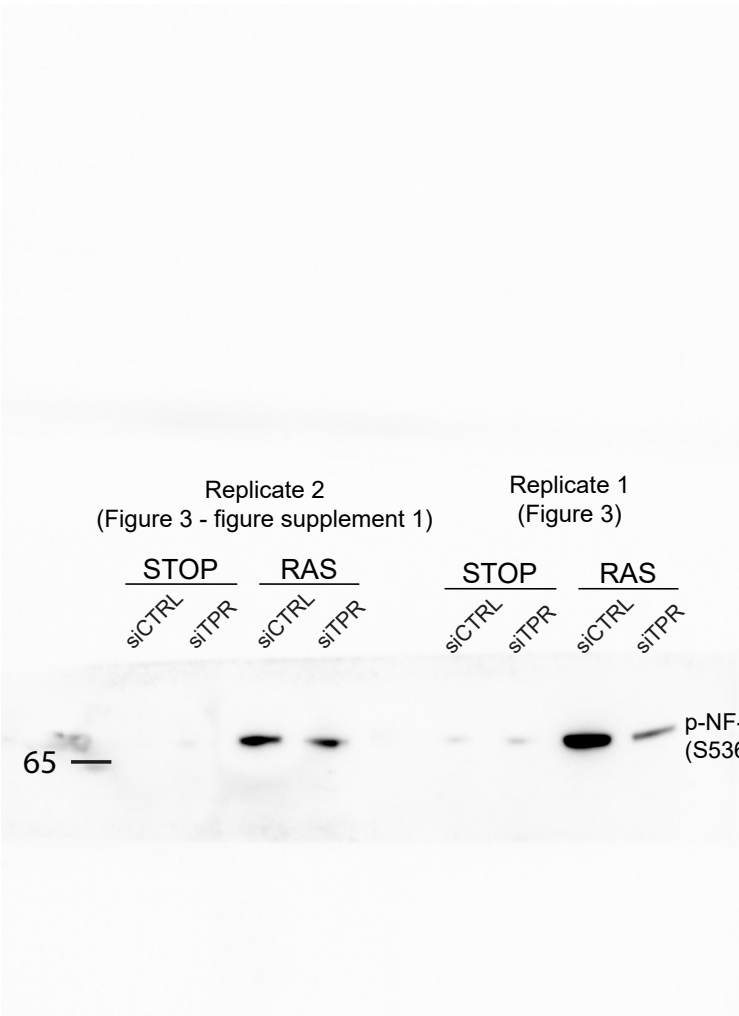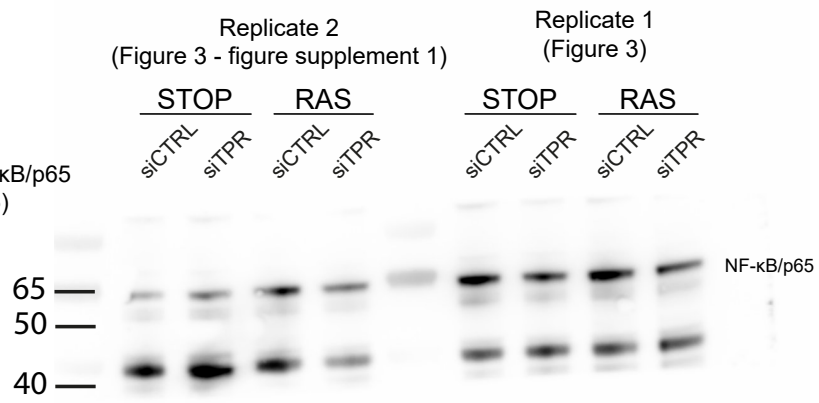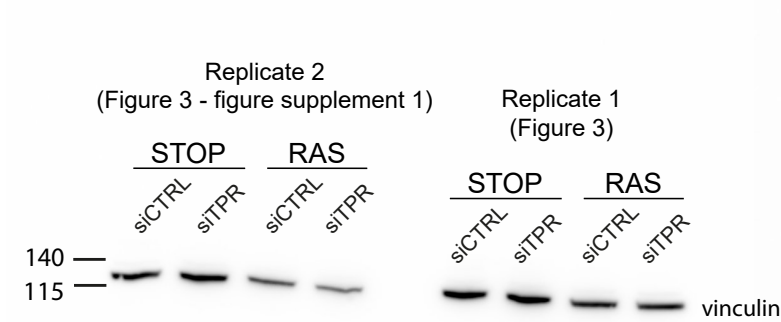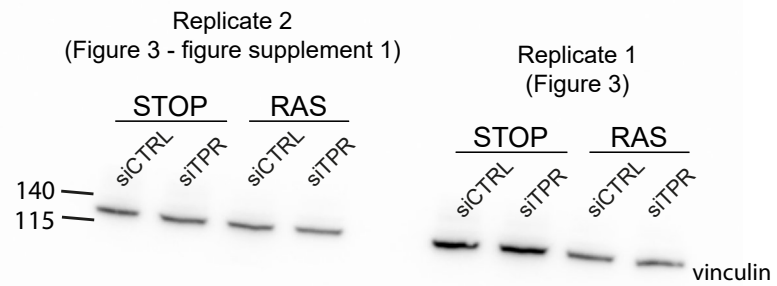

Supplement: Figure 3—figure supplement 1—source data 1. [file elife-101702-fig3-figsupp1-data1.zip › Figure 3 - figure supplement 1 Uncropped NF-kB day 5 blots.pdf]

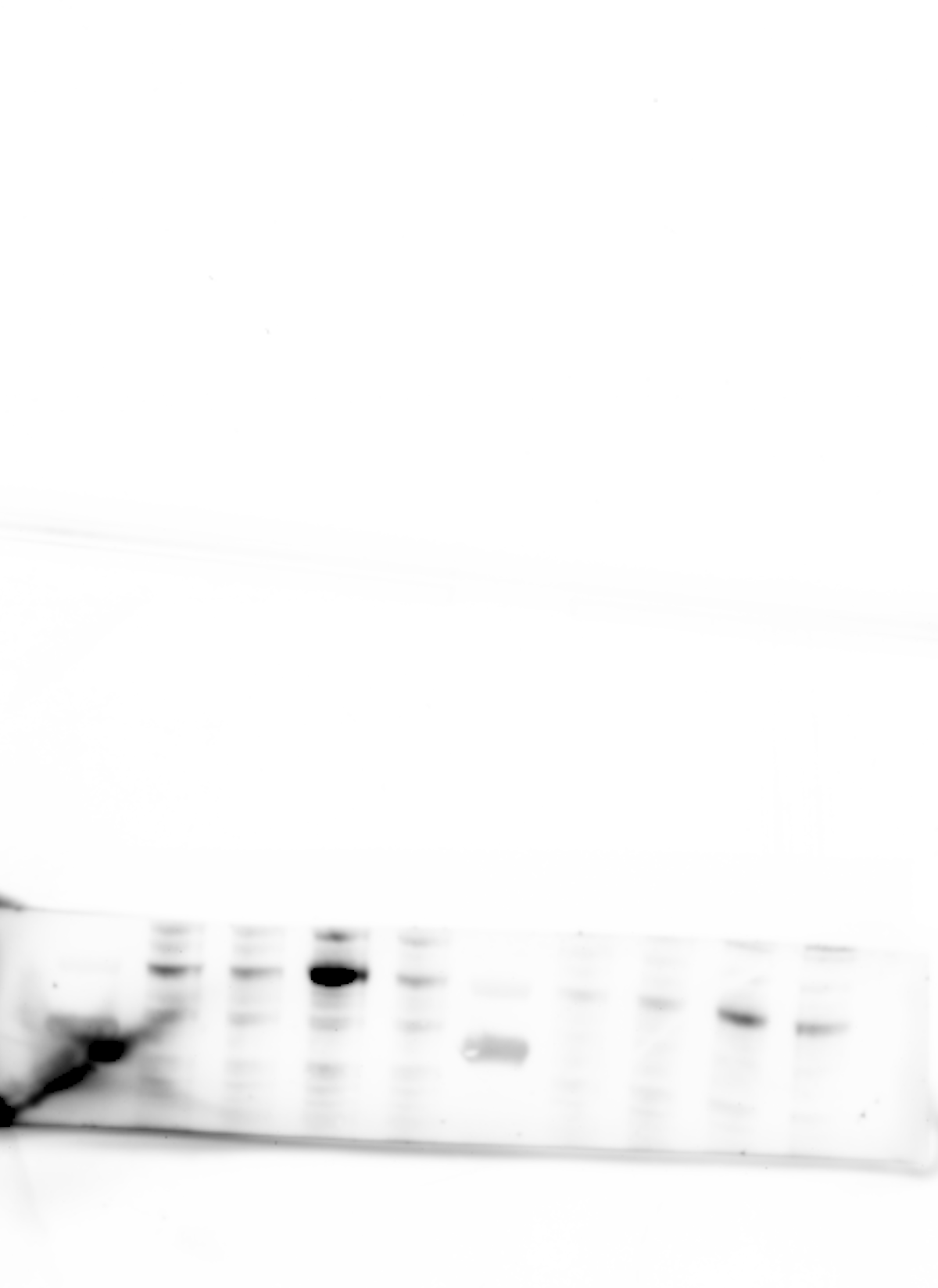

Supplement: Figure 3—figure supplement 1—source data 2. [file elife-101702-fig3-figsupp1-data2.zip › p-IKK day 5 rep2.tif]

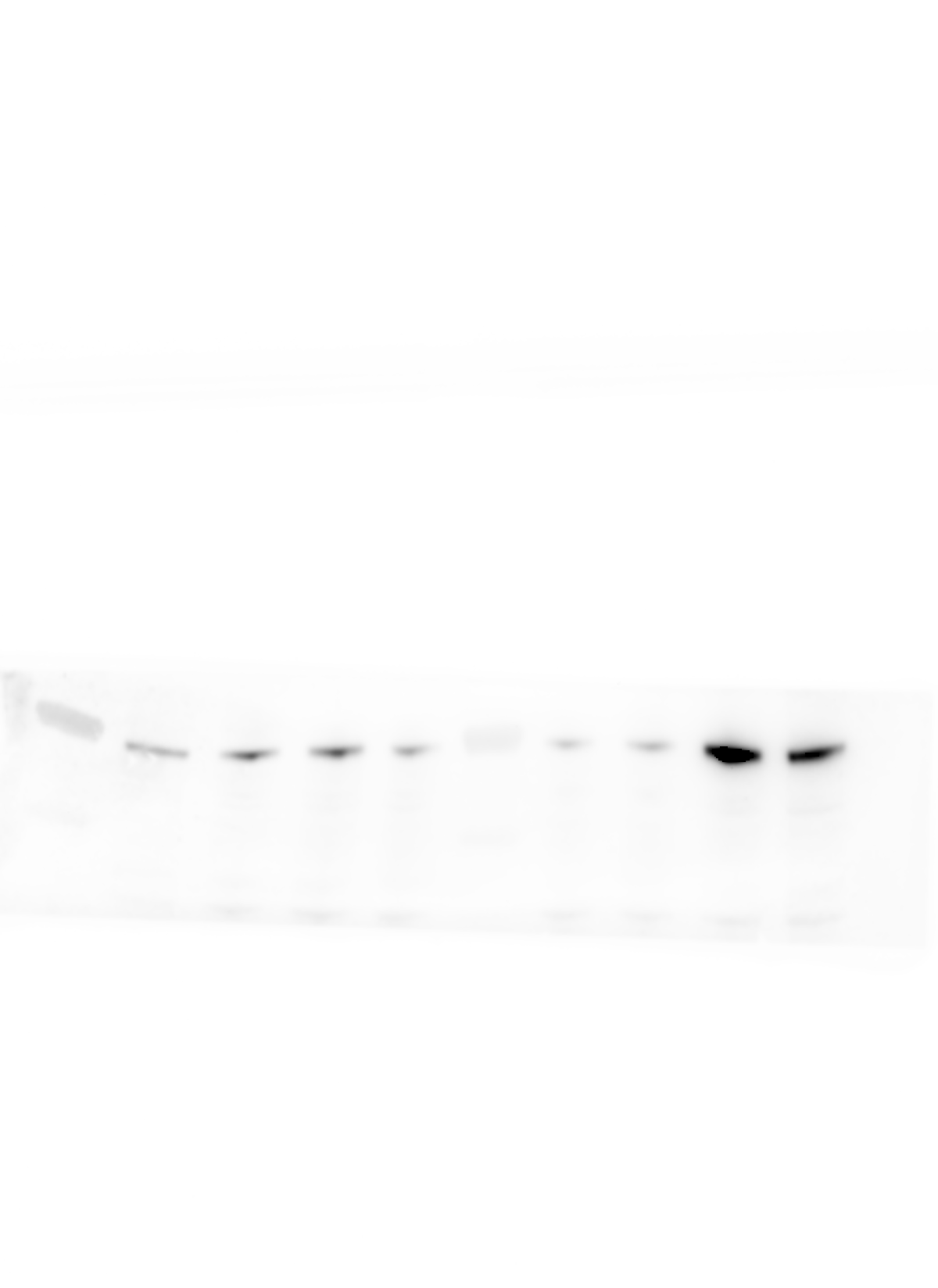

Supplement: Figure 3—figure supplement 1—source data 2. [file elife-101702-fig3-figsupp1-data2.zip › p-NF-kB day 3.tif]

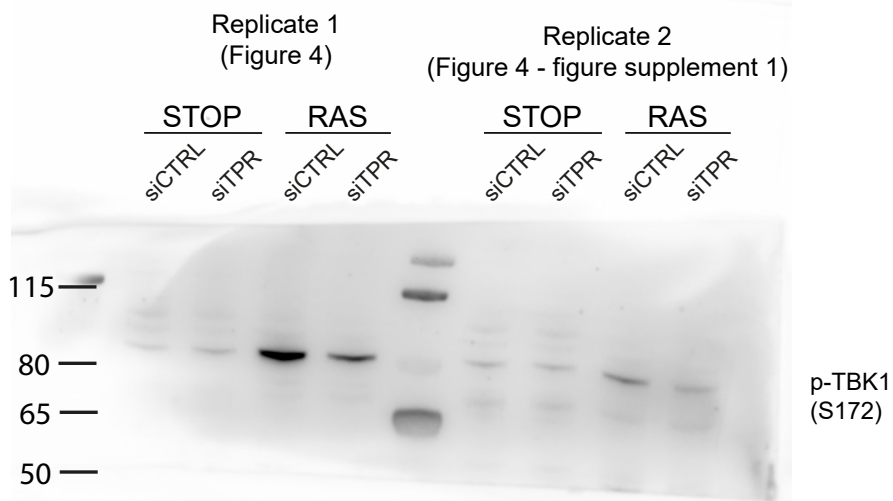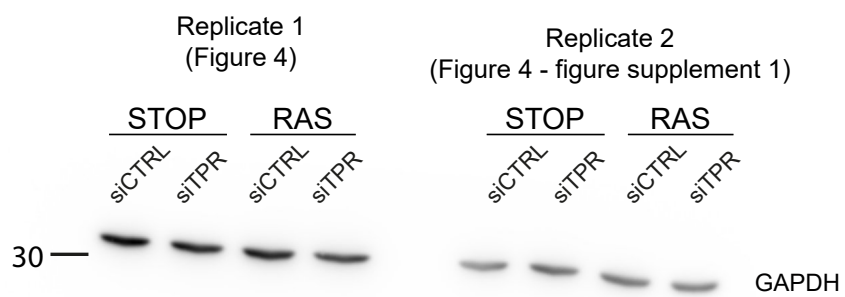

Supplement: Figure 4—source data 2. [file elife-101702-fig4-data2.zip › Figure 4 uncropped labelled p-TBK1 day 5.pdf]

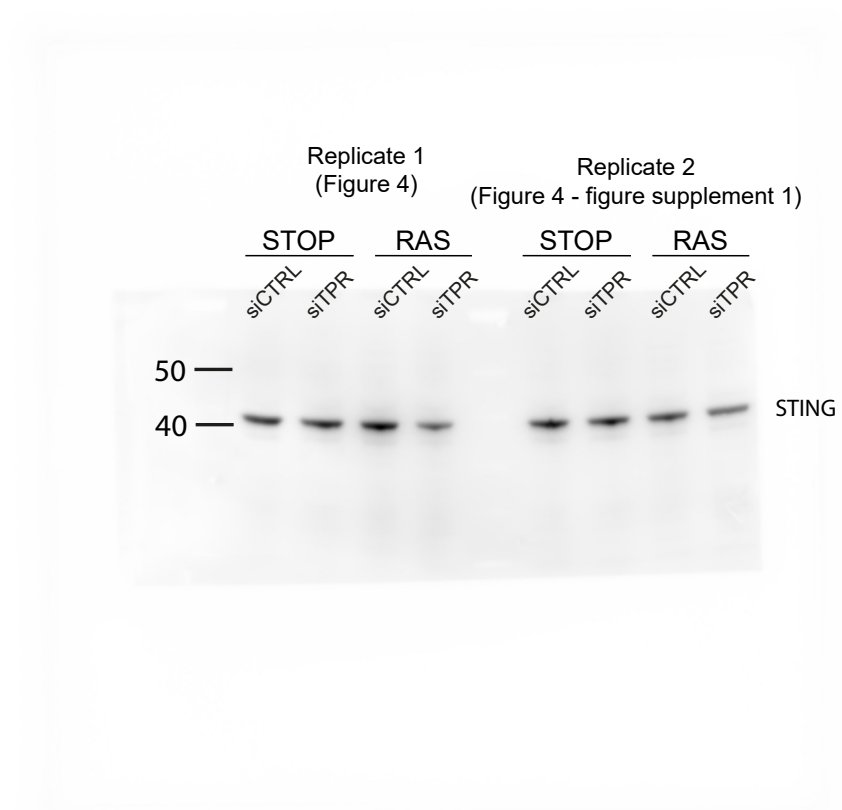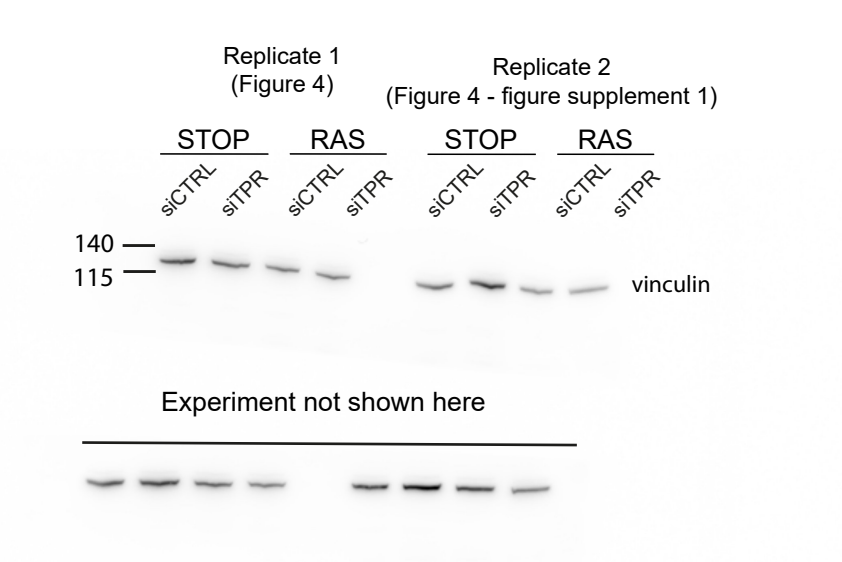

Supplement: Figure 4—source data 2. [file elife-101702-fig4-data2.zip › Figure 4 uncropped labelled STING day 3.pdf]

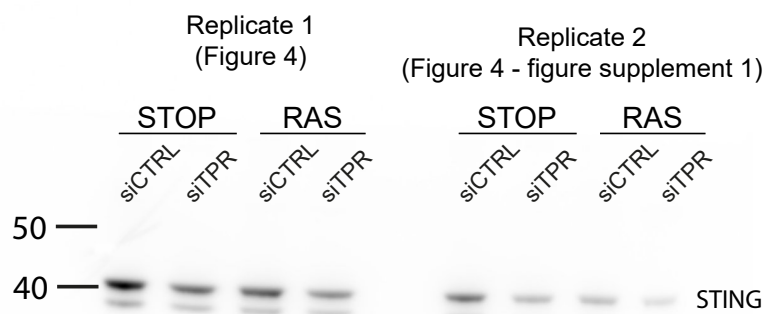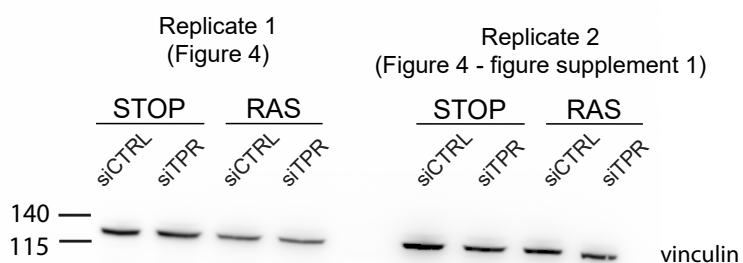

Supplement: Figure 4—source data 2. [file elife-101702-fig4-data2.zip › Figure 4 uncropped labelled STING day 5.pdf]

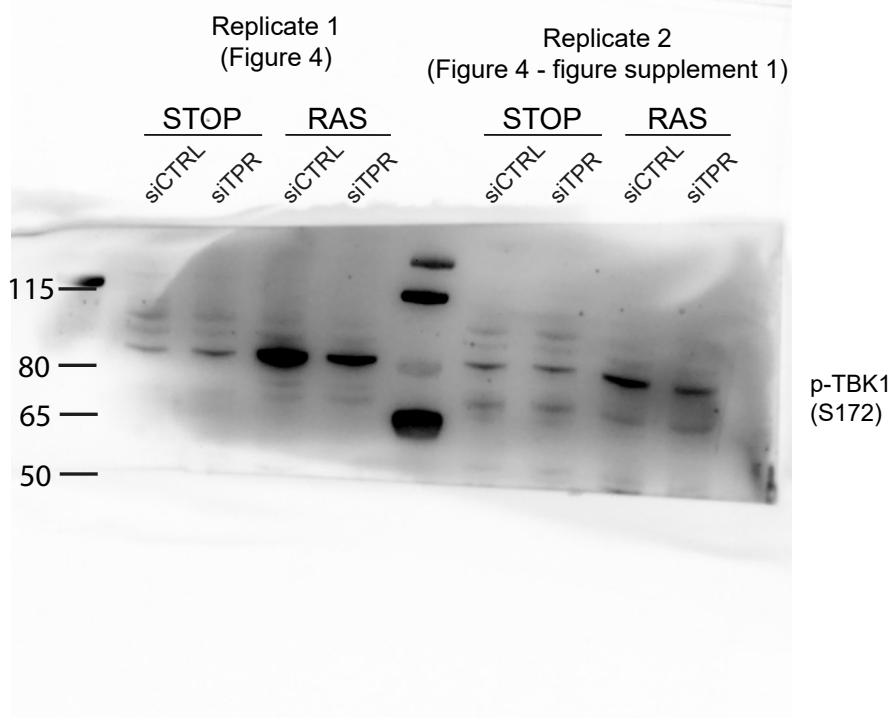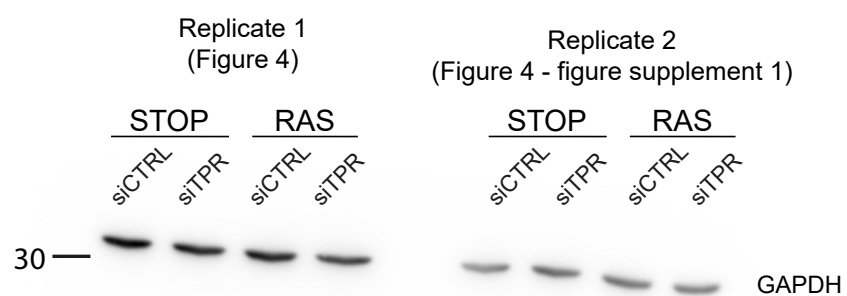

Supplement: Figure 4—figure supplement 1—source data 1. [file elife-101702-fig4-figsupp1-data1.zip › Figure 4 - figure supplement 1 uncropped labelled p-TBK1 day 5.pdf]

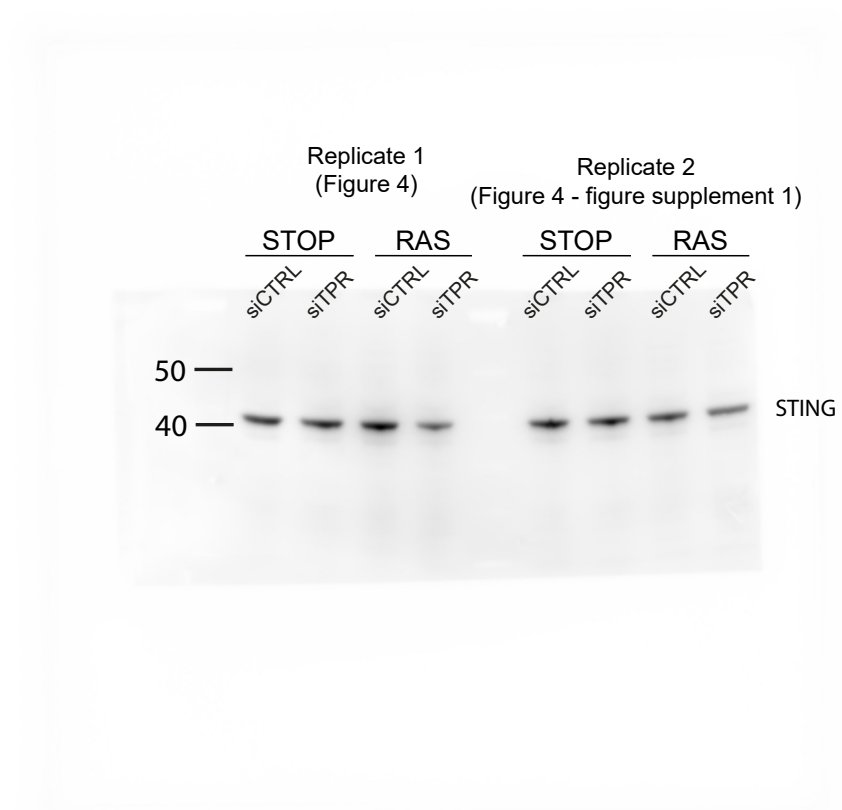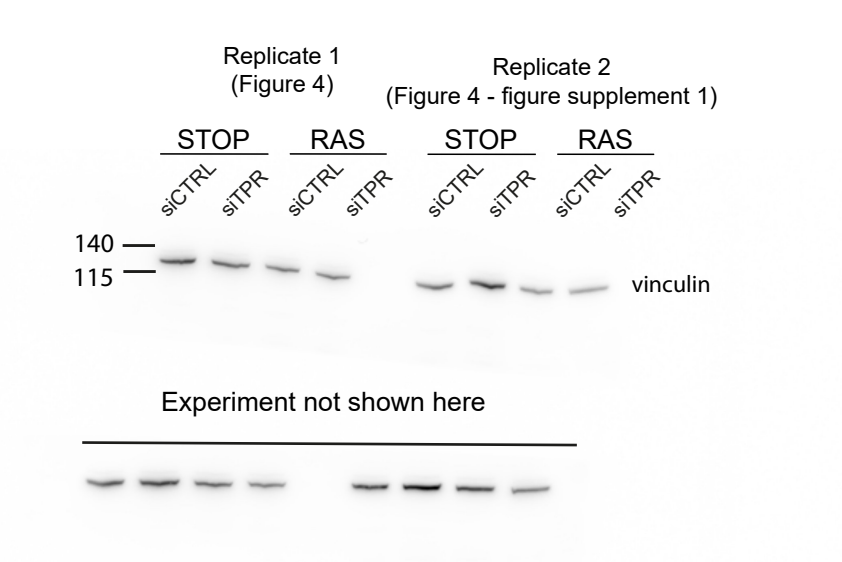

Supplement: Figure 4—figure supplement 1—source data 1. [file elife-101702-fig4-figsupp1-data1.zip › Figure 4 - figure supplement 1 uncropped labelled STING day 3.pdf]

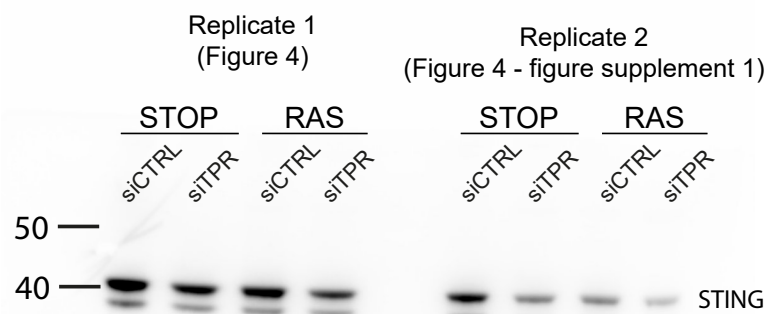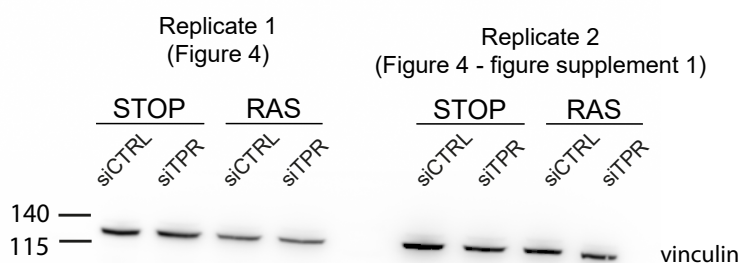

Supplement: Figure 4—figure supplement 1—source data 1. [file elife-101702-fig4-figsupp1-data1.zip › Figure 4 - figure supplement 1 uncropped labelled STING day 5.pdf]
