# Supplementary material for: TPR is required for cytoplasmic chromatin fragment formation during senescence
Source: eLife. 2024 Dec 3;13:e101702. doi: 10.7554/eLife.101702 (PMC11666244; doi:10.7554/eLife.101702)
Supplement: Figure 3—source data 1. — Median NF-κB nucleocytoplasmic ratios (n/c) and number of cells analysed for day 3 (d3) and d5 STOP or RAS cells subject to knockdown with control (CTRL) or TPR siRNAs. Kruskal-Wallis testing was used to determine statistical significance (p-value in parentheses) followed by Dunn’s post hoc testing. p-Values after Benjamini and Hochberg correction. NA: Kruskal-Wallis test showed no significant differences so it is not appropriate to carry out pairwise testing. [file elife-101702-fig3-data1.docx]

| **Figure 3C and Figure 3- figure supplement 1 A** | | | | | | | |
| --- | --- | --- | --- | --- | --- | --- | --- |
| **Cells** | **siRNA** | **NF-κB n/c** | **No. cells** | **P value** | **NF-κB n/c** | **No. cells** | **P value** |
|  |  | Replicate 1 (1.8E-04) | | | Replicate 2 (2.07E-13) | | |
| d3 STOP | siCTRL | 0.800 | 281 | 0.1599 | 0.730 | 263 | 2.84E-04 |
|  | siTPR | 0.787 | 207 |  | 0.765 | 189 |  |
| RAS | siCTRL | 0.837 | 195 | 0.0850 | 0.807 | 181 | 0.583 |
|  | siTPR | 0.806 | 200 |  | 0.814 | 152 |  |
| STOP | siCTRL | 0.800 | 281 | 0.0054 | 0.730 | 263 | 3.80E-10 |
| RAS |  | 0.837 | 195 |  | 0.807 | 181 |  |
|  |  | Replicate 1 (0.538) | | | Replicate 2 (6.37E-06) | | |
| d5 STOP | siCTRL | 0.811 | 232 | NA | 0.792 | 184 | 0.67 |
|  | siTPR | 0.825 | 188 |  | 0.785 | 160 |  |
| RAS | siCTRL | 0.821 | 218 | NA | 0.787 | 205 | 9.92E-05 |
|  | siTPR | 0.826 | 156 |  | 0.847 | 173 |  |
| STOP | siCTRL | 0.811 | 232 | NA | 0.792 | 184 | 0.92 |
| RAS |  | 0.821 | 218 |  | 0.787 | 205 |  |

| **Figure 3D and Figure 3- figure supplement 1 B** | | | | | | | |
| --- | --- | --- | --- | --- | --- | --- | --- |
| **Cells** | **siRNA** | **NF-κB** | **No. cells** | **P value** | **NF-κB** | **No. cells** | **P value** |
|  |  | Replicate 1 (< 2.2E-16) | | | Replicate 2 (< 2.2E-16) | | |
| d3 STOP | siCTRL | 0.019 | 281 | 3.36E-14 | 0.025 | 263 | 2.97E-03 |
|  | siTPR | 0.027 | 207 |  | 0.028 | 189 |  |
| RAS | siCTRL | 0.055 | 195 | 2.69E-04 | 0.052 | 181 | 4.93E-05 |
|  | siTPR | 0.044 | 200 |  | 0.040 | 152 |  |
| STOP | siCTRL | 0.019 | 281 | 1.39E-110 | 0.025 | 263 | 3.59E-73 |
| RAS |  | 0.055 | 195 |  | 0.052 | 181 |  |
|  |  | Replicate 1 (< 2.2E-16) | | | Replicate 2 (< 2.2E-16) | | |
| d5 STOP | siCTRL | 0.028 | 232 | 6.51E-06 | 0.034 | 184 | 0.209 |
|  | siTPR | 0.024 | 188 |  | 0.032 | 160 |  |
| RAS | siCTRL | 0.050 | 218 | 7.60E-34 | 0.053 | 205 | 4.46E-04 |
|  | siTPR | 0.029 | 156 |  | 0.046 | 173 |  |
| STOP | siCTRL | 0.028 | 232 | 1.71E-44 | 0.034 | 184 | 3.90E-34 |
| RAS |  | 0.050 | 218 |  | 0.053 | 205 |  |

**Figure 3 – source data 1. Quantification of NF-κB nucleocytoplasmic ratios, nuclear intensity and statistical analysis for data in Figure 3C,D and for biological replicates in Figure 3 - figure supplement 1A and B.** Median NF-κB nucleocytoplasmic ratios (n/c) and number of cells analysed for d3 and d5 STOP or RAS cells subject to knockdown with control (CTRL) or TPR siRNAs. Kruskal-Wallis testing was used to determine statistical significance (p value in parentheses) followed by Dunn post-hoc testing. P values after Benjamini and Hochberg correction. NA: Kruskal-Wallis test showed no significant differences so it is not appropriate to carry out pairwise testing.
