# Supplementary material for: TPR is required for cytoplasmic chromatin fragment formation during senescence
Source: eLife. 2024 Dec 3;13:e101702. doi: 10.7554/eLife.101702 (PMC11666244; doi:10.7554/eLife.101702)
Supplement: Figure 4—source data 1. — One-way ANOVA was used to determine statistical significance followed by Šídák’s multiple comparisons test. [file elife-101702-fig4-data1.docx]

| **Figure 4B** | P value | Summary |
| --- | --- | --- |
| ANOVA | 0.0141 | * |
| Šídák's multiple comparisons test | Adjusted P Value | Summary |
| STOP siCTRL vs. STOP siTPR | 0.6882 | ns |
| STOP siCTRL vs. RAS siCTRL | 0.6959 | ns |
| RAS siCTRL vs. RAS siTPR | 0.0476 | * |

| **Figure 4D** | P value | Summary |
| --- | --- | --- |
| ANOVA | 0.0108 | * |
| Šídák's multiple comparisons test | Adjusted P Value | Summary |
| STOP siCTRL vs. STOP siTPR | 0.9333 | ns |
| STOP siCTRL vs. RAS siCTRL | 0.0109 | * |
| STOP siCTRL vs. RAS siTPR | 0.1124 | ns |
| RAS siCTRL vs. RAS siTPR | 0.6315 | ns |
| STOP siTPR vs RAS siTPR | 0.1734 | ns |

**Figure 4 – source data 1. Statistical analysis for *STING1* qPCR data in Figure 4B and for cGAMP ELISA data in Figure 4D.** One-way ANOVA was used to determine statistical significance followed by Šídák's multiple comparisons test.
