# Supplementary material for: TPR is required for cytoplasmic chromatin fragment formation during senescence
Source: eLife. 2024 Dec 3;13:e101702. doi: 10.7554/eLife.101702 (PMC11666244; doi:10.7554/eLife.101702)
Supplement: Figure 5—source data 1. — Data were fitted to a generalised linear model before carrying out pairwise comparisons between samples. 500 cells were assessed per sample for each replicate of each experiment. [file elife-101702-fig5-data1.docx]

| Figure 5A | | |
| --- | --- | --- |
| % cells with CCFs | Day 3 p value | Day 5 p value |
| STOP siCTRL vs. STOP siTPR | 1 | 0.9931 |
| STOP siCTRL vs. RAS siCTRL | <0.0001 | 0.0214 |
| RAS siCTRL vs. RAS siTPR | 0.1848 | <.0001 |

| Figure 5G | |
| --- | --- |
| % cells with SAHF | p value |
| STOP siCTRL vs. RAS siCTRL | <0.0001 |
| RAS siCTRL vs. RAS siTPR | <0.0001 |
| RAS siCTRL vs. RAS siHMGA1 | <0.0001 |
| RAS siTPR vs. RAS siHMGA1 | <0.0001 |

| Figure 5H | |
| --- | --- |
| % cells with CCFs | p value |
| STOP siCTRL vs. RAS siCTRL | 0.0002 |
| RAS siCTRL vs. RAS siTPR | 0.0488 |
| RAS siCTRL vs. RAS siHMGA1 | 0.0172 |
| RAS siTPR vs. RAS siHMGA1 | 0.9806 |

**Figure 5 – source data 1. Statistical analysis for CCF and SAHF data in Figure 5A, G and H.** Data were fitted to a generalised linear model before carrying out pairwise comparisons between samples**.** 500 cells were assessed per sample for each replicate of each experiment.
