# Supplementary material for: TPR is required for cytoplasmic chromatin fragment formation during senescence
Source: eLife. 2024 Dec 3;13:e101702. doi: 10.7554/eLife.101702 (PMC11666244; doi:10.7554/eLife.101702)
Supplement: Supplementary file 1. — (a) Table summarising day 8 ATAC-seq changes in peak accessibility. Number of ATAC-peaks with significant changes generated from comparisons between samples using the limma package with an adjusted p-value cut-off of 0.05. Peaks significantly upregulated in RAS siCTRL compared to STOP siCTRL (SEN+) were further divided into TPR-dependent (SEN+TPR+) and TPR-independent (SEN+TPR-) as shown. (b) Table indicating the proximity of senescence-associated secretory phenotype (SASP) gene promoters to TPR-dependent, senescence-dependent ATAC-seq peaks. Distance (in bp) between TPR+SEN+ ATAC-seq peaks from the transcription start site (TSS) of genes involved in positive regulation of the inflammatory response, cytokine activity, and cytokine receptors. (c) Table summarising day 3 ATAC-seq changes in peak accessibility. Number of peaks with significant changes generated from comparisons between samples using the limma package with an adjusted p-value cut-off of 0.05. [file elife-101702-supp1.docx]

| **Comparison** | **No. Peaks with significant changes** |  |  |
| --- | --- | --- | --- |
| STOP siCTRL  vs  RAS siCTRL | 15732 | Down in RAS siCTRL: 8906 |  |
|  |  | Up in RAS siCTRL: 6826 | Down in RAS siTPR  (SEN^+^TPR^+^): 1187 |
|  |  |  | No change in RAS siTPR  (SEN^+^TPR-): 5639 |
|  |  |  | Up in RAS siTPR: 0 |
| STOP siCTRL  vs  STOP siTPR | 0 |  |  |
| RAS siCTRL  vs  RAS siTPR | 6185 | Down in RAS siTPR: 3972 |  |
|  |  | Up in RAS siTPR: 2213 |  |

**Supplementary file 1a. Table summarising day 8 ATAC-seq changes in peak accessibility.** Number of ATAC-peaks with significant changes generated from comparisons between samples using the limma package with an adjusted p-value cut-off of 0.05. Peaks significantly upregulated in RAS siCTRL compared to STOP siCTRL (SEN^+^) were further divided into TPR-dependent (SEN^+^TPR^+^) and TPR-independent (SEN^+^TPR^-^) as shown.

| Gene | Distance (bp) of TPR+ SEN+ peak from TSS |
| --- | --- |
| **Positive regulation of inflammatory response** | |
| *AGTR1* | -467229 |
| *EGFR* | -58375 |
| *ETS1* | -17373, +7357, +276923, +568579, +661894, +967169 |
| *IL1B* | -2652, +7134, +12164 |
| *IL33* | -144624 |
| *IL6* | -115454 |
| *IL6ST* | -344864 |
| *ITGA2* | +80370 |
| *KLKB1* | +33313 |
| *LBP* | +104427 |
| *MGST2* | +225388 |
| *NFKBIA* | +84158 |
| *PDCD4* | -148065 |
| *PDE2A* | +190585 |
| *PTGER4* | -203706 |
| *TLR2* | -39731 |
| *TLR6* | +34849 |
| *TLR9* | +24870 |
| *TNFSF11* | -99106, -49127, +319075 |
| *TNFSF18* | -99190, -61362 |
| *TNFSF4* | -135539, +57159, +94987 |
| *WNT5A* | -134541, +331390, +332410, +345547 |
| **Cytokine activity** | |
| *AREG* | +101059 |
| *AREGB* | -68719, +76171 |
| *BMP2* | -207544, +9230, +14328, +15362 |
| *BMP4* | -244195 |
| *BMP7* | +226902 |
| *CCL16* | +3017 |
| *CXCL6* | -94445, -91993 |
| *EDN1* | +52879 |
| *GDF6* | +694339 |
| *GDF7* | +20647 |
| *GREM1* | -30294, +115581, +125600, +263759 |
| *GREM2* | -621447 |
| *IFNE* | -200915, -41407 |
| *IL13* | -63993 |
| *IL19* | +53062 |
| *IL1A* | -45179, -40149 |
| *IL1B* | -2652, +7134, +12164 |
| *IL20* | -13422 |
| *IL22* | -51312 |
| *IL33* | -144624 |
| *IL6* | -115454 |
| *IL8* | -18679, -17284, +1546, +3998 |
| *INHBA* | +191789, +464037, +478566 |
| *INHBB* | +346976 |
| *KITLG* | -655937, -576588 |
| *NAMPT* | -86921, +94455 |
| *NRG1* | -118046, +26745, +27970, +164917, +545062 |
| *SPP1* | -79441 |
| *TGFB2* | +118699 |
| *TNFRSF11B* | -93919 |
| *TNFSF10* | -112408 |
| *TNFSF11* | -99106, -49127, +319075 |
| *TNFSF15* | -77809, -14960, +21708 |
| *TNFSF18* | -99190, -61362 |
| *TNFSF4* | -135539, +57159, +94987 |
| *TNFSF8* | +46482, +109331 |
| *WNT5A* | -134541, +331390, +332410, +345547 |
| **Cytokine receptor binding** | |
| *BAMBI* | +166771, +206795, +237605 |
| *BMP2* | -207544, +9230, +14328, +15362 |
| *BMP4* | -244195 |
| *BMP7* | +226902 |
| *CASP3* | +139376 |
| *CCL16* | +3017 |
| *CXCL6* | -94445, -91993 |
| *FKBP1A* | -25370 |
| *FRS2* | +99292 |
| *GDF6* | +694339 |
| *GDF7* | +20647 |
| *GREM1* | -30294, +115581, +125600, +263759 |
| *IFNE* | -200915, -41407 |
| *IL13* | -63993 |
| *IL1A* | -45179, -40149 |
| *IL1B* | -2652, +7134, +12164 |
| *IL20* | -13422 |
| *IL22* | -51312 |
| *IL6* | -115454 |
| *IL6R* | -57849 |
| *IL6ST* | -344864 |
| *IL8* | -18679, -17284, +1546, +3998 |
| *INHBA* | +191789, +464037, +478566 |
| *INHBB* | +346976 |
| *JAK1* | -116968 |
| *KITLG* | -655937, -576588 |
| *LIFR* | +180867 |
| *NGF* | -115612, -114631 |
| *PIK3R1* | -912093, -909082, -784284 |
| *PLCG1* | +150309 |
| *RASL11B* | +206839 |
| *SMAD7* | -259928, -259255, +44985, +46429, +223976 |
| *TGFB2* | +118699 |
| *TGFBR2* | -443985 |
| *TLR9* | +24870 |
| *TNFSF10* | -112408 |
| *TNFSF11* | -99106, -49127, +319075 |
| *TNFSF15* | -77809, -14960, +21708 |
| *TNFSF18* | -99190, -61362 |
| *TNFSF4* | -135539, +57159, +94987 |
| *TNFSF8* | +46482, +109331 |
| *TRAF3* | +9481 |
| *VEGFC* | -220648, +153612 |

**Supplementary file 1b. Table indicating the proximity of SASP gene promoters to TPR-dependent, senescence-dependent ATAC-seq peaks**. Distance (in bp) between TPR^+^SEN^+^ ATAC-seq peaks from the transcription start site (TSS) of genes involved in positive regulation of the inflammatory response, cytokine activity and cytokine receptors.

| **Comparison** | **No. Peaks with significant**  **changes** |  |
| --- | --- | --- |
| STOP siCTRL  vs  RAS siCTRL | 94394 | Down in RAS siCTRL: 58200 |
|  |  | Up in RAS siCTRL: 36194 |
| STOP siCTRL  vs  STOP siTPR | 0 |  |
| RAS siCTRL  vs  RAS siTPR | 0 |  |

**Supplementary file 1c. Table summarising day 3 ATAC-seq changes in peak accessibility.** Number of peaks with significant changes generated from comparisons between samples using the limma package with an adjusted p-value cut-off of 0.05.
